# Functional Screen of Wilson Disease ATP7B Variants Reveals Residual Transport Activities

**DOI:** 10.1155/humu/7485658

**Published:** 2025-07-07

**Authors:** Jenifer S. Calvo, Tomáš Heger, Ekaterina Kabin, William R. Mowrey, Guillermo Del Angel, Wei Ding, Svetlana Lutsenko

**Affiliations:** ^1^Department of Physiology, Johns Hopkins Medical Institutes, Baltimore, Maryland, USA; ^2^Department of Molecular Biology and Genetics, Danish Research Institute of Translational Neuroscience (DANDRITE), Nordic EMBL Partnership for Molecular Medicine, Aarhus University, Aarhus, Denmark; ^3^Alexion, AstraZeneca Rare Disease, Boston, Massachusetts, USA

**Keywords:** ATP7B, copper, mutations, variants, Wilson disease

## Abstract

Wilson disease is a disorder of copper (Cu) homeostasis caused by the malfunction of Cu transporter ATP7B and associated Cu accumulation in tissues. The existence of over 700 disease-associated variants in the *ATP7B* gene and a broad spectrum of disease manifestations complicate the analysis of genotype–phenotype correlations and the development of better treatments for this disorder. To assist such studies, we screen 101 variants of ATP7B for expression and Cu transport activity in human fibroblasts lacking active ATP-dependent Cu transporters. The ClinVar database classified 59 of these as variants of uncertain significance or having conflicting pathogenicity classifications; six variants were not in the database. Thirty-three of the variants have been previously characterized by other assays. Only three variants (S657R, G1061E, and G1266R) resulted in the complete inactivation of Cu transport. The in silico analysis of these mutants was used to rationalize this drastic effect on ATP7B activity. The remaining ATP7B variants showed a range of Cu transport activities. Coexpression of variants with different properties yielded activity values different from the simple average. The advantages and limitations of this functional screen are discussed.

## 1. Introduction

Wilson disease (WD) is a disorder associated with cellular copper (Cu) overload, predominantly in the liver and the brain, resulting in tissue damage and neurological dysfunction [1, 2]. Cu accumulation is caused by mutations in the gene encoding the Cu transporter ATP7B and the loss of its function. ATP7B is a large multidomain membrane protein. It has 8 transmembrane (TM) domains. In the TM domains, conserved charged residues in TMs 3 and 4 form the Cu entry site into the Cu transport pathway. The CPC motif in TM6 is the Cu binding site, while histidines in the TM1,2 loop and TM5,6 loop facilitate Cu release [3–6]. The six cytosolic N-terminal metal binding domains (MBDs) accept Cu from the Cu-chaperone Atox1 and regulate ATP7B activity and trafficking [7–12]. The cytosolic N- and P-domains are the sites of ATP binding/hydrolysis and catalytic phosphorylation, respectively. The TGE motif in the A-domain interacts with the P-domain during phosphorylation/dephosphorylation, rotating to cause rearrangement of the TM domains for Cu translocation [13]. The C-terminus is involved in Golgi retention and trafficking of ATP7B [14]. More than 700 variants in the *ATP7B* gene have been identified [15]; these variants decrease ATP7B expression, stability, transport activity, cellular localization, and/or Cu-dependent trafficking [16–25]. This large number of variants, the compound heterozygous nature of WD, and WD phenotypic variability make establishing the genotype–phenotype correlation necessary for precise diagnosis and treatment of WD very challenging [26–28].

Treatment options for WD include chelation therapy using D-penicillamine or trientine to promote Cu excretion and/or zinc supplementation to reduce Cu uptake [29]. While effective in addressing hepatic symptoms, these treatments are life-long, expensive, and paradoxically can trigger neurological deterioration [30]. Cell and gene therapies, which aim to permanently express functional ATP7B, have shown promise in animal studies but also present challenges such as enhanced immune response and tumor biogenesis [31–33]. Other potential strategies include the use of corrector molecules to stabilize ATP7B to alleviate the endoplasmic reticulum (ER) retention and/or activate autophagy [34, 35]. For “correctors” to be successful, functional characterization of the disease-causing variants is necessary to identify the most promising candidates with residual Cu transport. Several common pathogenic variants, such as H1069Q, have been previously characterized in detail. H1069Q-ATP7B was found to have residual activity, but in polarized hepatocytes, it was unable to traffic to the canalicular membrane and export Cu [36]. However, the majority of variants have not been characterized. Additionally, it has been shown that coexpression of different WD variants affects the functional characteristics of individual variants [23]. This property has not been well evaluated, as mutants have mostly been characterized in vitro and in cellular models as homozygous.

In this study, we characterized 101 WD-causing ATP7B variants by testing whether variants were expressed and capable of Cu transport using Menkes disease fibroblast cells (YST), which lack Cu export and accumulate Cu. The vast majority of tested ATP7B variants, when overexpressed, showed measurable Cu transport activity; the unambiguously inactive ATP7B mutants were also identified. Our results highlight the best uses and the limitations of screens based on functional complementation. The identification of a large number of ATP7B variants capable of Cu transport warrants the search for small “corrector” molecules to increase their activity.

## 2. Materials and Methods

### 2.1. Selection of Variants and Site-Directed Mutagenesis to Generate ATP7B Variants

One hundred one WD-associated ATP7B variants were used for this study; these variants are present in different domains of the protein. To ascertain the potential of the in vitro assay in correlating functional changes with the magnitude of clinical effects, missense variants were selected among three groups of ATP7B variants, namely, known benign polymorphic missense variants, known pathogenic missense variants, and missense variants of either uncertain significance, unknown effect, or conflicting interpretations of pathogenicity. Due to the very large number of variants, priority was given to more common variants listed in population databases such as gnomAD [37].

The plasmid expressing the N-terminally GFP-tagged wild-type human ATP7B (Figure S1) was used as a template to introduce desired site-specific mutations. In studies of Cu transport, the WT plasmid without mutations served to produce active ATP7B (positive control); the plasmid with the mutation converting catalytic Asp1027 to Ala (D1027A) and rendering ATP7B inactive served as a negative control. Site-directed mutagenesis and sequencing to verify the correct sequence were performed by GenScript (Piscataway, NJ).

### 2.2. Transfection of Menkes Fibroblast Cells (YST)

Menkes fibroblast cells (YST), a human cell line that lacks active Cu transporters ATP7A and ATP7B [38, 39], were transfected with plasmids expressing WT ATP7B or D1027A variant or ATP7B mutant of interest. The method of Roy et al. [23] was used for the characterization of ATP7B expression and activity with the following modifications: the format was scaled down from six-well (10 cm^2^) plates to eight-well (0.8 cm^2^) chamber slides to increase throughput and decrease the amount of required plasmid, and the amount of plasmid per well was reduced to avoid strong overexpression and improve the sensitivity of the assays. Briefly, YST cells maintained in DMEM supplemented with 1% penicillin/streptomycin and 10% fetal bovine serum were seeded at a density of 1 × 10^4^ cells per well in poly-L-lysine-coated eight-well chamber slides. After growing for 48 h at 37°C in a humidified incubator (5% CO_2_), the cells were transiently transfected with 50 ng each of plasmid expressing tyrosinase and plasmid expressing ATP7B variant using Lipofectamine LTX-PLUS transfection system. Cotransfection of two plasmids into YST cells was performed to model heterozygous state by using 25 ng each of the two selected plasmids.

### 2.3. Monitoring Protein Expression by GFP Fluorescence and Protein Activity by Tyrosinase Assay

Twenty-two hours after transfection, the expression of ATP7B-GFP variants was determined by detecting the GFP fluorescence signal using a Zeiss AXIO Vert A1 microscope at 40x. The cells were then washed with phosphate-buffered saline (PBS), fixed using ice-cold acetone–methanol (1:1 *v*/*v*) solution, and incubated with 0.4 mg/mL levodopa (3,4-dihydroxy-L-phenylalanine, Sigma-Aldrich) in 0.1 M sodium phosphate buffer (pH 6.8) for 2 h at 37°C. The slides were mounted using Fluoromount-G and imaged by phase contrast microscopy (Zeiss AXIO Observer Z1) at 20x magnification to detect the formation of the black eumelanin pigment. Experiments were performed in triplicate; at least 10 images were collected from each transfection for the analysis of GFP fluorescence and at least 20 images for the tyrosinase assay. The activity was detected by the appearance of dark pigment (eumelanin); the intensity of the pigment was quantified for selected variants using ImageJ software. The color intensity of the pigmented area was corrected for background noise, normalized by area, and compared to similarly quantified pigment intensity of WT ATP7B expressed in parallel. The data were plotted and analyzed in GraphPad Prism Version 10.2 using an unequal variances *t*-test.

### 2.4. Immunoblotting

YST cells were seeded in six-well (10 cm^2^) plates at a density of 1.6 × 10^6^ cells per well as described above. Four hundred nanograms of plasmid was used for transfection using the Lipofectamine LTX-PLUS transfection system. Twenty-two hours after transfection, the cells were collected and lysed using RIPA lysis buffer (0.05 M Tris–HCl, pH 7.0; 0.15 M NaCl; 0.25% deoxycholic acid; 1% NP-40; and 1 mM EDTA). Cell debris were removed by centrifugation at 3000 × g for 15 min. The supernatant was determined for protein concentration using the Pierce 660 nm assay (Thermo). Approximately 10 *μ*g of protein was run on an SDS-PAGE using a 10% Laemmli gel (BioRad, United States), followed by transfer to a PVDF membrane using the Transblot Turbo system (Bio-Rad, United States). The membranes were blocked with 5% milk in PBST for 1 h at room temperature (RT), followed by incubation with rabbit anti-ATP7B (1:5000; Abcam) and rabbit anti-*β*-actin (1:2000, Abcam) primary antibodies for 16 h at 4°C. The membranes were then incubated with goat anti-rabbit IgG-HRP (1:10000; Santa Cruz) secondary antibody for 1 h at RT. The membranes were treated with Supersignal West Pico PLUS (Thermo Fisher Scientific) substrate enhancer solution for 1 min at RT. The membranes were imaged using the Amersham Imager 600 system (GE healthcare).

### 2.5. Analysis of Databases and Structural Damage Prediction

NCBI ClinVar (http://ncbi.nlm.nih.gov/clinvar/) [40] was used to determine the clinical significance of the variants. ClinVar and UniProt (http://uniprot.org) [41] were both used to identify available functional information on the variants. Missense3D (http://missense3d.bc.ic.ac.uk/) was used to predict structural damage caused by S657R, G1061E, and G1266R substitutions and Missense3D-TM [42, 43] to assess the impact of the S657R variant. Variant effect prediction AlphaMissense dataset [44] for human ATP7B was downloaded from AlphaFold DB (https://alphafold.ebi.ac.uk/) [45]. Alignment-free estimation of sequence conservation of human ATP7B was determined by the algorithm developed by Yeung et al. [46] mapping conservation scores on AlphaFold 2 models. Human ATP7B (UniProt ID P35670) orthologs (H2Q7L5, Q64446, A0A6I8R0A5, A0A8N7T720, G5EE14, Q6H7M3, P38995, and Q5ZWR1) were aligned in Jalview 2.11.3.3 [47] using its alignment tool [48, 49] with the Clustal algorithm in default settings and Clustal X color scheme [50]. WebLogo 3 with hydrophobicity coloring [51] was created based on multiple sequence alignment by ColabFold notebook [52] workspace ConservFold setup with default parameters with the input sequence of human ATP7B residues 484–1465. Structure of Mg^2+^/ATP-bound ATP7B was generated using AlphaFold Server (https://golgi.sandbox.google.com/) employing the AlphaFold 3 prediction algorithm [53]. The best ranked AlphaFold 3 prediction was used for further analysis (ipTM = 0.89; pTM = 0.47). Root mean square deviation was calculated between aligned residues (1006–1104 and1147–1303) of the predicted ATP-bound ATP7B model and crystal structure of the CopA P- and N-domains bound with Mg^2+^/adenylyl methylenediphosphonate (PDB ID 3A1C) [54] using the ChimeraX MatchMaker tool [55, 56]. The three main variants of interest (S657R, G1061E, and G1266R) were introduced into the presented structural models using the ChimeraX *swapaa* function in the default settings, visualizing the least conflicting rotamer.

## 3. Results

### 3.1. Selecting WD-Causing ATP7B Variants for Screening

For this study, we selected 101 ATP7B mutants with various classifications of clinical significance to determine their effect on protein expression and activity. Based on the ClinVar database, out of the 101 variants, 31 are clinically classified as pathogenic or likely pathogenic, five as benign or likely benign, 59 have conflicting classifications of pathogenicity or are classified as variants of uncertain significance, and six are not yet in the database, as summarized in Table S1. Thirty-three mutants have been previously functionally characterized using in vitro methods, as summarized in Table S1.

**Figure 1:**
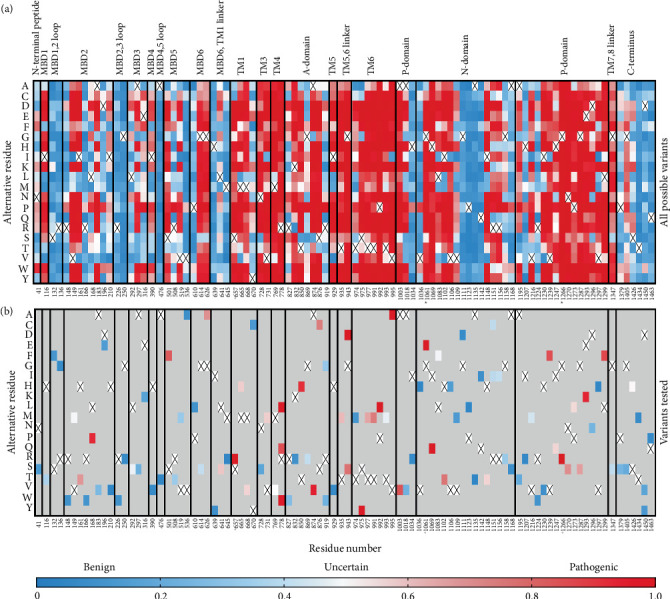
(a) AlphaMissense pathogenicity heat map of ATP7B showing all reported amino acid alterations at the given position and (b) specific missense variants experimentally tested in this work. Variants later identified to completely abolish ATP7B Cu transport activity (S657R, G1061E, and G1266R) are labeled in bold with the asterisk below each panel.

Since the ClinVar database contains uncertain classification for about two-thirds of the studied variants, we plotted a heat map visualizing AlphaMissense pathogenicity scores for all 101 variants spread across the entire ATP7B sequence to predict their pathogenic potential (Figure 1). AlphaMissense [44], a deep learning algorithm trained on variant population frequency data in the context of predicted AlphaFold 2 structures, provides reliable proteome-wide prediction of the impact of all possible amino acid substitutions [57]. This tool is especially useful for assessing uncharacterized missense variants. The pathogenicity score heat map for ATP7B illustrates that the regions negatively affected by amino acid alteration localize mainly in TM helices and catalytic domains, whereas the N- and C-termini seem less susceptible to deleterious alterations (Figure 1). Clinical data show that the pathogenic missense variant distribution correlates with the trend predicted by AlphaMissense [58–60].

### 3.2. Screening 101 ATP7B Variants in a Cellular Model of Cu Overload

**Figure 2:**
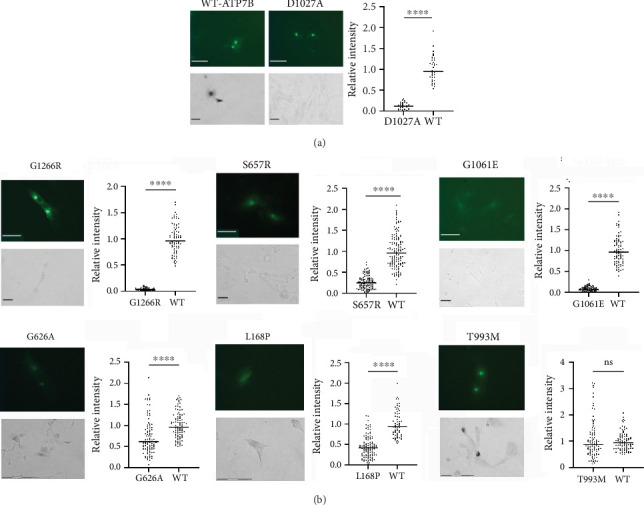
Expression and activity of representative ATP7B variants in YST cells. Top panels: The expression of GFP-tagged ATP7B visualized using GFP fluorescence (scale bar: 50 *μ*m). Bottom panels: The Cu transport activity of ATP7B was evaluated via the tyrosinase activation assay. The formation of black eumelanin pigment indicates active tyrosinase and therefore Cu transport by ATP7B (scale bar: 50 *μ*m). (a) WT ATP7B and D1027A are the positive and negative controls, respectively. (b) Representative mutants illustrating a broad range of protein levels and activities under identical experimental conditions. Complete Cu transport inactivation was observed for G1266R, G1061E, and S675R variants. ⁣^ns^*p* value > 0.05; ⁣^∗∗∗∗^*p* value < 0.0001.

To provide an initial functional characterization of selected ATP7B variants, we introduced site-specific mutations to an N-terminally GFP-tagged wild-type ATP7B construct. The identical amounts of plasmids encoding these constructs were transfected into human Menkes fibroblast cells (YST). These cells lack active Cu transporters ATP7A and ATP7B, accumulate Cu [38, 39], and lack Cu delivery to the Cu-dependent enzymes in the secretory pathway. The cells were imaged by fluorescence microscopy 22 h post-transfection to detect the GFP signal, indicating expression of ATP7B. We then determined the Cu transport activity of the variants using a tyrosinase activation assay. Tyrosinase is a Cu-dependent enzyme that catalyzes the oxidation of levodopa into a dark pigment eumelanin. In YST cells, tyrosinase is inactive. Expression of transport-competent ATP7B results in the delivery of Cu to tyrosinase and activation of the enzyme, which leads to the production of eumelanin pigment in the presence of levodopa. Wild-type ATP7B [37] was used as the positive control, and the previously characterized catalytically inactive mutant D1027A [21, 61] was used as the negative control (Figure 2).

GFP fluorescence was observed for all mutants, indicative of their expression. Although an identical amount of plasmid was used for transfections, the protein fluorescence intensity differed significantly, pointing to differences in the stability of ATP7B variants. (Examples are shown in Figure 2b; the entire dataset can be found in Figure S2.) Similarly, differences in Cu transport activities were detected. Unexpectedly, 97% of the screened mutants had a measurable Cu transport activity, which was evident from the appearance of pigment in the YST cells (Figure 2b and Figure S2). Only three variants, G1266R (P-domain), G1061E (N-domain), and S657R (TM1), resulted in the complete inactivation of ATP7B (Figure 2b). The data are summarized in Table 1.

**Figure 3:**
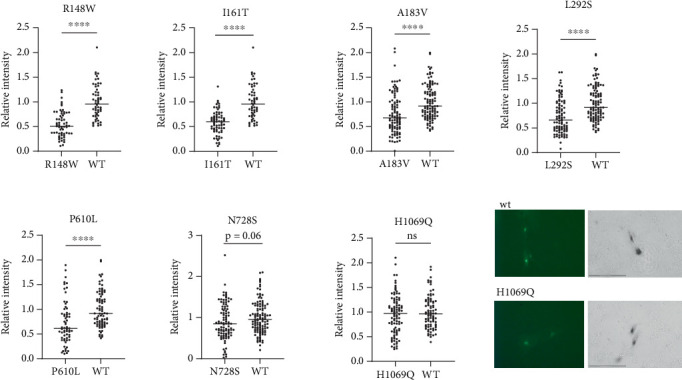
ATP7B variants that show a stronger reduction in Cu transport activity when compared to H1069Q mutant. Multiple images for WT ATP7B and each ATP7B variant were collected; pigment intensity was quantified in individual cells and plotted. H1069Q did not show a statistically significant difference in pigment intensity compared to WT ATP7B. ⁣^ns^*p* value > 0.05; ⁣^∗∗∗^*p* value < 0.001; ⁣^∗∗∗∗^*p* value < 0.0001.

Given the large number of mutants with residual activity, we first visually inspected the data to identify obvious changes in the expression and/or activity of ATP7B variants (Table 1). Out of the 29 variants classified as pathogenic or likely pathogenic in ClinVar, we saw reduced expression and/or activity in 80% of them, including G1266R, G1061E, and S657R, which we observed to be completely inactive. H1069Q and R778L, which are the most common WD-causing variants [15], both showed Cu transport activity (Figure 3, Table 1, and Figure S2).

The behavior of the H1069Q mutant highlighted the limitations of a tyrosinase activation assay. A previous analysis of the H1069Q mutant determined that it has lower catalytic and transport activity when compared to the wild-type ATP7B [21]. In the tyrosinase assay, this residual activity is sufficient to restore tyrosinase activity to levels similar to the wild-type (Figure 3). This is likely because the recombinant ATP7B variants are expressed at levels exceeding those of the endogenous protein, and even partial activity of the ATP7B variant can be sufficient for pigment appearance. Therefore, we reasoned that those mutants that do not restore pigment intensity to the wild-type levels are likely to be more deleterious than the H1069Q. Examples of such low activity mutants include previously uncharacterized variants R148W and L292S, the variants of unknown significance (I161T, A183VT, and P610L), and the variant with conflicting predictions (N728S) (Figure 3).

Aside from correlating functional defects to assigned clinical significance, screening of disease-causing variants also provides information about critical residues in the structure and function of ATP7B. Homology models of ATP7B using the LCopA structure have been generated [4, 5], and the structure of truncated ATP7B has become available [3]. Based on the generated model and assigned domain function, mutational variants were designated as “sensitive” if the amino acid variant is predicted to be disruptive to protein function. Sixteen of the mutants screened here were in sensitive positions based on this model [6]. Of these mutants, our results showed that five had reduced expression but significant activity (V1216M, T977M, T935M, P992L, and H1069Q), two had normal expression but reduced activity (T993M and N728S), one had reduced both expression and activity (L1299F), two had normal expression but no activity (G1266R and S657R), and one had reduced expression and no activity (G1061E). Five of these variants have been previously functionally characterized, whereas the rest have been screened for the first time in this work.

### 3.3. In Silico Analysis Rationalizes the Inactivating Effects of G1266R, S657R, and G1061E Mutations

**Figure 4:**
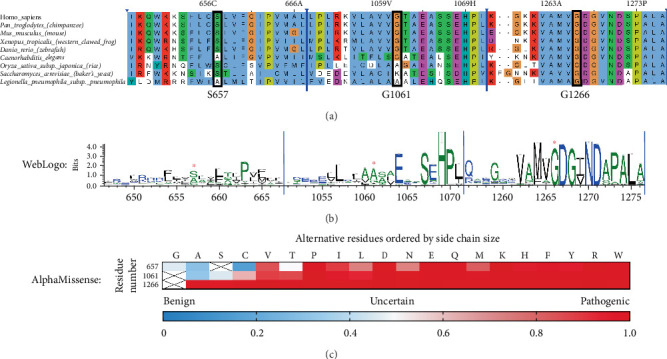
ATP7B sequence conservation in the regions where activity-abolishing variants S657R, G1061E, and G1266R occur. (a) Representative multiple sequence alignment (MSA) of ATP7B orthologs from model organisms and sequences for which experimental structures are available. The regions of primary sequence are centered around human ATP7B residues S657, G1061, and G1266. (b) WebLogo representation shown for the same sequence segments was generated from large scale MSA by ConservFold with human ATP7B as the input sequence and MMseqs2 as an algorithm to search cluster sequence data [52, 62]. (c) Variant effect prediction by AlphaMissense [57] for human ATP7B residues 657, 1061, and 1266 [57]. Crossed squares represent residues found in the wild-type sequence. ⁣^∗^ indicates the positions of residues 657, 1061, and 1266.

To better understand the severe impact of the three mutants that eliminate ATP7B Cu transport activity (S657R, G1061E, and G1266R), we examined the evolutionary conservation and potential consequences of these substitutions on the structure of ATP7B. Multiple sequence alignment of ATP7B orthologs from model organisms and of sequences for which experimental structures are available revealed the highest level of conservation for residue 1266, indicative of its essential role in ATP7B function (Figure 4a). Indeed, G1266 is located in the immediate vicinity of the catalytic site (see below).

Unlike G1266, the residues S657R and G1061E are not invariant; that is, they are important for ATP7B structure or functional dynamics but are not directly involved in Cu transport steps. WebLogo analysis (Figure 4b) supports this hypothesis. It shows that positions 657 and 1061 are frequently occupied by residues with small side chains. This points to potential spatial constraints for accommodating various residues. Indeed, as we show below, available ATP7B structures or structural models confirm that the residues in these positions are buried and face a rather hydrophobic environment. S657R and G1061E substitutions are bulky and introduce a charge. Additionally, the AlphaMissense algorithm-predicted pathogenicity scores for all possible substitutions in the discussed positions (Figure 4c) are in line with our predictions. While position 1266 may tolerate no substitutions, positions 657 and 1061 are to be less impacted when occupied by uncharged residues with small side chains. Taken together, our computational analysis; the experimental data showing complete loss of function; and previous identification of G1266R, S657R, and G1061E in WD patients [3, 63] indicate that these variants are pathogenic in the homozygous state (i.e., in the absence of other substitutions).

#### 3.3.1. The S657R Variant

**Figure 5:**
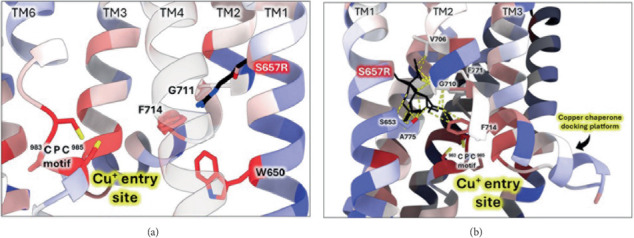
Structural model of in silico S657R substitution. Human ATP7B AlphaFold 2 model colored by estimated evolutionary conservation (red-to-blue from most conserved to least conserved). S657R variant was virtually introduced by ChimeraX. Mutated R657 is shown as sticks colored in black. (a) Rotamer with the best evaluation criteria protrudes towards Cu entry site. (b) Visualization of six R657 rotamers with the least number of clashes; clashes are shown in yellow dashed line.

To further investigate the S657R variant, we introduced a virtual mutation into the human ATP7B structural model using ChimeraX (Figure 5). S657 is located in the first transmembrane domain (TM1). When this serine is mutated, the arginine side chain best fitting rotamer invades into the Cu entry site, clashing with the conserved residue F714 and the TM2 hinge residue G710 (Figure 5a). Other potential rotamers make several clashes with the hinge region that bends TM2 into a functionally important Cu chaperone docking platform [64, 65]. Vulnerability of the TM2 hinge is highlighted by the fact that other WD variants occur in that sequence (G710S, G711R, and L708P) [66]. Moreover, S657R results in the “buried charge introduced” type of pathogenic impact when analyzed by Missense3D-TM [42]. Together, these effects might be severe enough to explain the inactivating effect of S657R.

#### 3.3.2. The G1061E Variant

**Figure 6:**
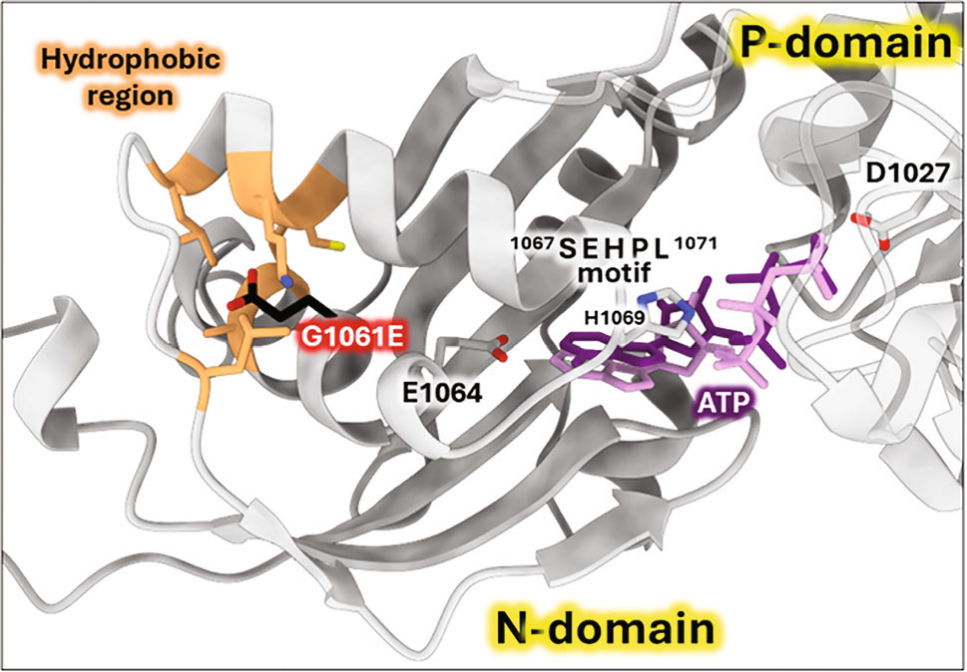
Structural model of in silico G1061E substitution. The AlphaFold 3 prediction of human wild-type ATP7B-Mg^2+^-ATP complex. ATP (dark purple) binding mode in the human ATP7B model closely fits the binding mode of adenylyl methylene-diphosphonate (AMP-PCP; light purple) in the experimental structure of the CopA PN-domain fragment [54] (1.163 and 2.483 Å RMSD between 185 pruned atom pairs and across all pairs, respectively). In silico mutated residue E1061 is shown as sticks colored in black G1061E virtual mutation was introduced into the model by ChimeraX, with the best-fitting rotamer shown.

The G1061E variant is located in the N-domain of ATP7B, which binds nucleotides (ATP/ADP) during the catalytic cycle. The structure of human ATP7B with a sufficiently high resolution for the N-domain to model ATP-binding is not available (the structure with PDB ID 8IOY has insufficient local resolution [66]). Consequently, we used AlphaFold3 to generate the structural model of the Mg^2+^-ATP-bound human wild-type ATP7B [53]. This model is in excellent agreement with the experimental NMR structure of the human ATP7B N-domain with bound ATP [67] and with the crystallographic structure of the PN-domain of CopA (bacterial ATP7B homolog) with the nonhydrolysable ATP analog AMP-PCP bound [54]. As shown in Figure 6, G1061 is located in the same *α*-helix as E1064, a residue directly involved in ATP-binding [54, 68]. Also, a highly conserved SEHPL motif involved in ATP coordination is present in the domain. Residue G1061 faces towards the inner region of the N-domain with a hydrophobic environment. Introducing bulky and charged glutamate side chain in this position may cause structural distortion, which would translate into impaired ATP coordination by the N-domain. Indeed, Missense3D analysis of G1061E variant in AlphaFold 3 model predicts structural damage with “buried charge introduced” and “buried glycine replaced” alerts [43].

#### 3.3.3. The G1266E Variant

**Figure 7:**
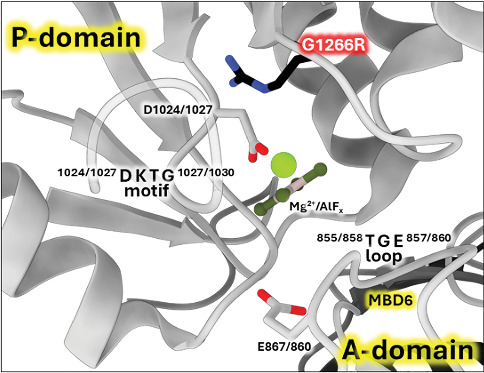
Structural model of the in silico G1266R substitution introduced into *Xenopus tropicalis* ATP7B structure (PDB ID 7SI3 [66]). Catalytically important motifs DKTG and TGE in the P- and A-domain, respectively, are labeled with *X. tropicalis* human sequence numbering. The structure mimics the so-called E2-P_i_ catalytic state by inhibiting the enzyme with aluminum fluoride complex (AlF_x_). The virtual R1266 mutation (human numbering) introduced in ChimeraX is shown as sticks colored in black.

The WebLogo diagram (Figure 4b) revealed G1266 as a highly conserved residue in the ATP7B sequence. The surrounding positions also have a high degree of conservation. As evident from the *Xenopus tropicalis* ATP7B structure [3], these residues lie opposingly to the invariant DKTG motif that contains catalytic aspartate and is involved in ATP hydrolysis (Figure 7) [69].

Such proximity to the protein catalytic center suggests that any substitutions of residue 1266 will probably have pathological consequences. This prediction agrees with our experimental data showing no activity for the G1266R mutant. The mutation of the same residue in paralogous ATP7A causes Menkes disease, the pathogenic effect most probably associated with the disruption of the phosphotransfer reaction that is common for both ATP7A and ATP7B [5].

#### 3.3.4. The G1272R Variant

Missense3D analysis of G1272R substitution in the experimental structure (*X. tropicalis* numbering; equivalent to human G1266R) predicts damaging impact with the “buried charge introduced” and “buried glycine replaced” alerts. We used *X. tropicalis* ATP7B for predictions because the 7SI3 model has the highest resolution (Figures S3A and S4B) and also shows high homology to human ATP7B (Figure S3B and S4A). In addition, the available human ATP7B experimental structures show the D1027A mutation (Figure 7) in the active site close to the position of the G1266R variant.

Twenty-nine of the tested variants are located in the N-terminal MBD region of the protein. For these variants, we used Missense 3D [43, 70] and available MBD structures to predict their effects on protein structure. Structural damage was predicted for G626A (MBD6), L168P (MBD2), and I161T (MBD2), all of which show significantly reduced Cu transport activity (Figures 2 and 3). Taken together, our results indicate that structural changes caused by the N-terminal domain variants negatively affect protein Cu transport function.

Four of the MBD variants were not predicted to affect structure but are located in conserved positions in the MBDs (Figure S5) likely serving an important role in the function of these domains. P610L (MBD6) is in a conserved proline position across the MBDs. Based on ClinVar, it is designated as a variant of uncertain significance, and there is no available experimental data for this protein. Our results show that this variant is associated with decreased ATP7B expression and activity in YST cells; therefore, the mutated proline must have a key role in protein folding, domain interactions, and function. L292S (MBD3) and A183V (MBD2) are both in conserved positions; these variants show reduced transport activity. L292S is not in ClinVar, while A183V is classified as a variant of uncertain significance. R148W (MBD2) variant replaces a conserved arginine and has reduced Cu transport activity. These results update ClinVar classifications.

### 3.4. Coexpression of Different ATP7B Variants Yields Intermediate Phenotype

**Figure 8:**
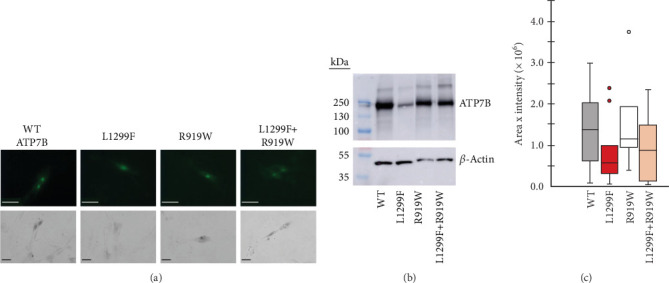
Coexpression of variants with different functional properties. (a) GFP fluorescence (scale bar: 50 *μ*m) and tyrosinase assay (scale bar: 50 *μ*m) of individual and coexpressed plasmids. (b) Immunoblotting of individual and coexpressed plasmids. (c) Quantification of tyrosinase assay signals for WT, R919W, L1299F, and R919W coexpressed with L1299F.

WD has a high prevalence of compound heterozygous variants. In cells, ATP7B can form dimers [71]. Therefore, different ATP7B mutants can interact and, as a result, have functional properties distinct from their individual properties examined in isolation [23]. To test this, we coexpressed equal amounts of two ATP7B mutants with different properties. R919W is a variant in the A-domain classified as a variant with conflicting evidence of pathogenicity in ClinVar and which we found to be normally expressed with activity similar to WT in the tyrosinase assay. On the other hand, L1299F is a variant in the P-domain classified in ClinVar as pathogenic or likely pathogenic. In our studies, we observed reduced expression and activity for this variant.  Figure 8 shows the expression (GFP fluorescence (Figure 8) and immunoblotting (Figure 8)) and transport activity (tyrosinase assay (Figure 8a,c)) for WT, L1299F, and R919W in isolation and following coexpression of R919W and L1299F. The coexpressed variant sample has lower GFP fluorescence and tyrosinase signal compared to R919W alone.

Immunoblotting and densitometry of the individual and coexpressed variants showed that the expression of R919W is 94% that of WT, while L1299F is 61%. Coexpressing R919W and L1299F resulted in expression that is 87% that of WT, in-between the values of R919W and L1299F alone (Figure 8). The activity of R919W is 113% of WT, based on pigment intensity. Coexpressing R919W and L1299F resulted in the activity that was 76% of WT, also in-between of the variant's individual values. Thus, in characterizing the disease-causing ATP7B variants, it is important to note whether the variants occur in homozygous or compound heterozygous states in the disease and model these zygosities in functional investigations. More detailed studies need to be done to account for the lower expression of one variant and how this affects its interaction with the other expressed variant.

## 4. Discussion

A very large number of missense mutations identified in the *ATP7B* gene have long been a challenge for studies of the functional consequences of these substitutions. Detailed analysis of the most common variants, such as H1069Q, has been done and yielded important information on its stability, activity, and regulation. Such a thorough analysis is not feasible for over 700 WD-associated variants and their combinations in the compound heterozygous states. Therefore, a functional screen serves as a useful tool for initial categorization of variants into completely inactive, partially active, and those with apparent normal activity that may have defects in regulation, trafficking, or mRNA splicing. With this in mind, we performed a screen of 101 ATP7B variants under identical conditions and characterized their ability to transport Cu. The assay yielded important initial data for previously uncharacterized mutants, facilitating further studies of these variants. Our data also highlight the limitations of such a screen.

When expressed and characterized as homozygous variants, only three mutants showed complete loss of Cu transport activity. In silico analysis of the three completely inactive variants (S657R, G1061E, and G1266R) [3, 63] suggests that in each case, the disruptive effect originates from introducing bulky and charged residue side chains in spatially constrained and functionally important protein regions. The surprisingly large number of Cu transport-competent ATP7B variants is likely a result of transient transfections of ATP7B cDNA, which yields protein abundance exceeding the levels of endogenous ATP7B. Although we optimized and carefully monitored protein expression to avoid significant overexpression, the amounts of produced mutants are still greater than the endogenous ATP7B levels. Therefore, low Cu transport activity of mutants can be amplified to levels sufficient to fully activate tyrosinase. Thus, the screen confidently identifies inactive variants and variants with significantly reduced activity (Figure 2 and Table 1), whereas those with “normal” activity need further study.

We did not investigate Cu-induced trafficking; therefore, ATP7B variants with normal Cu transport activity may have functional defects in these other properties of ATP7B. For example, we did not observe reduced expression or activity for R919G variant, and studies in CHO cells showed that it did not traffic in response to elevated Cu levels [72]. Studies with the recombinant cDNA also do not model mutation-induced changes in mRNA splicing, which could significantly decrease RNA/protein levels. This is the case for the M645R variant. Previous studies of recombinant cDNA bearing this mutation and our current studies found that the M645R substitution produces active ATP7B. However, studies of gene-edited HepG2 cells combined with the min-gene investigation revealed that a c.1934T>G substitution results in ~70% skipping of Exon 6, a frameshift generating the stop codon, and the loss of *ATP7B* function [24].

In vivo, many WD-associated variants occur as compound heterozygotes. The different ATP7B variants can dimerize, producing complexes with different properties compared to individual partners [23]. Our experiments on coexpressing a functionally defective ATP7B variant with a functionally normal variant resulted in ATP7B characteristics intermediate, but not average, between the two variants. Thus, further dedicated studies are needed to carefully model the compound heterozygous state to understand whether the activity and/or expression of ATP7B exist in these cases. The WD patient-derived iPS cells could be a suitable model for such studies. It would be interesting to determine whether gene and mRNA editing of one allele with a “severe” ATP7B mutation will be sufficient to rescue the phenotypes of cells carrying a combination of severe and mild ATP7B variants.

Although this and other complementation assays [73–75] offer only semiquantitative analysis of ATP7B mutants, they convincingly demonstrate that many ATP7B variants are not irreversibly impaired and, under certain conditions, can transport Cu and deliver Cu to Cu-dependent enzymes. This finding is significant, as it supports the earlier suggestions that small “corrector” molecules could be a potentially beneficial therapeutic approach to disease treatment. Such an approach has been employed for patients with cystic fibrosis [76] and should be considered for WD as one of several alternatives.

## 5. Conclusions

In this study, we screened 101 WD-associated variants and provided the first functional characterization for 70 ATP7B variants. The reduction in expression and/or activity was observed in 63% of the variants. The screen yielded additional information for variants that are classified as of uncertain significance in ClinVar: we observed functional defects in 12 out of the 21. The initial functional information for those variants that are not yet in the database revealed functional defects in six out of the 11 variants. Our results correlate well with pathogenic classification in ClinVar and previous reports of functional characterization. Taken together, the screen enabled identification of the most severely affected ATP7B variants and those with limited function.

## Figures and Tables

**Table 1:** Summary of expression (±) and activity (±) of screened ATP7B variants and their comparison to WT (=/↓). Variants are colored based on expression and activity compared to WT: in bold—complete protein inactivation; in italic—reduced expression and activity; and in bold-italic variants—normal expression and significant activity.

**Domain**	**Amino acid variant**	**Expression (±)**	**Expression compared to WT (=/↓)**	**Activity (±)**	**Activity compared to WT (=/↓)**
C-terminus	***Q1463P***	***+***	**=**	***+***	**=**
	*D1450V*	*+*	=	*+*	↓
	***D1450Y***	***+***	**=**	***+***	**=**
	***T1434M***	***+***	**=**	***+***	**=**
	*S1426I*	*+*	=	*+*	↓
	***G1405S***	***+***	**=**	***+***	**=**
	*P1379S*	*+*	↓	*+*	=

TM7,8 linker	*G1347S*	*+*	↓	*+*	=

P-domain	***L1299F***	***+***	**↓**	***+***	**=**
	***V1297I***	***+***	**=**	***+***	**=**
	*D1296N*	*+*	=	*+*	↓
	***E1293K***	***+***	**=**	***+***	**=**
	***G1287S***	***+***	**=**	***+***	**=**
	***P1273L***	***+***	**=**	***+***	**=**
	***N1270S***	***+***	**=**	***+***	**=**
	**G1266R**	**+**	**=**	**−**	**−**
	***H1247Q***	***+***	**=**	***+***	**=**
	*V1239F*	*+*	=	*+*	↓
	***I1230V***	***+***	**=**	***+***	**=**
	***R1224W***	***+***	**=**	***+***	**=**
	*V1216M*	*+*	↓	*+*	=
	***H1207R***	***+***	**=**	***+***	**=**
	*A1195T*	*+*	=	*+*	↓

N-domain	***A1168S***	***+***	**=**	***+***	**=**
	*G1158R*	*+*	↓	*+*	=
	***R1156H***	***+***	**=**	***+***	**=**
	*R1151H*	*+*	↓	*+*	↓
	*R1151C*	*+*	↓	*+*	=
	*I1148T*	*+*	↓	*+*	=
	***Q1142H***	***+***	**=**	***+***	**=**
	***A1135T***	***+***	**=**	***+***	**=**
	***P1123L***	***+***	**=**	***+***	**=**
	*G1111D*	*+*	↓	*+*	↓
	*V1109M*	*+*	↓	*+*	↓
	*V1106I*	*+*	↓	*+*	↓
	*I1102T*	*+*	↓	*+*	↓
	*L1083F*	*+*	↓	*+*	↓
	*H1069Q*	*+*	↓	*+*	=
	**G1061E**	**+**	**↓**	**−**	**−**
	***V1036I***	***+***	**=**	***+***	**=**

P-domain	*H1034R*	*+*	=	*+*	↓
	*A1018V*	*+*	↓	*+*	=
	*A1003V*	*+*	↓	*+*	↓

TM6	*V995A*	*+*	↓	*+*	=
	***T993M***	***+***	**=**	***+***	**=**
	*P992L*	*+*	↓	*+*	=
	*T991M*	*+*	↓	*+*	↓
	*T977M*	*+*	↓	*+*	↓
	*S975Y*	*+*	↓	*+*	=
	*T974M*	*+*	↓	*+*	=

TM5,6 linker	*G943D*	*+*	↓	*+*	=
	*G943S*	*+*	↓	*+*	=
	*T935M*	*+*	↓	*+*	=

TM5	*I929V*	*+*	=	*+*	↓

A	***R919W***	***+***	**=**	***+***	**=**
	***R919G***	***+***	**=**	***+***	**=**
	*S876C*	*+*	=	*+*	↓
	*A874V*	*+*	↓	*+*	↓
	*G869R*	*+*	=	*+*	↓
	***T850I***	***+***	**=**	***+***	**=**
	***K832R***	***+***	**=**	***+***	**=**
	***R827W***	***+***	**=**	***+***	**=**

TM4	*R778L*	*+*	↓	*+*	↓
	*R778Q*	*+*	↓	*+*	↓
	*R778W*	*+*	↓	*+*	↓
	*M769V*	*+*	=	*+*	↓

TM3	*V731M*	*+*	=	*+*	↓
	*N728S*	*+*	=	*+*	↓

TM1	*Y670C*	*+*	=	*+*	↓
	*M668V*	*+*	=	*+*	↓
	*M665I*	*+*	=	*+*	↓
	**S657R**	**+**	**=**	**−**	**−**

MBD6,TM1 linker	***M645R***	***+***	**=**	***+***	**=**
	***L641S***	***+***	**=**	***+***	**=**
	***H639Y***	***+***	**=**	***+***	**=**

MBD6	*G626A*	*+*	↓	*+*	↓
	*G614S*	*+*	=	*+*	↓
	*P610L*	*+*	↓	*+*	↓

MBD5	*V536A*	*+*	=	*+*	↓
	*V519M*	*+*	=	*+*	↓
	*R508T*	*+*	↓	*+*	↓
	*S501F*	*+*	=	*+*	↓

MBD4,5 loop	***A476T***	***+***	**=**	***+***	**=**

MBD4	*I390V*	*+*	=	*+*	↓

MBD3	***E316K***	***+***	**=**	***+***	**=**
	***A297S***	***+***	**=**	***+***	**=**
	*L292S*	*+*	=	*+*	↓

MBD2,3 loop	***G250R***	***+***	**=**	***+***	**=**
	*R226W*	*+*	↓	*+*	↓

MBD2	*I210V*	*+*	↓	*+*	↓
	***D196E***	***+***	**=**	***+***	**=**
	*A183V*	*+*	=	*+*	↓
	*L168P*	*+*	↓	*+*	↓
	***R166W***	***+***	**=**	***+***	**=**
	*I161T*	*+*	↓	*+*	↓
	*V149M*	*+*	=	*+*	↓
	*R148W*	*+*	=	*+*	↓

MBD1,2 loop	***R136G***	***+***	**=**	***+***	**=**
	*S132F*	*+*	↓	*+*	↓

MBD1	***I116T***	***+***	**=**	***+***	**=**

N-terminus	***N41S***	***+***	**=**	***+***	**=**

## Data Availability

Details regarding data availability and instructions for requesting information are available in the Alexion Clinical Trials Disclosure and Transparency Policy at https://alexion.com/our-research/research-and-development.

## Supporting Information

Supporting Information Additional supporting information can be found online in the Supporting Information section. Table S1: List of selected variants, their pathogenicity classification in ClinVar, and functional characterization. Figure S1: Plasmid expressing the N-terminally GFP-tagged wild-type human ATP7B [32]. Figure S2: Original data showing expression and copper-transport activity of all variants tested in this study. Figure S3: Available structural information for ATP7B. Figure S4: Amino acid sequences of human (PDB ID 7XUN) and frog (PDB ID 7SI3) ATP7B aligned in ChimeraX v1.8 using Clustal Omega v1.1.0 algorithm. Figure S5: Sequence alignment of ATP7B MBDs1-6.
