# Supplementary material for: Functional Screen of Wilson Disease ATP7B Variants Reveals Residual Transport Activities
Source: Hum Mutat. 2025 Jul 7;2025:7485658. doi: 10.1155/humu/7485658 (PMC12259332; doi:10.1155/humu/7485658)
Supplement: Supporting Information — Additional supporting information can be found online in the Supporting Information section. Table S1: List of selected variants, their pathogenicity classification in ClinVar, and functional characterization. Figure S1: Plasmid expressing the N-terminally GFP-tagged wild-type human ATP7B [32]. Figure S2: Original data showing expression and copper-transport activity of all variants tested in this study. Figure S3: Available structural information for ATP7B. Figure S4: Amino acid sequences of human (PDB ID 7XUN) and frog (PDB ID 7SI3) ATP7B aligned in ChimeraX v1.8 using Clustal Omega v1.1.0 algorithm. Figure S5: Sequence alignment of ATP7B MBDs1-6. [file 7485658.f1.pdf]

# Functional Screen of Wilson Disease ATP7B Variants Reveals Residual Transport Activities

Jenifer S. Calvo[1], Tomáš Heger[2], Ekaterina Kabin[1], William Mowrey[3], Guillermo Del Angel[3], Wei Ding[3⊥], Svetlana Lutsenko[1⊥]

## Supplementary Materials

**Table S1.** List of selected variants, their pathogenicity classification in ClinVar, and functional characterization. Rows are colored based on ClinVar classification: in red are pathogenic or likely pathogenic, in green are benign or likely benign, in gray are unknown significance or conflicting interpretations of pathogenicity, and non-colored variants are not in ClinVar.

| Functional Domain | Amino Acid variant | Clinical significance in ClinVar | Functional characterization in literature | Source |
|---|---|---|---|---|
| C-terminus | Q1463P | Not in ClinVar | Not available | N/A |
| | D1450V | Not in ClinVar | Not available | N/A |
| | D1450Y | Not in ClinVar | Not available | N/A |
| | T1434M | Conflicting interpretations of pathogenicity | Normal TGN localization | (14) |
| | S1426I | Not in ClinVar | Reduced Cu transport | (18) |
| | G1405S | Conflicting interpretations of pathogenicity | Not available | N/A |
| | P1379S | Conflicting interpretations of pathogenicity | Normal TGN localization | (14) |
| TM 7,8 linker | G1347S | Conflicting interpretations of pathogenicity | Not available | N/A |
| P-domain | L1299F | Pathogenic/Likely pathogenic | Not available | N/A |
| | V1297I | Conflicting interpretations of pathogenicity | Not available | N/A |
| | D1296N | Conflicting interpretations of pathogenicity | Not available | N/A |
| | E1293K | Conflicting interpretations of pathogenicity | Not available | N/A |
| | G1287S | Conflicting interpretations of pathogenicity | Mild to intermediate deficit in rescuing ccc2 activity in yeast | (69) |
| | P1273L | Pathogenic | Decreased Cu transport activity, increased ATPase activity | (16) |
| | N1270S | Pathogenic | Loss of Cu transport activity, increased ATPase activity | (16) |
| | G1266R | Pathogenic/Likely pathogenic | Low Cu transport, inactive phosphorylation | (16) |
| | H1247Q | Conflicting interpretations of pathogenicity | Not available | N/A |
| | V1239F | Not in ClinVar | Unable to rescue ccc2 activity in yeast | (69) |
| | I1230V | Conflicting interpretations of pathogenicity | Not available | N/A |
| | R1224W | Not in ClinVar | Not available | N/A |

| | | | | |
|---|---|---|---|---|
| | V1216M | Pathogenic/Likely pathogenic | Not available | N/A |
| | H1207R | Benign/Likely benign | Not available | N/A |
| | A1195T | Conflicting interpretations of pathogenicity | Not available | N/A |
| N-domain | A1168S | Uncertain significance | Not available | N/A |
| | G1158R | Uncertain significance | Not available | N/A |
| | R1156H | Uncertain significance | Not available | N/A |
| | R1151H | Conflicting interpretations of pathogenicity | Mild reduction in affinity for ATP | (64) |
| | R1151C | Pathogenic/Likely pathogenic | Not available | N/A |
| | I1148T | Pathogenic/Likely pathogenic | Mildly to intermediate deficit in rescuing ccc2 activity in yeast | (69) |
| | Q1142H | Conflicting interpretations of pathogenicity | Not available | N/A |
| | A1135T | Conflicting interpretations of pathogenicity | Not available | N/A |
| | P1123L | Uncertain significance | Not available | N/A |
| | G1111D | Uncertain significance | Not available | N/A |
| | V1109M | Not in ClinVar | Not available | N/A |
| | V1106I | Conflicting interpretations of pathogenicity | Not available | N/A |
| | I1102T | Pathogenic | Intermediate to severe deficit in rescuing ccc2 activity in yeast | (69) |
| | L1083F | Pathogenic/Likely pathogenic | Decreased copper transport activity; no effect on ATPase activity; decreased localization to the TGN | (16, 73) |
| | H1069Q | Pathogenic | Decreased stability; decreased copper transport activity; impaired ATPase activity | (16, 74) |
| | G1061E | Pathogenic | Reduced protein expression, retention in ER, no Cu transport | (18) |
| | V1036I | Uncertain significance | Mild effect on ccc2 activity in yeast. | (70) |
| P-domain | H1034R | Benign; likely benign | Not available | N/A |
| | A1018V | Pathogenic/Likely pathogenic | Not available | N/A |
| | A1003V | Pathogenic/Likely pathogenic | Not available | N/A |
| TM6 | V995A | Conflicting interpretations of pathogenicity | Fully complemented ccc2 activity in yeast. | (71) |
| | T993M | Conflicting interpretations of pathogenicity | Not available | N/A |
| | P992L | Pathogenic/Likely pathogenic | Partial Cu transport activity; normal phosphorylation; severely impaired ccc2 activity at 30°C, completely impaired at 37°C | (16, 71) |
| | T991M | Conflicting interpretations of pathogenicity | Mild to intermediate deficit in rescuing Ccc2p activity in yeast | (69) |

| | | | | |
|---|---|---|---|---|
| | T977M | Pathogenic | Unable to complement ccc2 activity in yeast | (71) |
| | S975Y | Pathogenic/Likely pathogenic | Not available | N/A |
| | T974M | Uncertain significance | Not available | N/A |
| TM5,6 linker | G943D | Pathogenic/Likely pathogenic | Not available | N/A |
| | G943S | Pathogenic | Slightly impaired complementation of ccc2 activity in yeast; normally localized to TGN but does not respond to Cu | (71, 75) |
| | T935M | Pathogenic/Likely pathogenic | Not available | N/A |
| TM5 | I929V | Conflicting interpretations of pathogenicity | Not available | N/A |
| A-domain | R919W | Conflicting interpretations of pathogenicity | Not available | N/A |
| | R919G | Pathogenic | Not available | N/A |
| | S876C | Not in ClinVar | Not available | N/A |
| | A874V | Conflicting interpretations of pathogenicity | Partial Cu transport, increased phosphorylation; No tyrosinase activity; decreased localization to the TGN | (6, 16) |
| | G869R | Pathogenic | Not available | N/A |
| | T850I | Pathogenic/Likely pathogenic | Not available | N/A |
| | K832R | Likely benign | Decrease Cu transport activity; no effect on ATPase activity | (16) |
| | R827W | Uncertain significance | Not available | N/A |
| TM4 | R778L | Pathogenic | Decreased Cu transport activity; localized to the ER | (75) (16) |
| | R778Q | Pathogenic | Decreased Cu transport | (71) |
| | R778W | Pathogenic | Not available | N/A |
| | M769V | Pathogenic | Partial Cu transport; increased phosphorylation; rescued ccc2 activity in yeast | (16)(71) |
| TM3 | V731M | Uncertain significance | Not available | N/A |
| | N728S | Conflicting interpretations of pathogenicity | Not available | N/A |
| TM1 | Y670C | Uncertain significance | Not available | N/A |
| | M668V | Conflicting interpretations of pathogenicity | Not available | N/A |
| | M665I | Conflicting interpretations of pathogenicity | Not available | N/A |
| | S657R | Conflicting interpretations of pathogenicity | Not available | N/A |
| MBD6, TM1 linker | M645R | Pathogenic | Recombinant ATP7B has normal abundance, Cu transport, and localization. Endogenously, altered mRNA splicing causes marked decrease in protein abundance and activity | (16) (76) (19) |

| | | | | |
|---|---|---|---|---|
| | L641S | Conflicting interpretations of pathogenicity | No effect on protein abundance, Cu transport, and localization | (76) (77) |
| | H639Y | Uncertain significance | No effect on protein abundance, Cu transport, and localization | (77) |
| MBD6 | G626A | Pathogenic/Likely pathogenic | No effect on protein abundance and protein localization; partial Cu transport; normal phosphorylation | (16) (73, 76) (77) |
| | G614S | Uncertain significance | Not available | N/A |
| | P610L | Uncertain significance | Not available | N/A |
| MBD5 | V536A | Conflicting interpretations of pathogenicity | Not available | N/A |
| | V519M | Uncertain significance | Not available | N/A |
| | R508T | Uncertain significance | Not available | N/A |
| | S501F | Uncertain significance | Not available | N/A |
| MBD4,5 linker | A476T | Conflicting interpretations of pathogenicity | Not available | N/A |
| MBD4 | I390V | Conflicting interpretations of pathogenicity | Not available | N/A |
| MBD3 | E316K | Uncertain significance | Not available | N/A |
| | A297S | Not in ClinVar | Not available | N/A |
| | L292S | Not in ClinVar | Not available | N/A |
| MBD2,3 linker | G250R | Uncertain significance | Not available | N/A |
| | R226W | Uncertain significance | Not available | N/A |
| MBD2 | I210V | Conflicting interpretations of pathogenicity | Not available | N/A |
| | D196E | Conflicting interpretations of pathogenicity | Not available | N/A |
| | A183V | Uncertain significance | Not available | N/A |
| | L168P | Conflicting interpretations of pathogenicity | Not available | N/A |
| | R166W | Conflicting interpretations of pathogenicity | Not available | N/A |
| | I161T | Uncertain significance | Not available | N/A |
| | V149M | Conflicting interpretations of pathogenicity | Not available | N/A |
| | R148W | Likely benign | Not available | N/A |
| MBD1,2 linker | R136G | Conflicting interpretations of pathogenicity | Not available | N/A |
| | S132F | Not in ClinVar | Not available | N/A |
| | I116T | Conflicting interpretations of pathogenicity | Not available | N/A |
| N-terminus | N41S | Pathogenic/Likely pathogenic | Affects Cu-induced relocalization | (12) |

**Supplementary Figures**

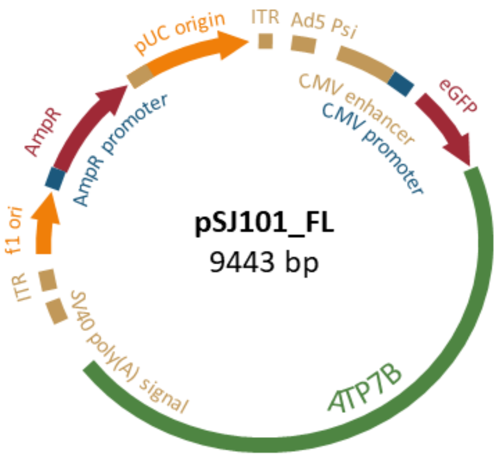


**Suppl Fig1.** Plasmid expressing the N-terminally GFP-tagged wild-type human ATP7B (32).

**Supplemental Figure 2** Original data showing expression and copper-transport activity of the variants tested in this study. WT control for each measurement is included on the left. The set of panels on the right show triplicate data on expression (GFP, green) and tyrosinase activity (dark pigment) for each listed mutant. Text in green color indicate activity and expression similar to WT, text in red indicate reduced expression and/or activity

# Q1463P (C-terminus)

**Normal expression; significant activity**

wt

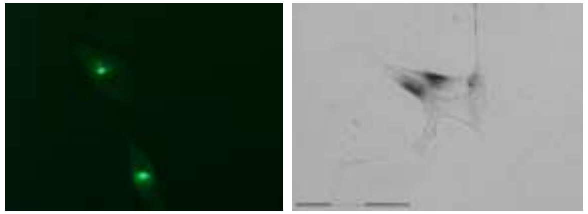

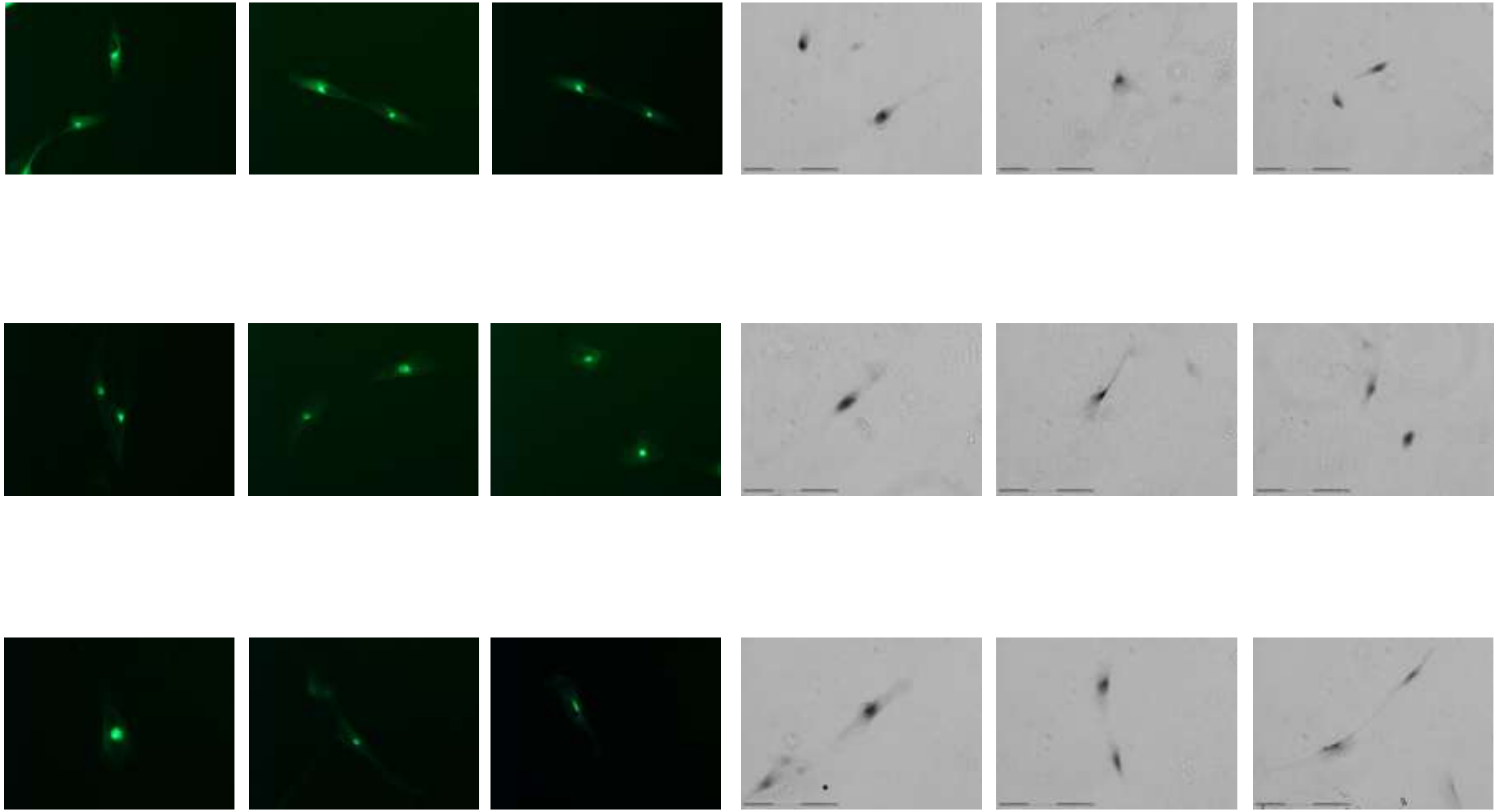

# D1450V (C-terminus)

Normal expression; reduced activity

wt

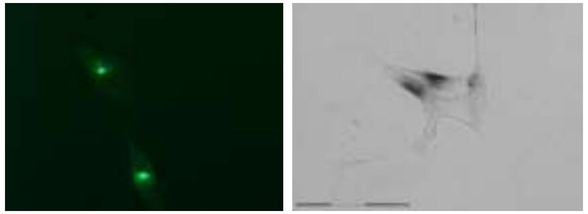

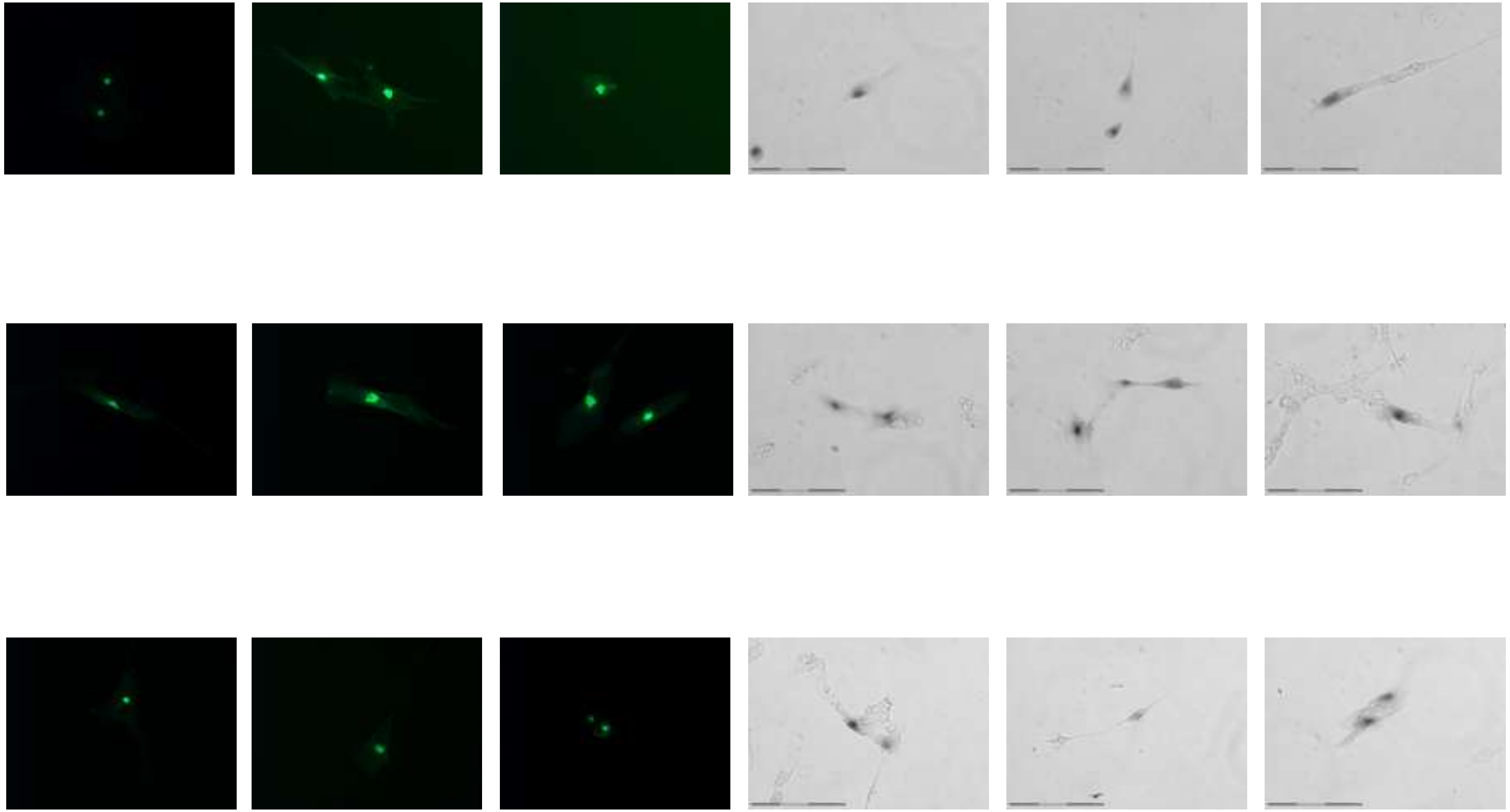

# D1450Y (C-terminus)

Normal expression; significant activity

wt

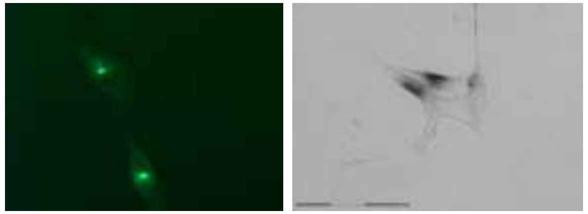

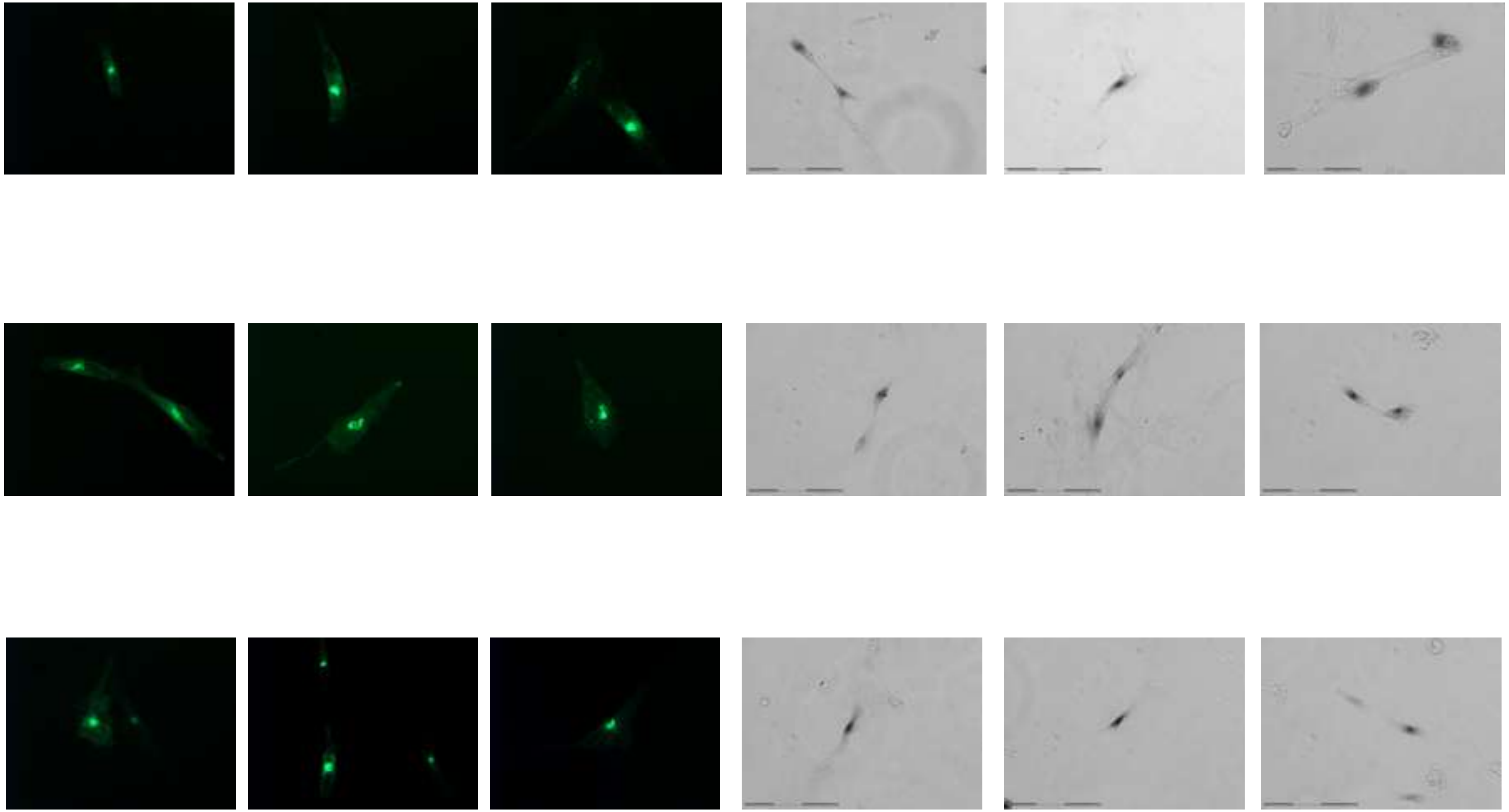

# T1434M (C-terminus)

**Normal expression; significant activity**

**wt**

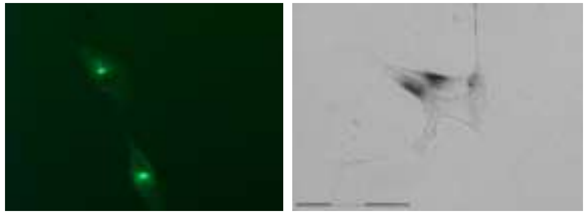

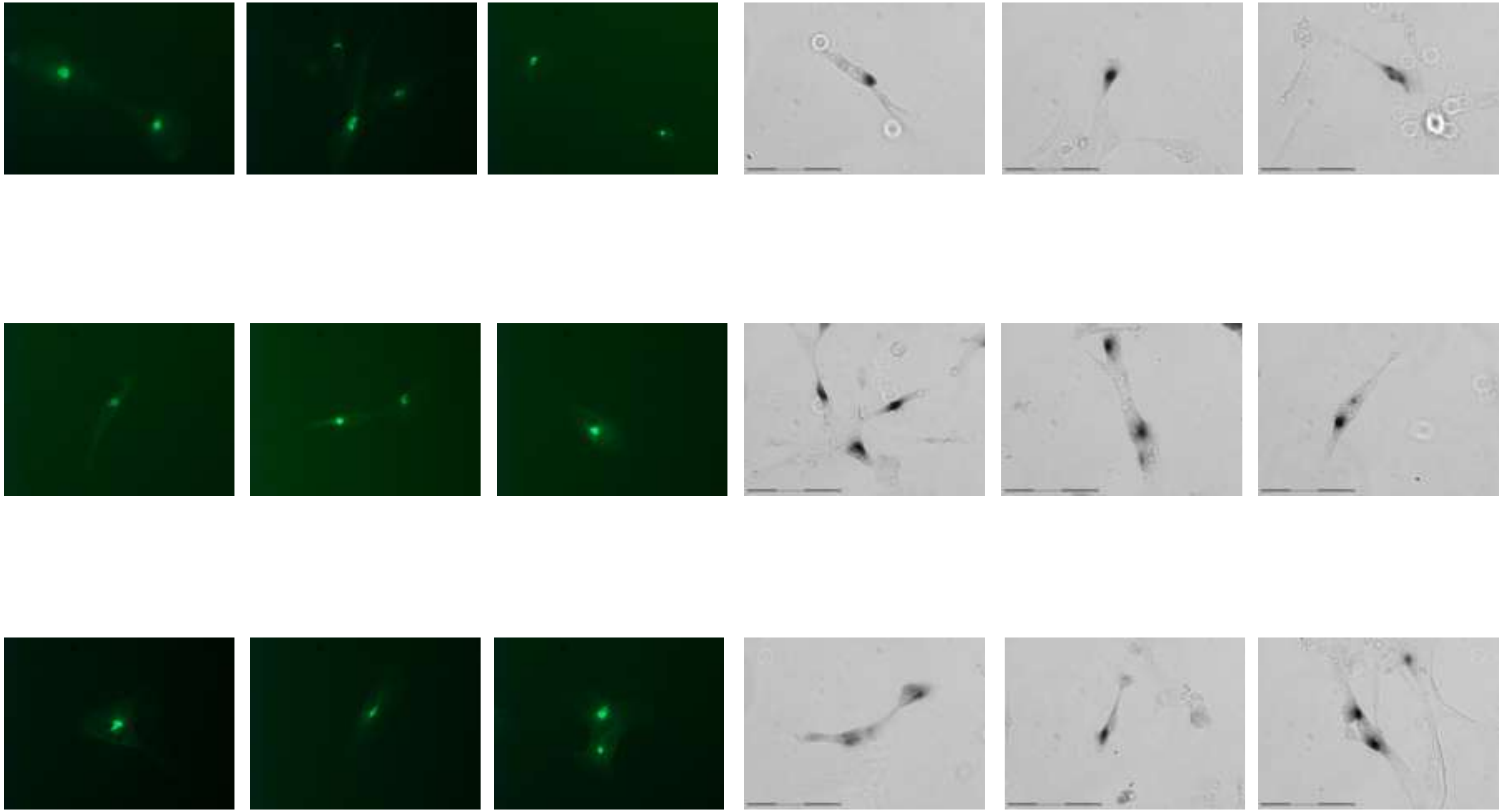

# 13: S1426I (C-terminus)

Normal expression; significant activity

wt

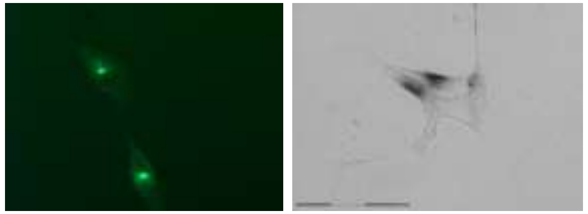

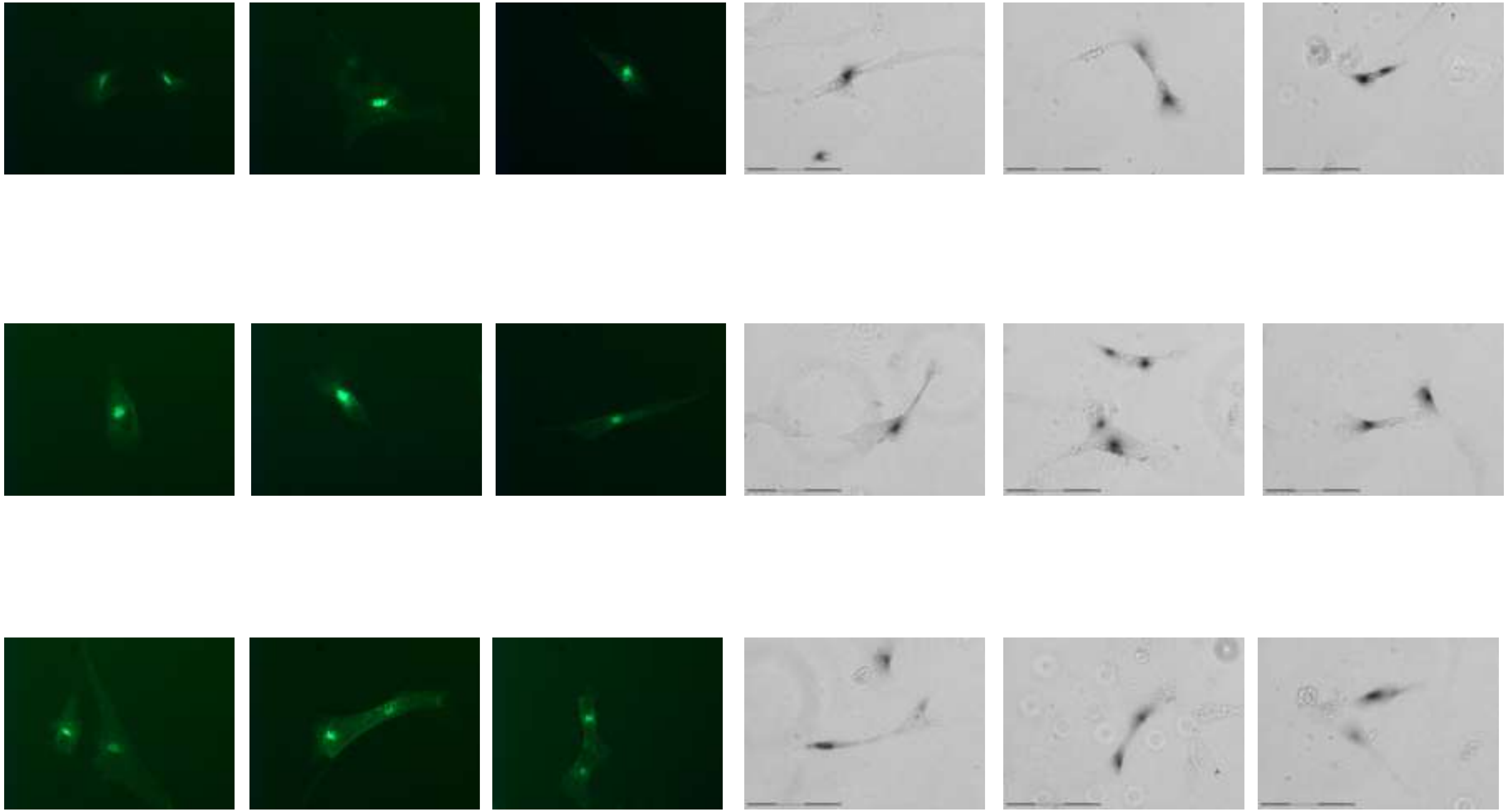

# G1405S (C-terminus)

**Normal expression; significant activity**

**wt**

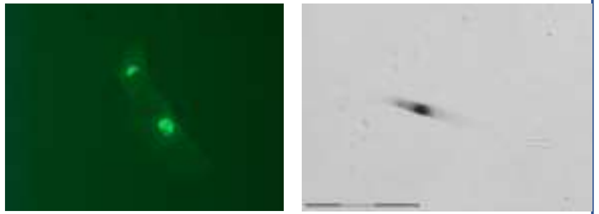

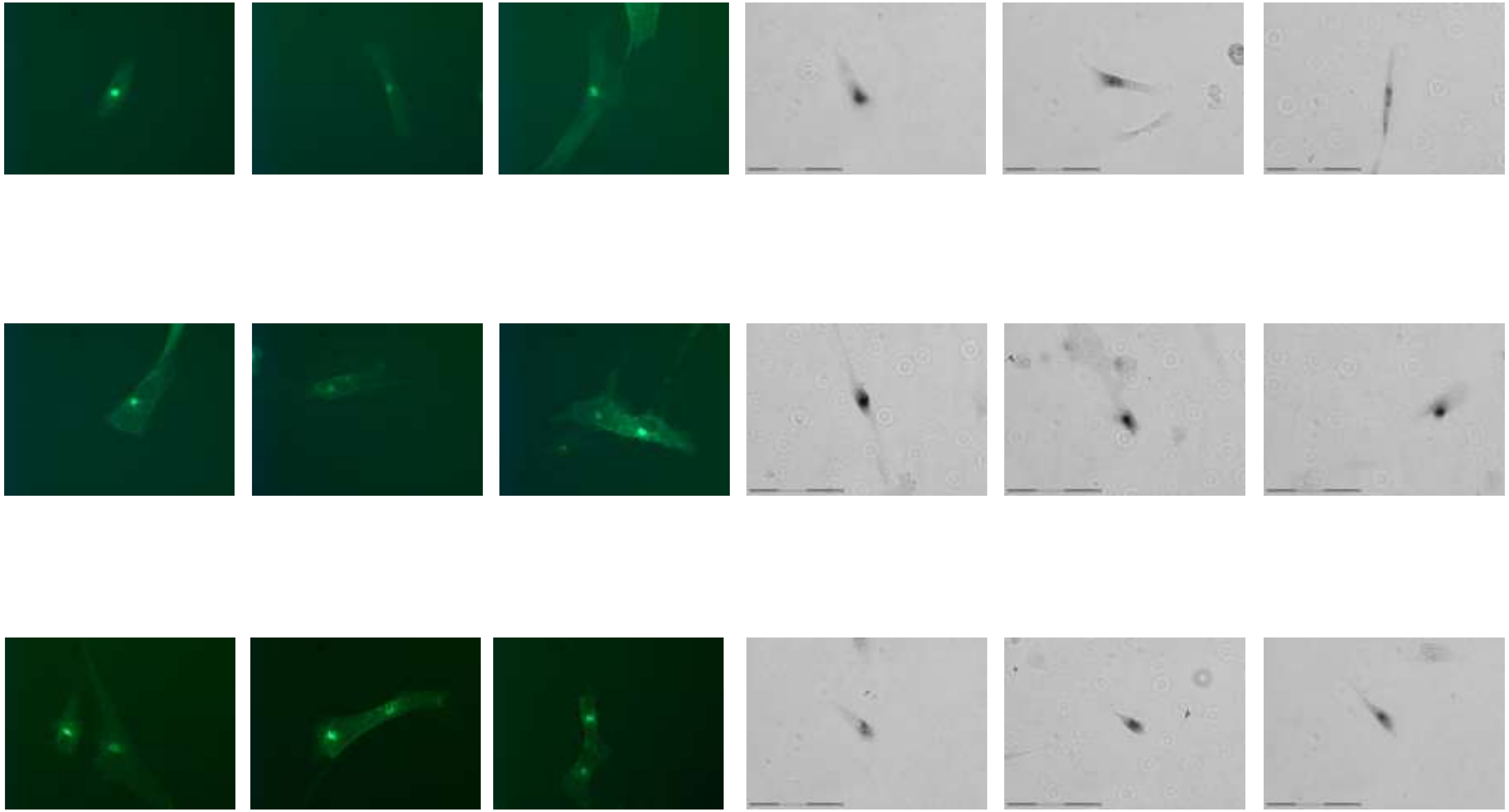

# P1379S (C-terminus)

Reduced expression; significant activity

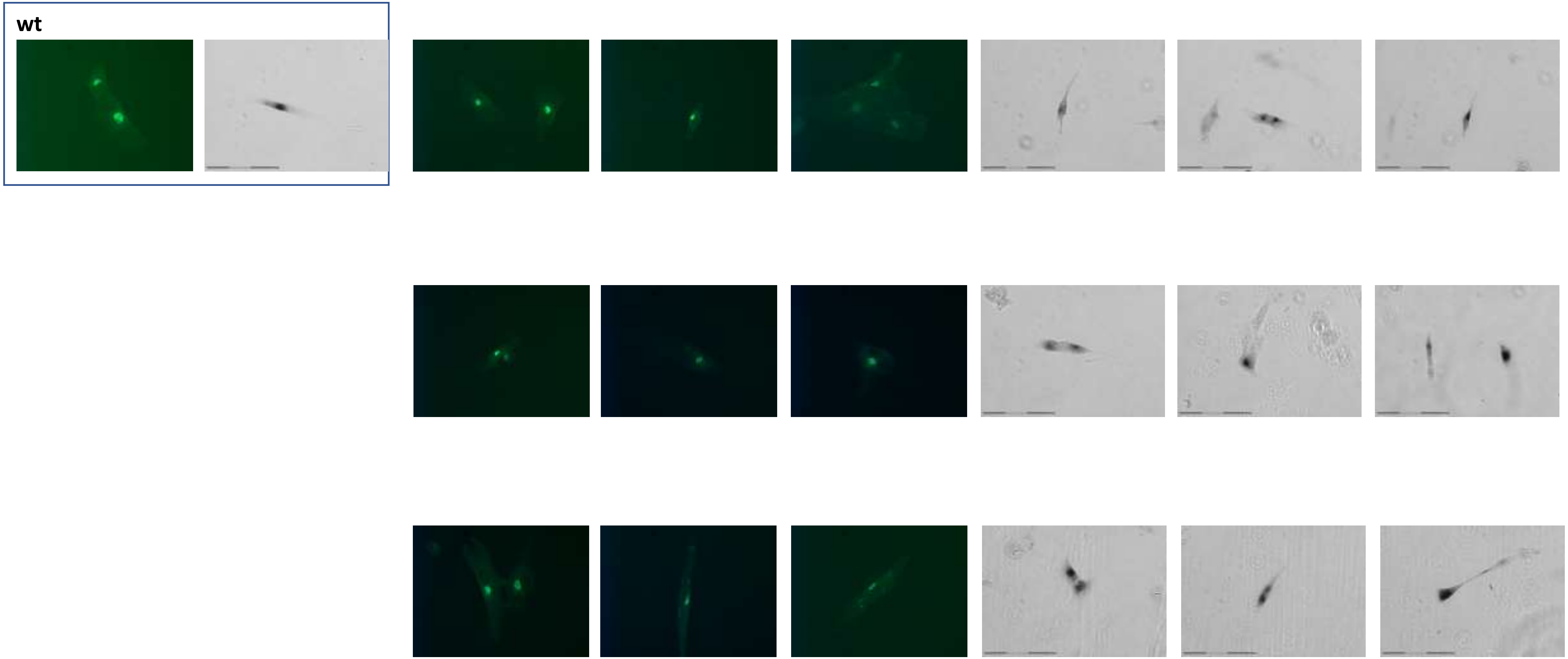

# G1347S (Extracellular between TM7 and TM8)

Reduced expression; significant activity

wt

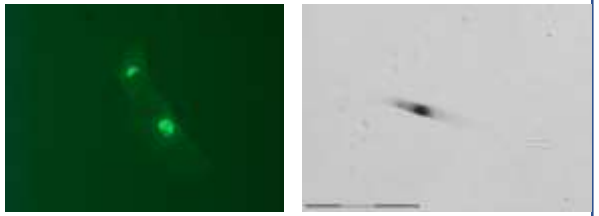

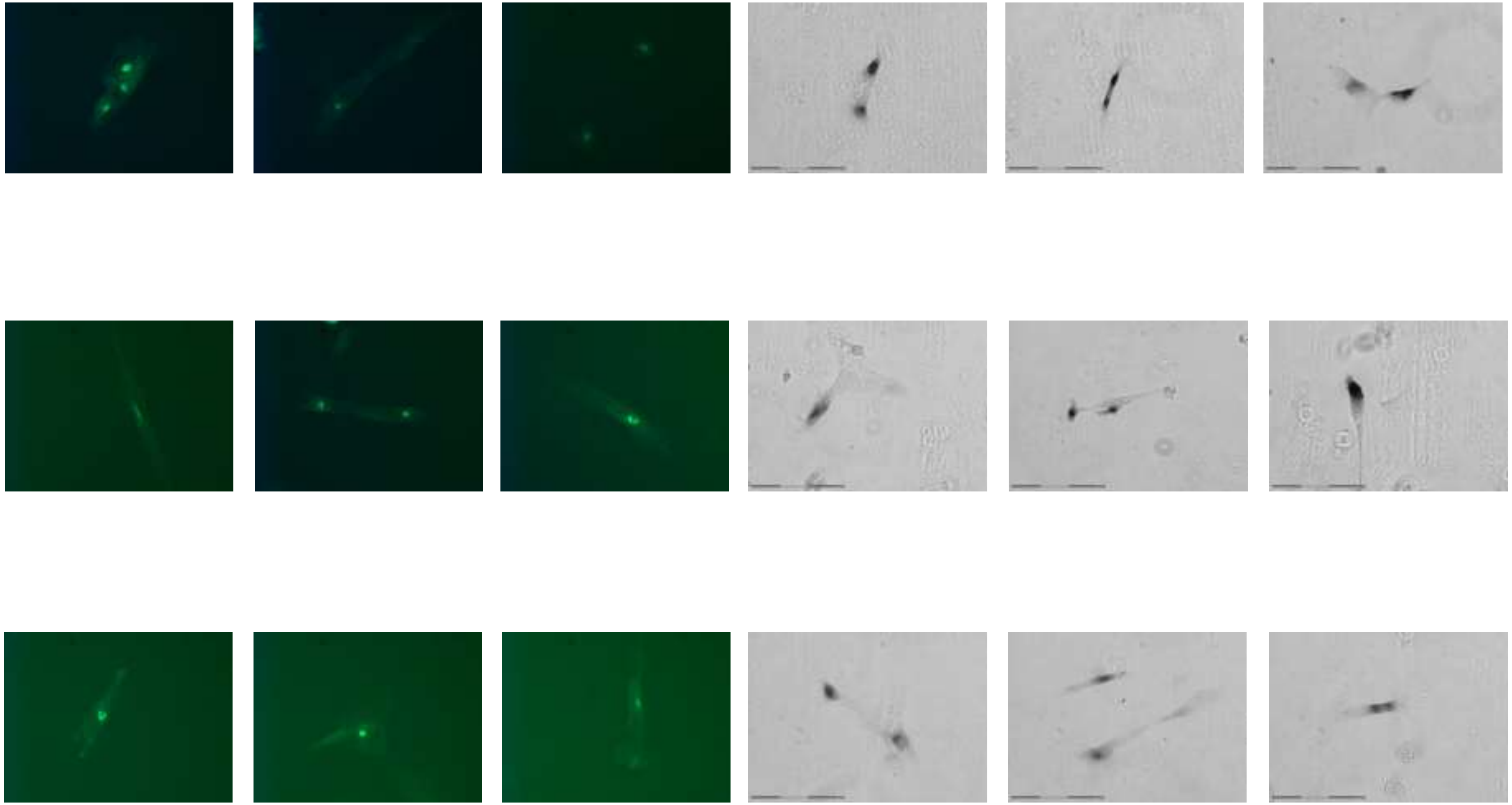

# L1299F (P-domain)

**Reduced expression; significant activity**

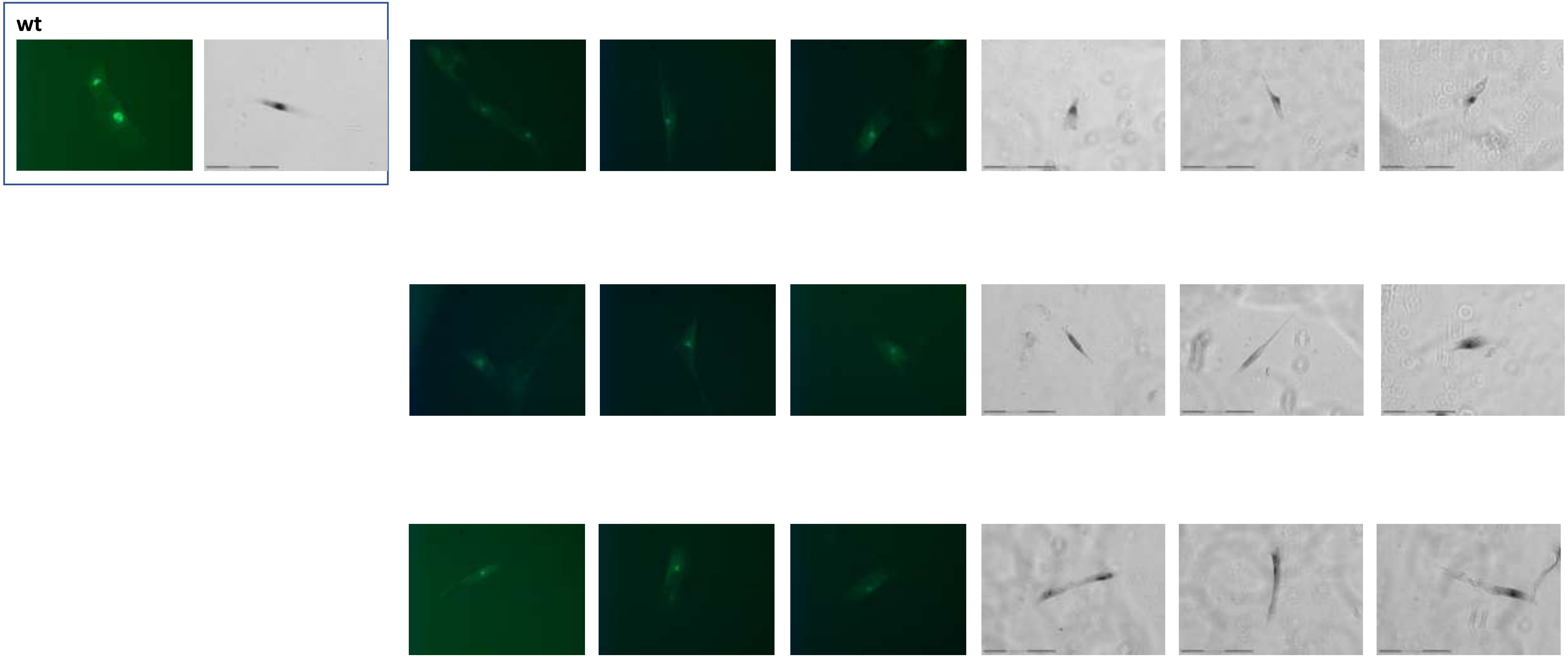

# V1297I (P-domain)

Normal expression; significant activity

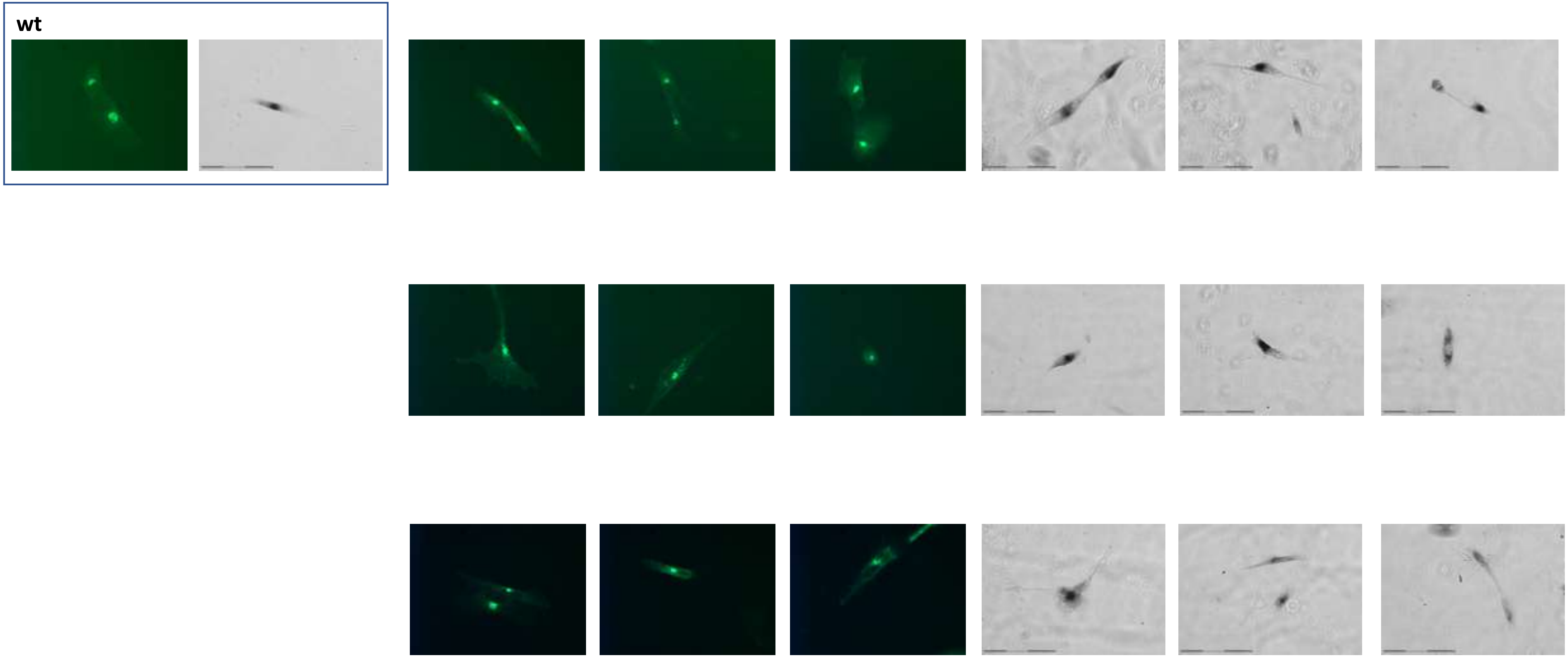

# D1296N (P-domain)

**Normal expression; reduced activity**

**wt**

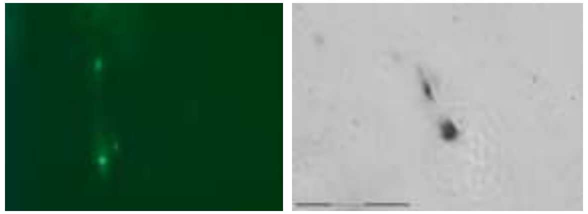

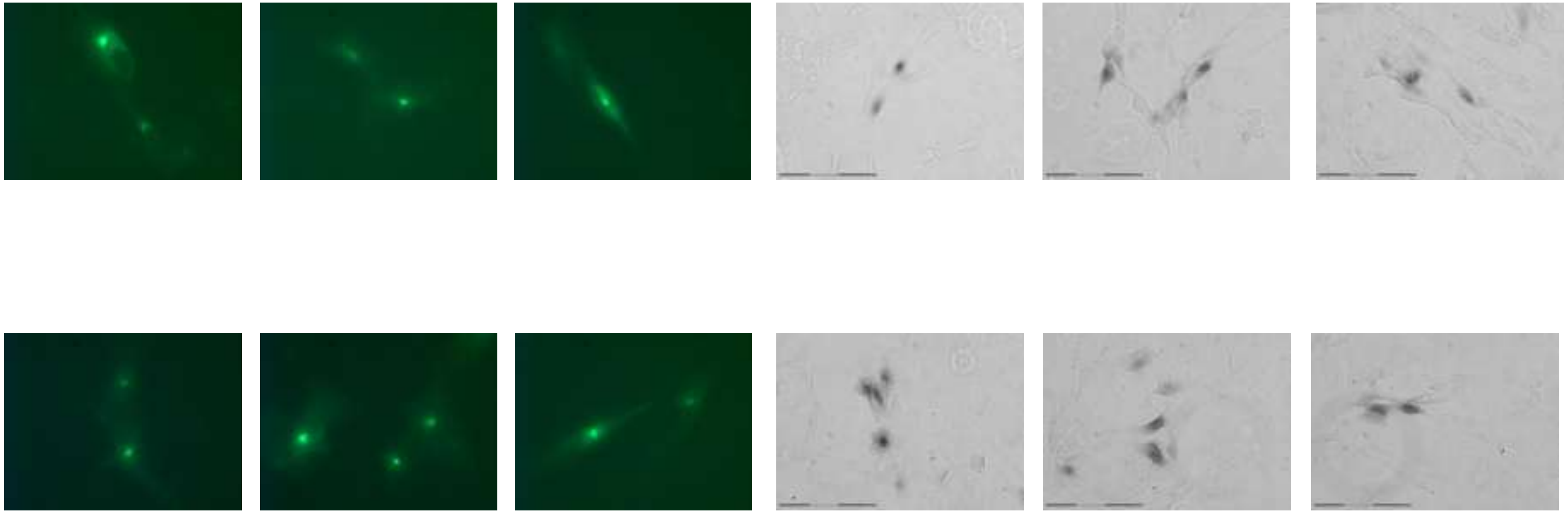

# E1293K (P-domain)

Normal expression; significant activity

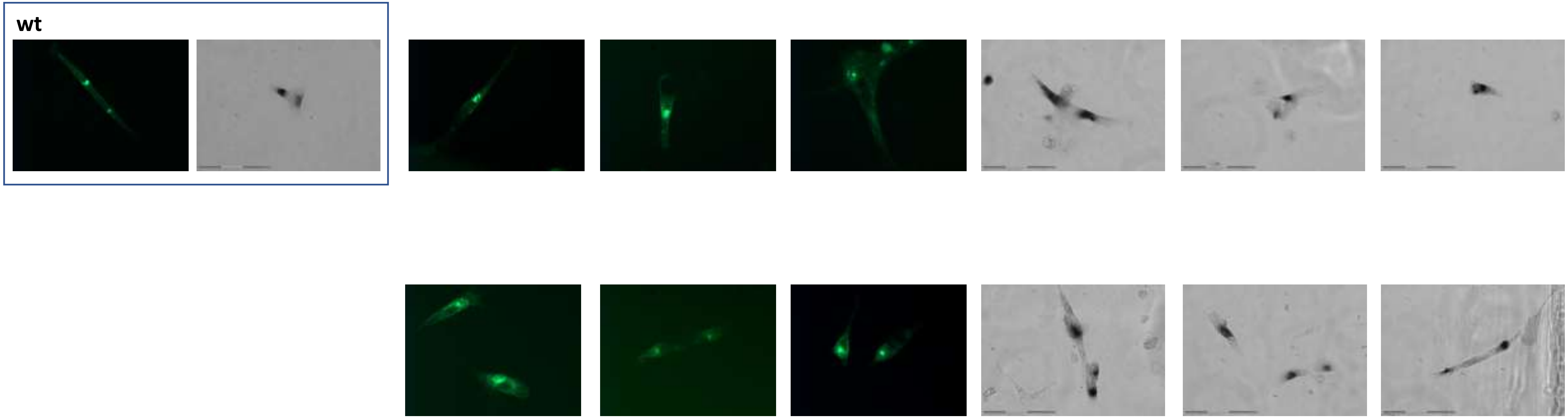

# G1287S (P-domain)

Normal expression; significant activity

wt

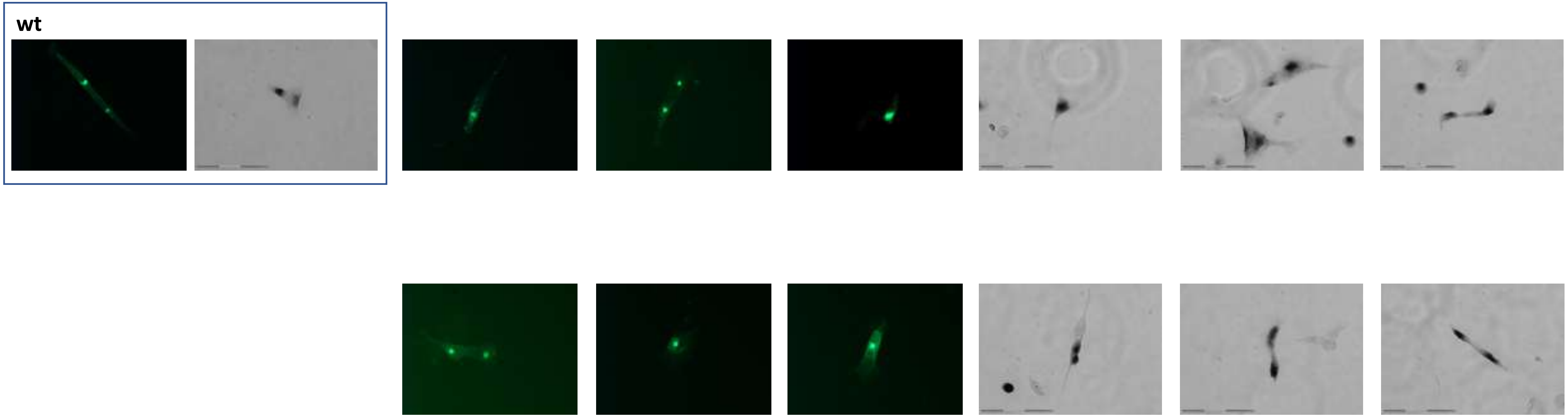

# P1273L (P-domain)

**Normal expression; significant activity**

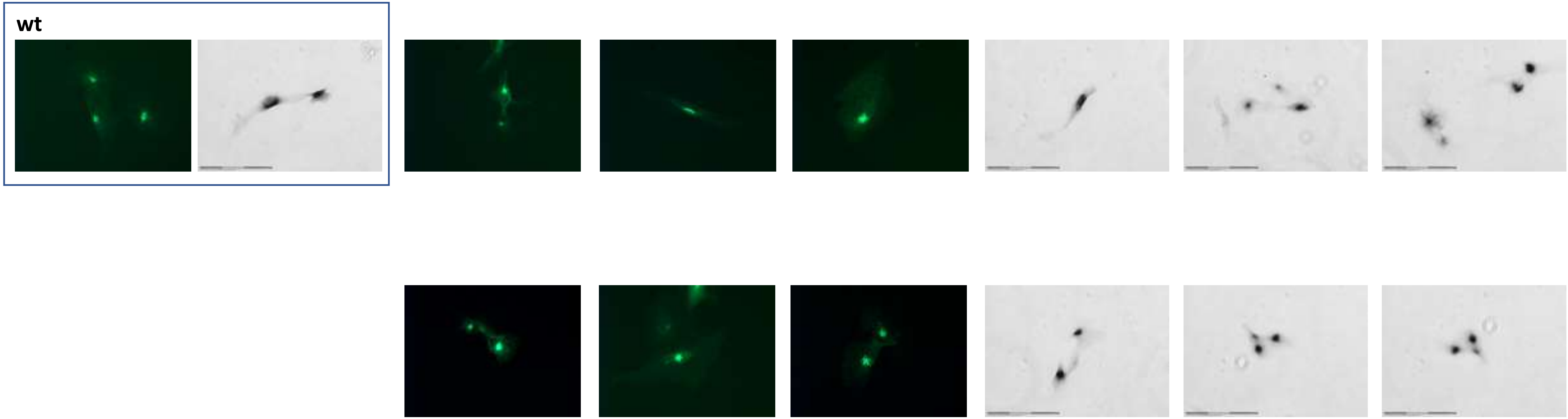

# N1270S (P-domain)

**Normal expression; significant activity**

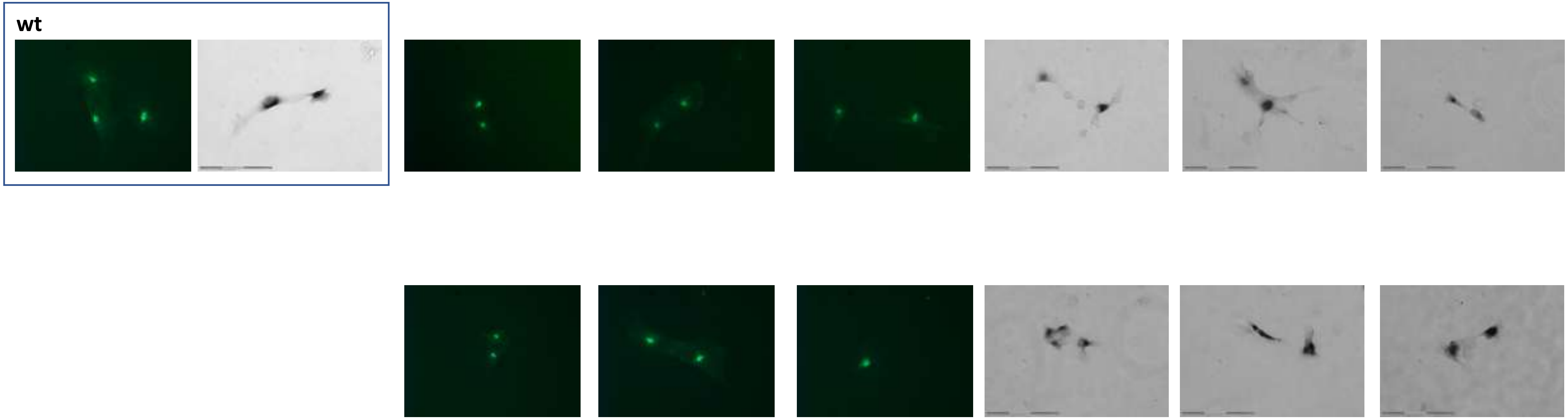

# G1266R (P-domain)

Normal expression; no activity

wt

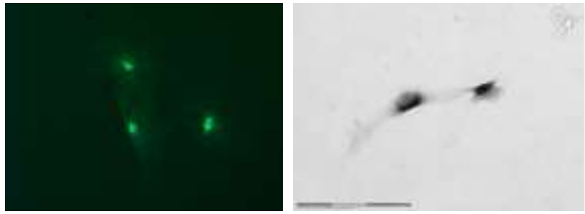

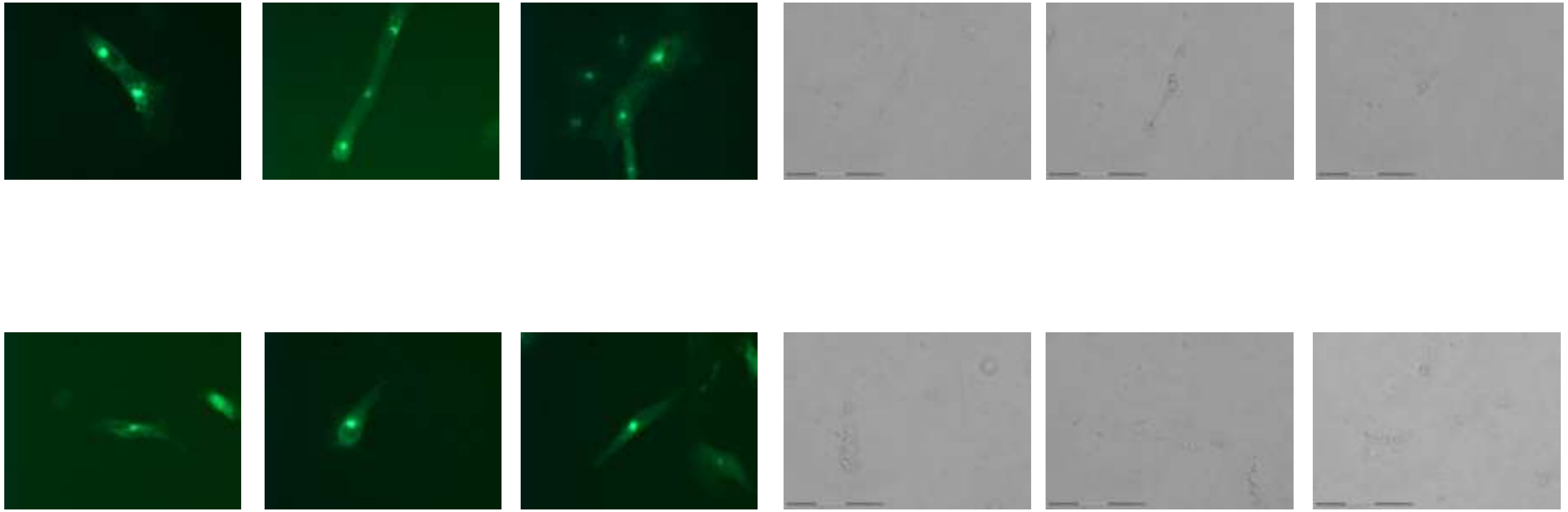

# H1247Q (P-domain)

**Normal expression; significant activity**

**wt**

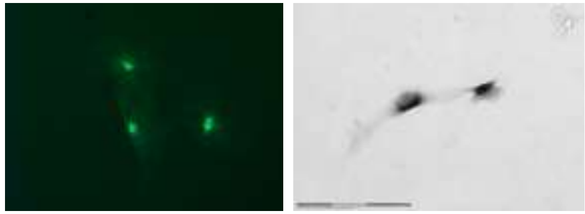

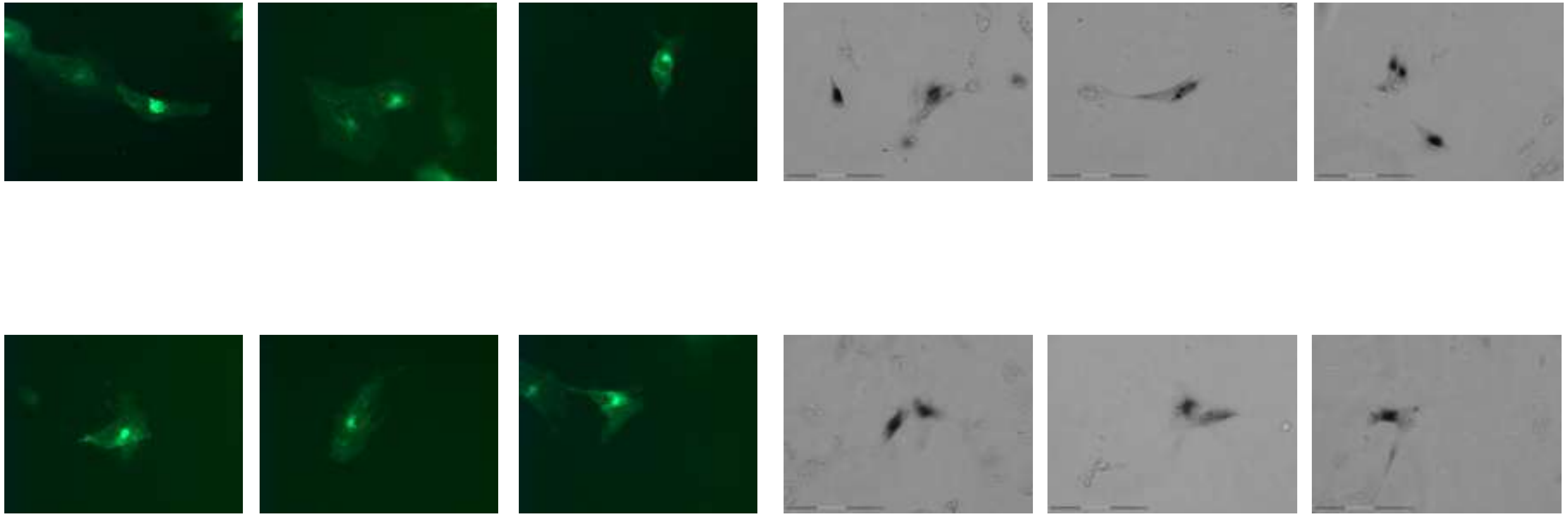

# V1239F (P-domain)

Normal expression; reduced activity

wt

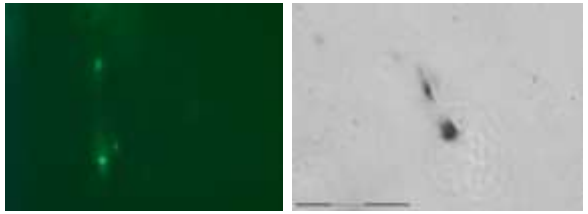

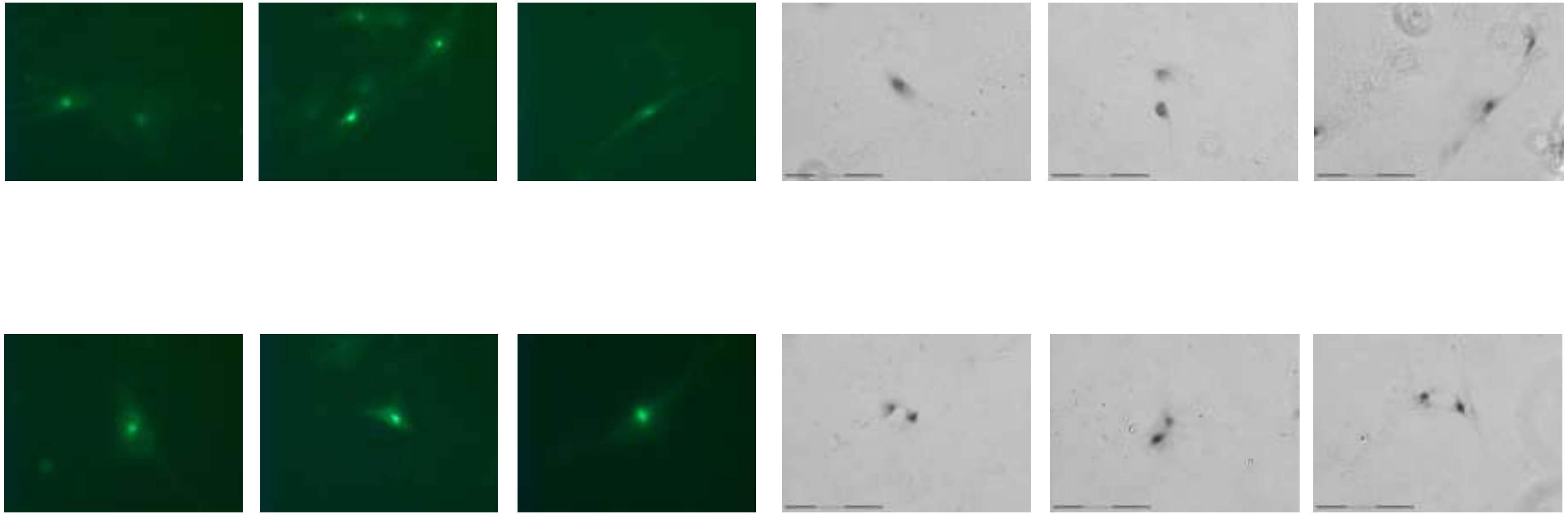

# I1230V (P-domain)

Normal expression; significant activity

wt

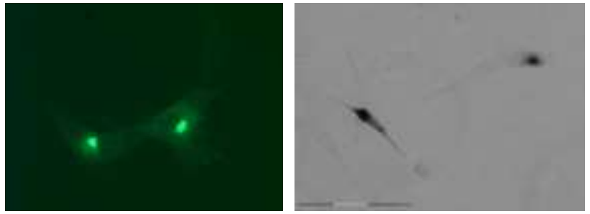

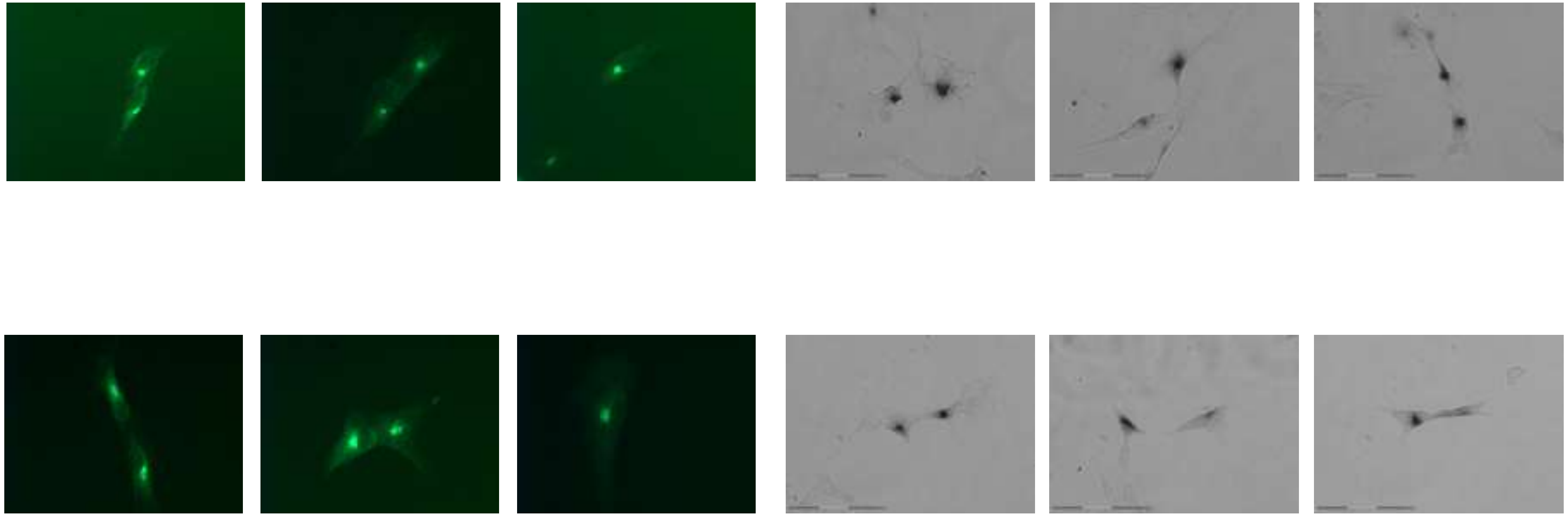

# R1224W (P-domain)

**Normal expression; significant activity**

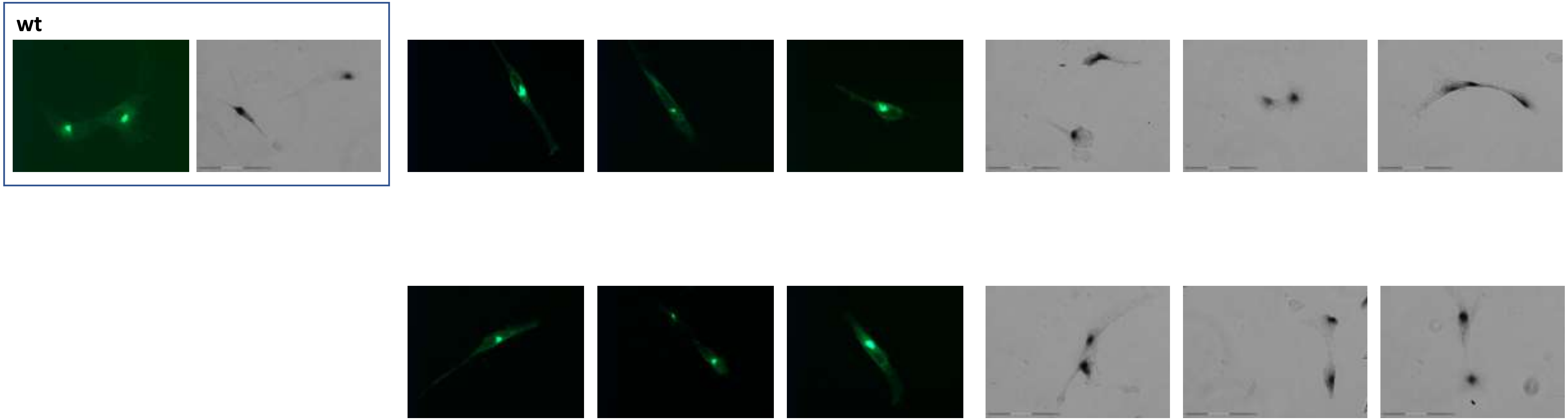

# V1216M (P-domain)

**Reduced expression; significant activity**

**wt**

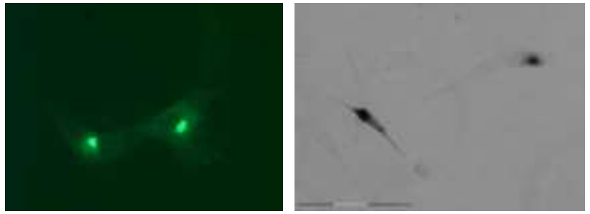

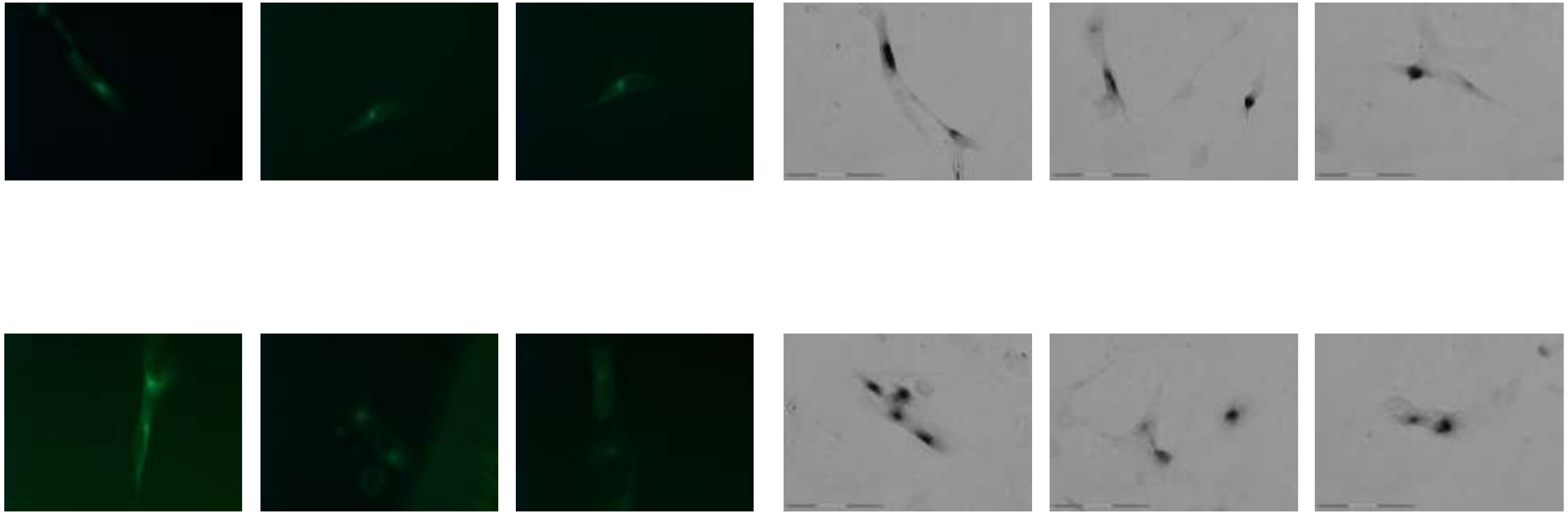

# H1207R (P-domain)

**Normal expression; significant activity**

**wt**

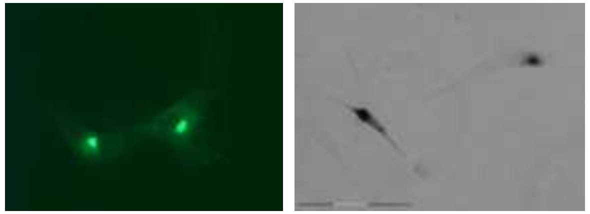

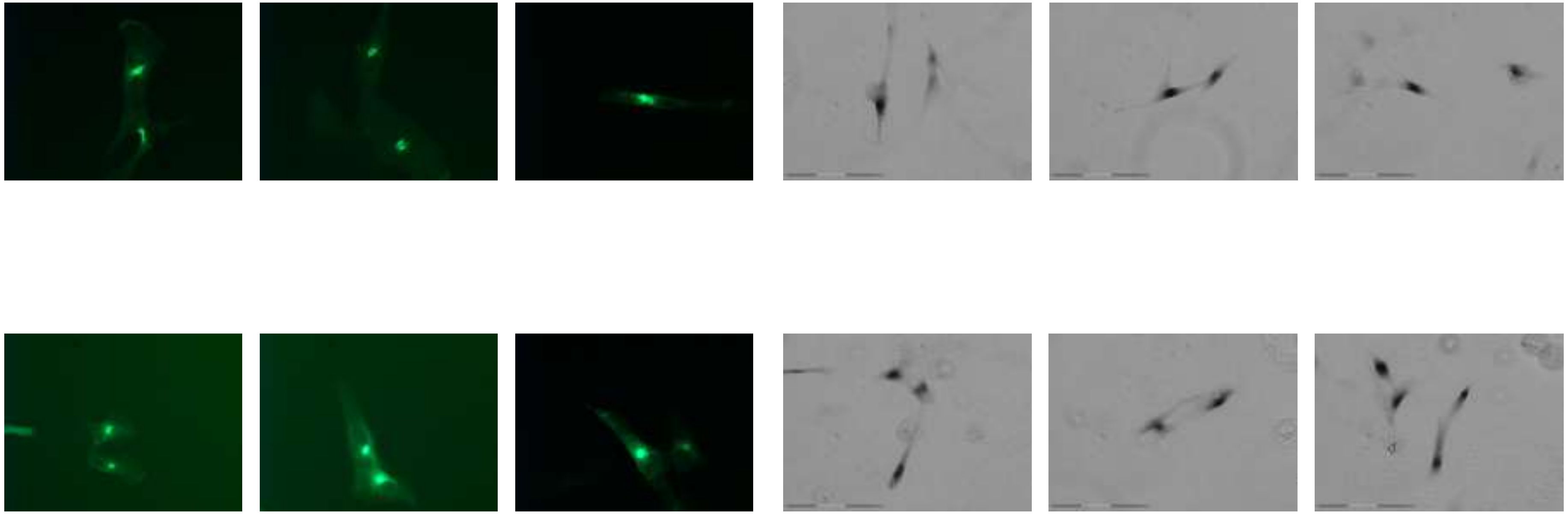

# A1195T (P-domain)

**Normal expression; reduced activity**

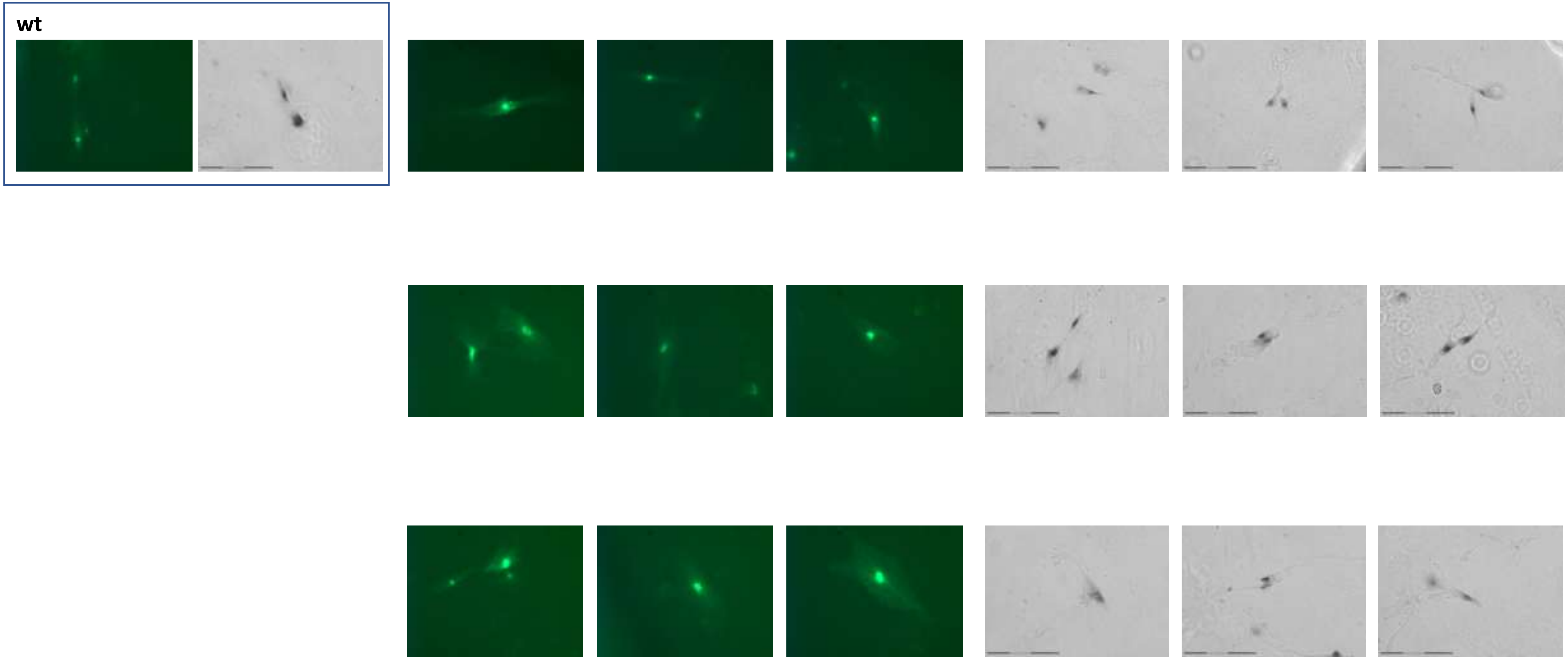

# A1168S (N-domain)

**Normal expression; significant activity**

**wt**

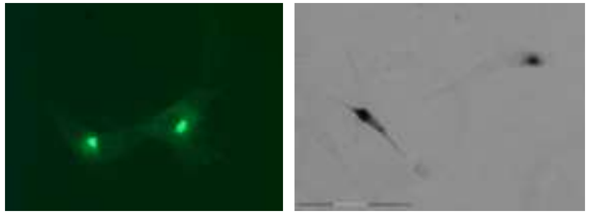

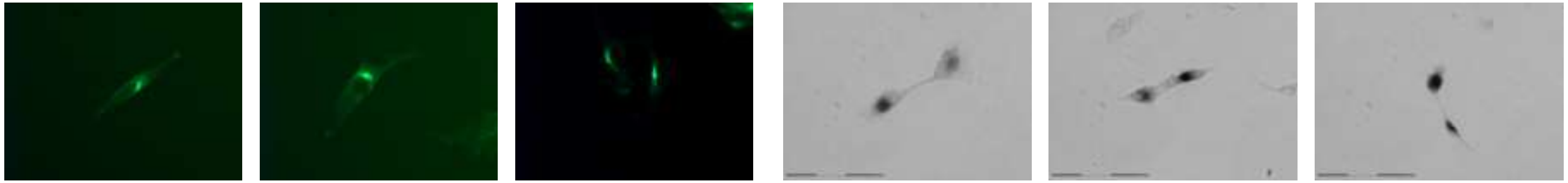

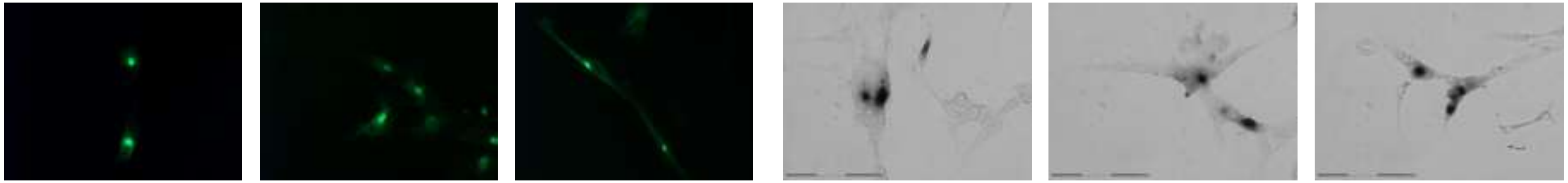

# G1158R (N-domain)

Reduced expression; significant activity

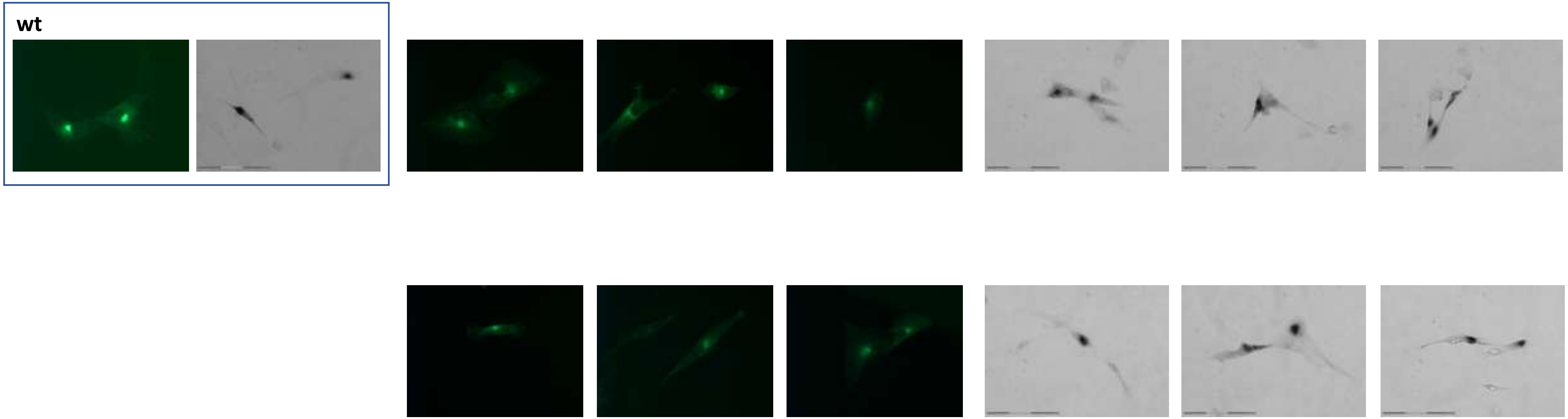

# R1156H (N-domain)

**Normal expression; significant activity**

**wt**

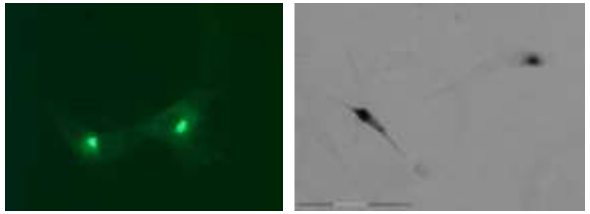

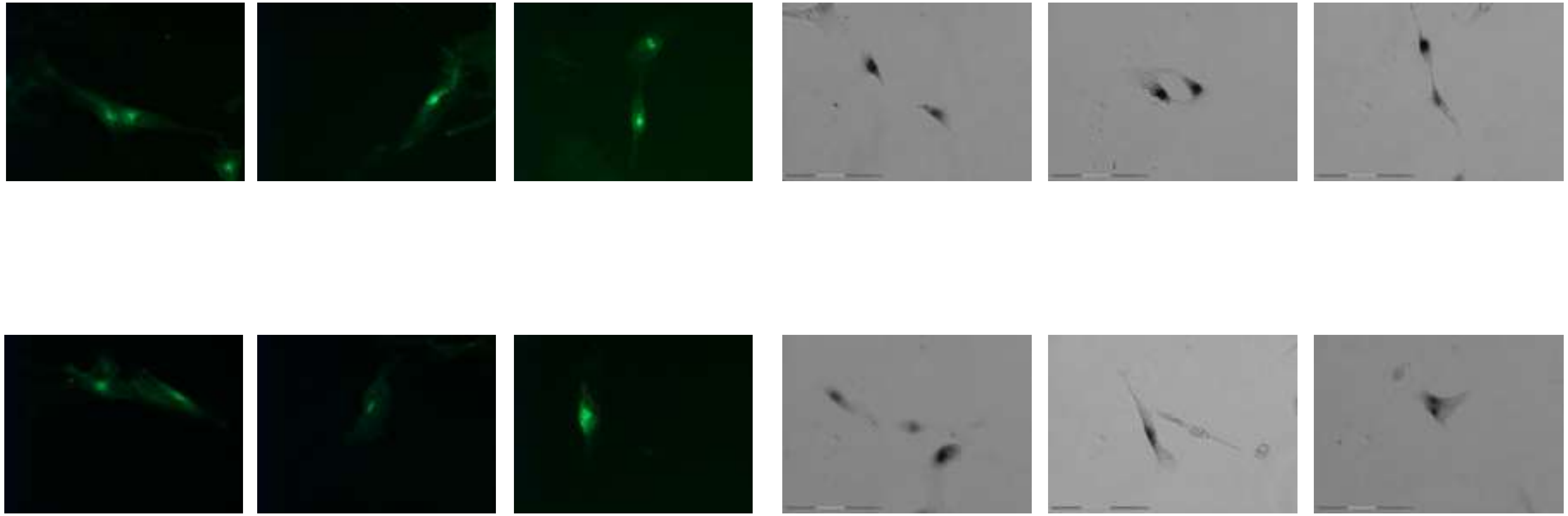

# R1151H (N-domain)

**Reduced expression; reduced activity**

**wt**

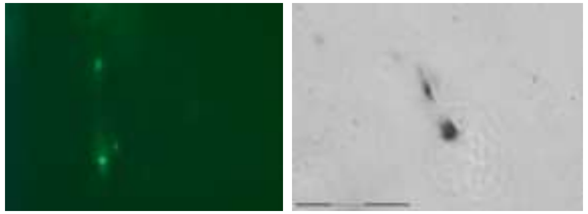

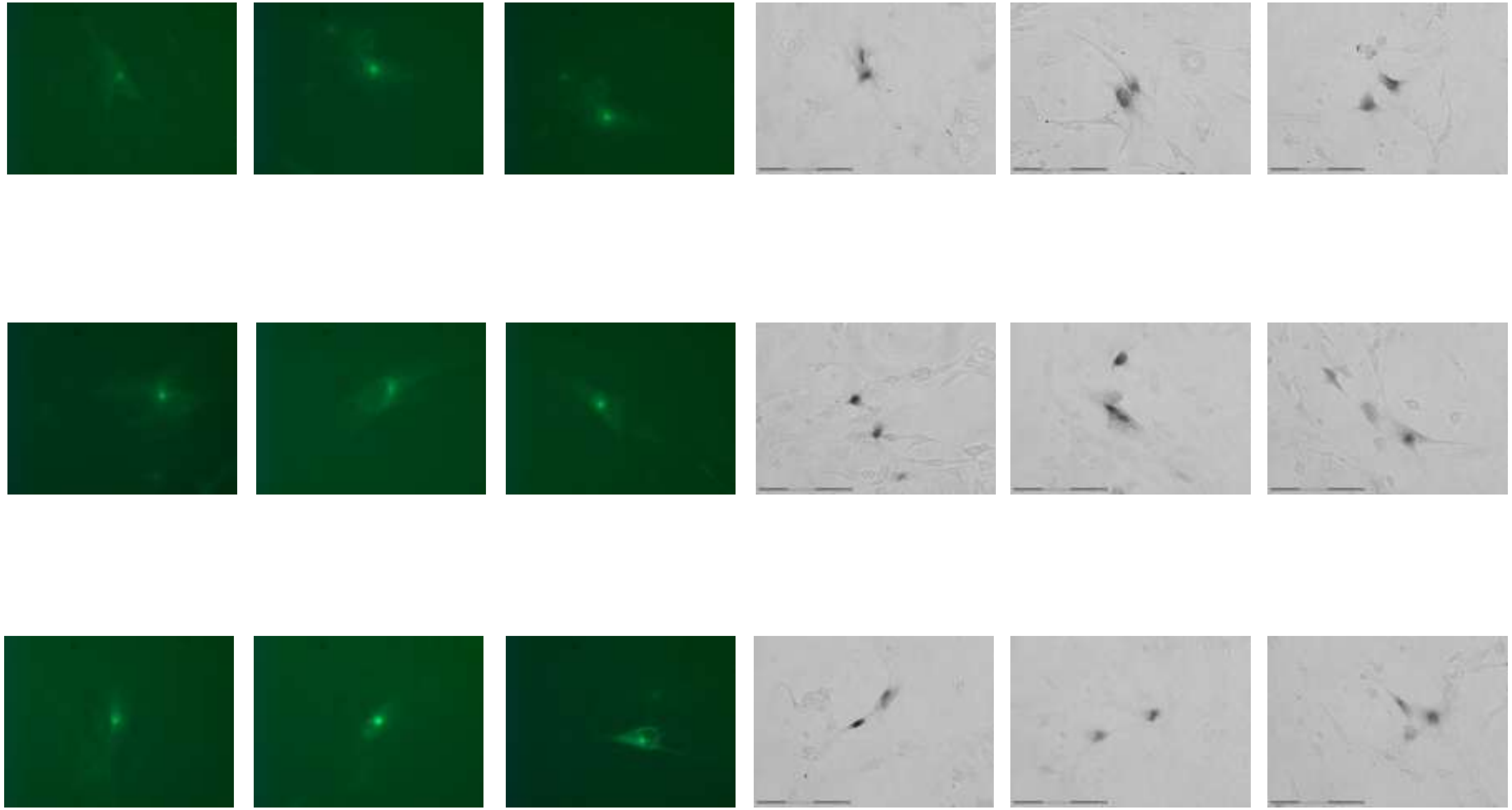

# R1151C (N-domain)

Reduced expression; significant activity

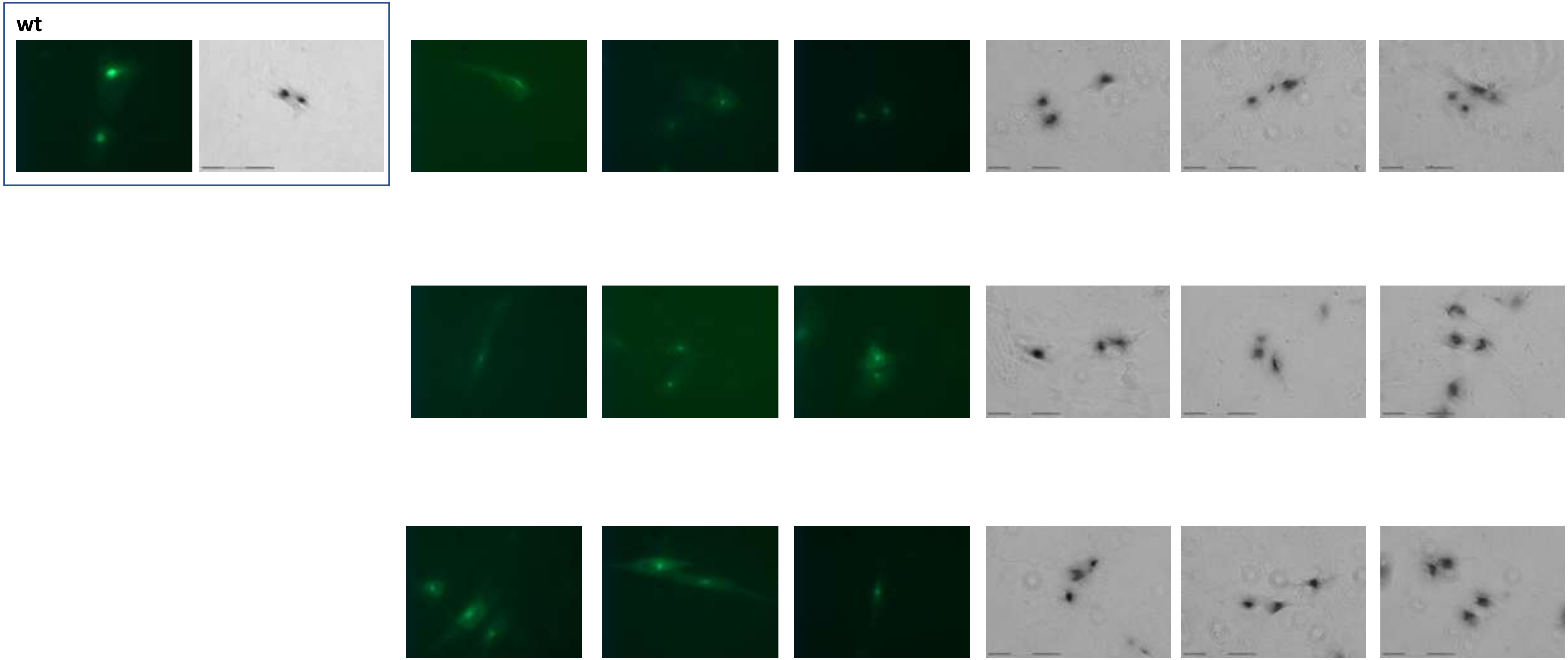

# I1148T(N-domain)

Reduced expression; significant activity

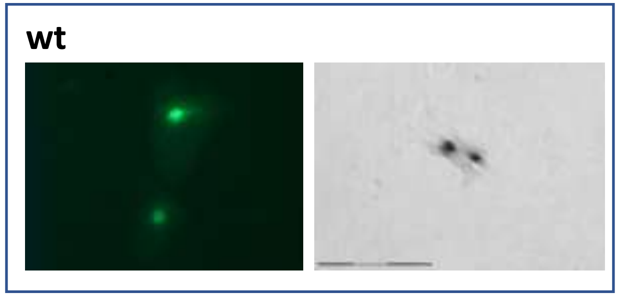


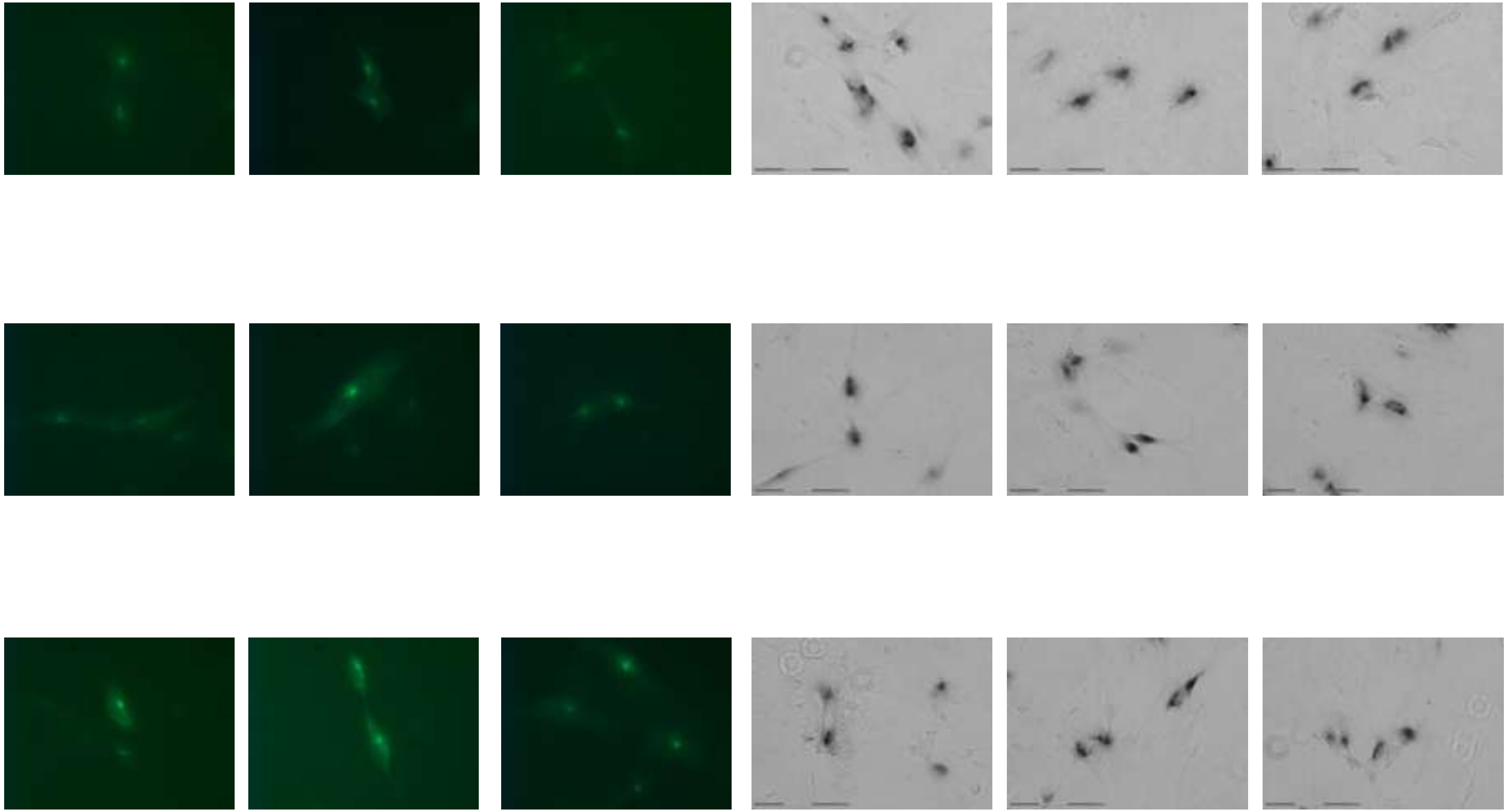

# Q1142H (N-domain)

Normal expression; significant activity

**wt**

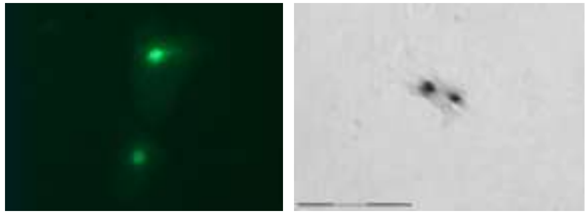

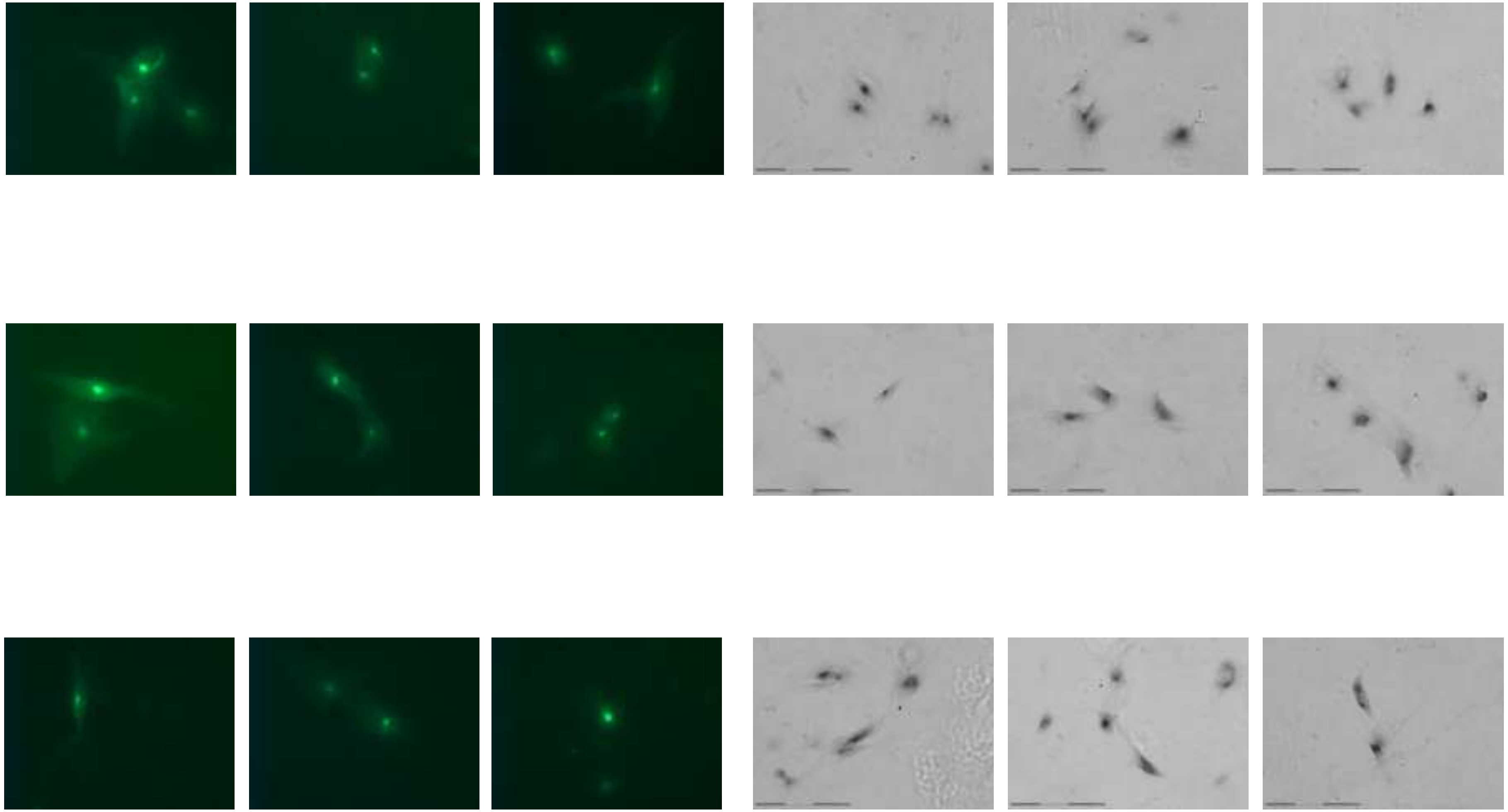

# A1135T(N-domain)

Normal expression; significant activity

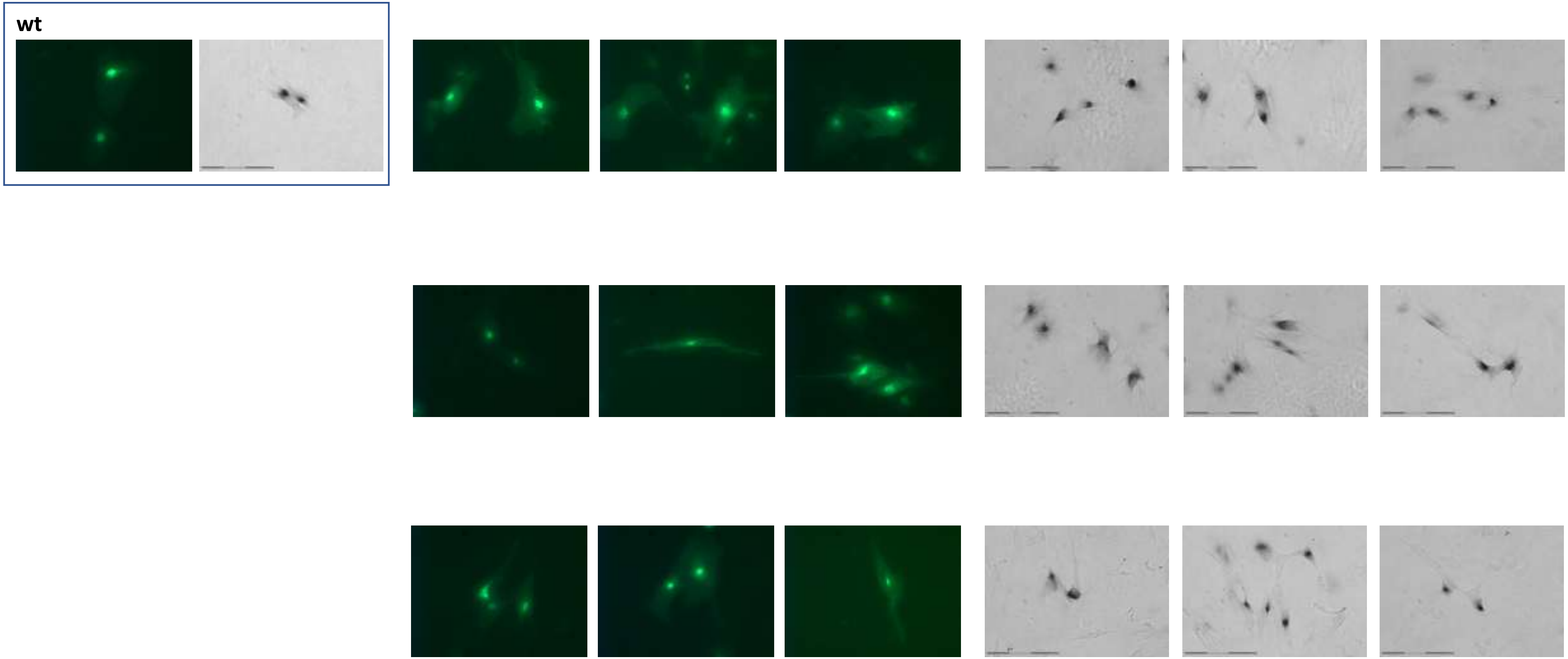

# P1123L (N-domain)

**Normal expression; significant activity**

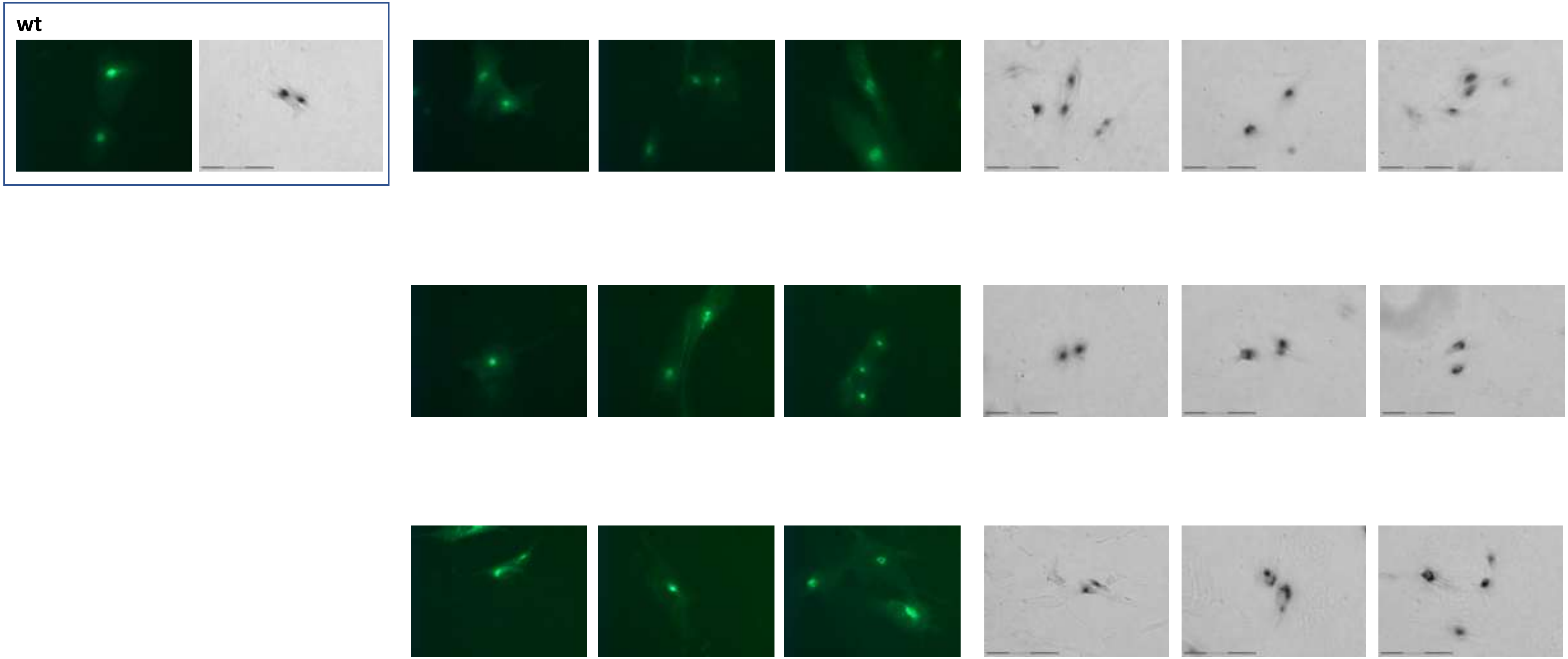

# G1111D (N-domain)

**Reduced expression; reduced activity**

**wt**

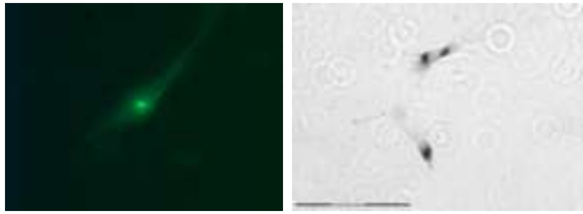

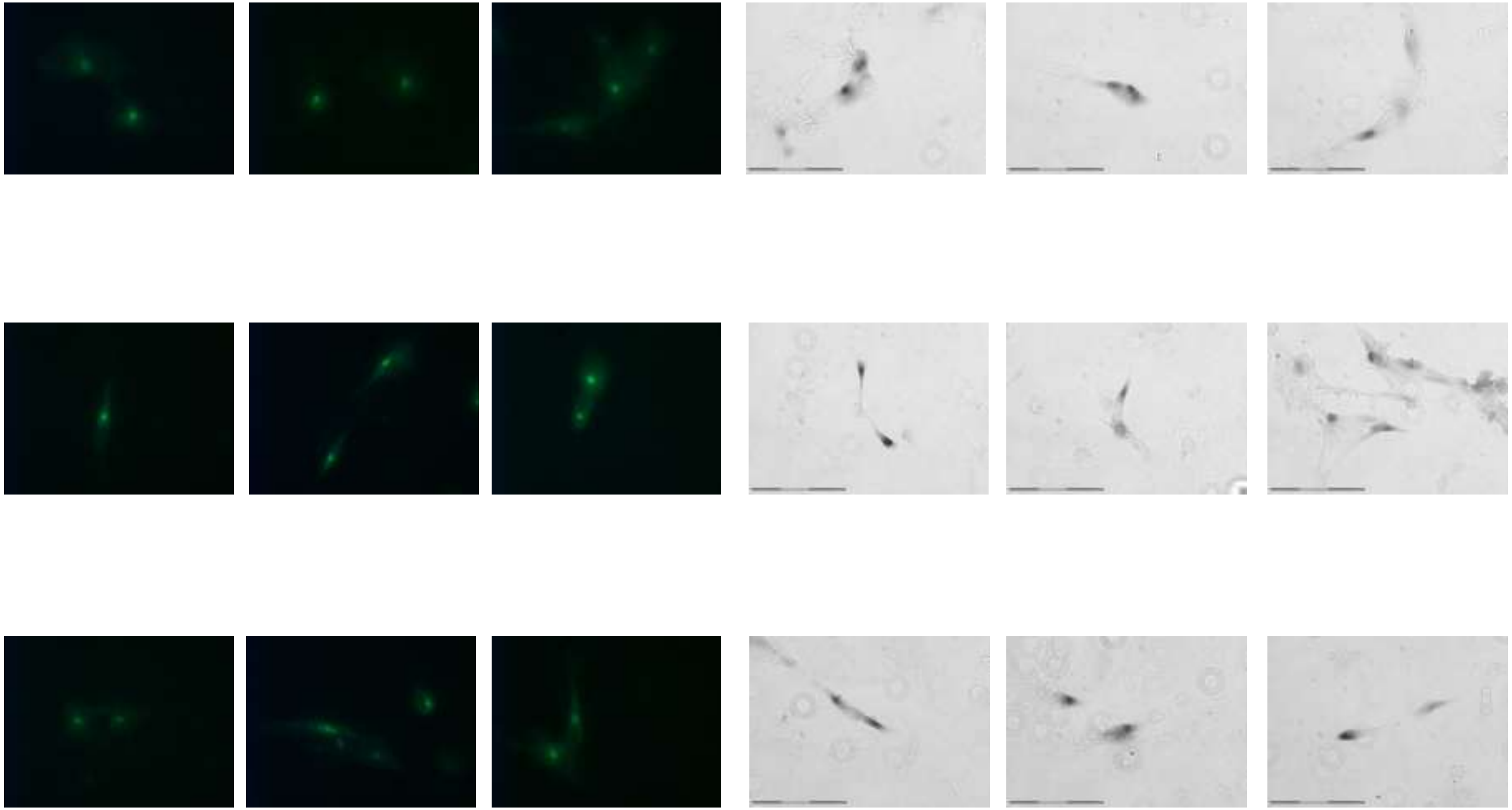

# V1109M (N-domain)

**Reduced expression; reduced activity**

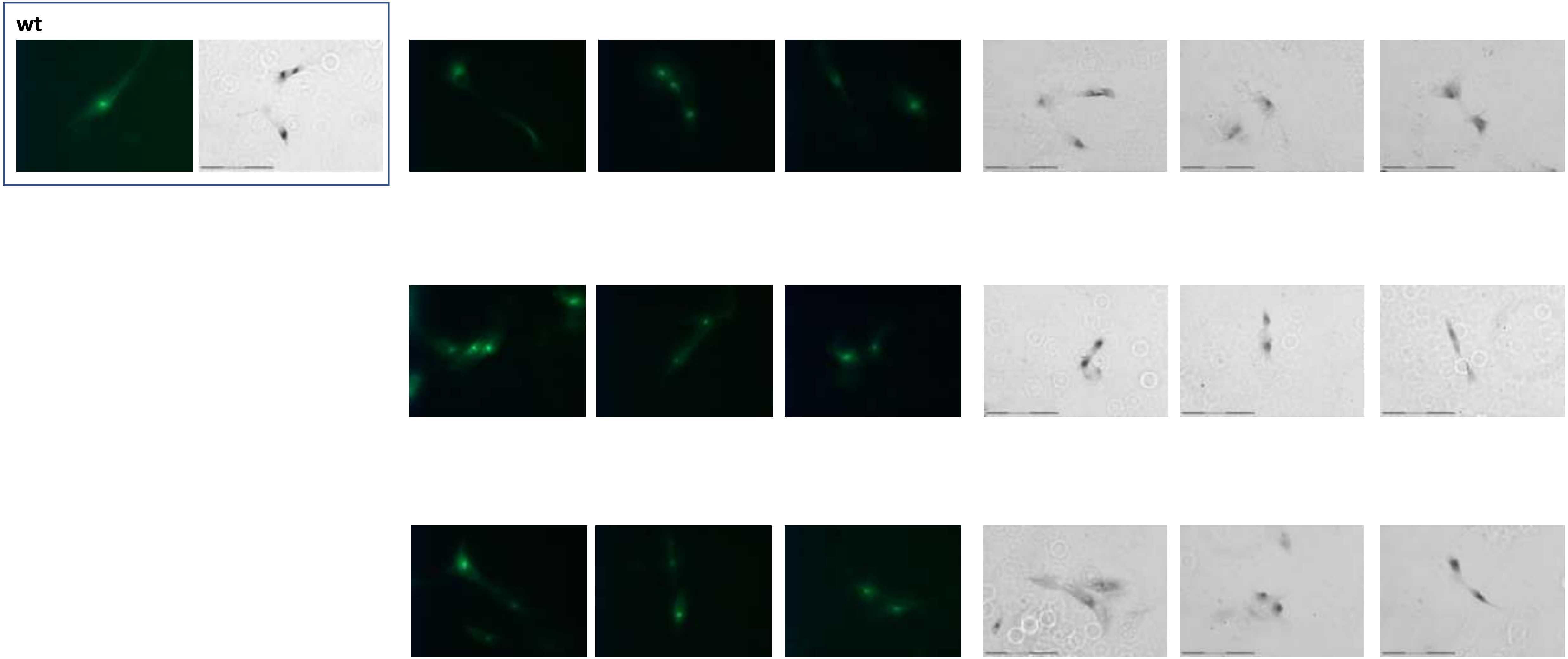

# V1106I (N-domain)

**Reduced expression; reduced activity**

**wt**

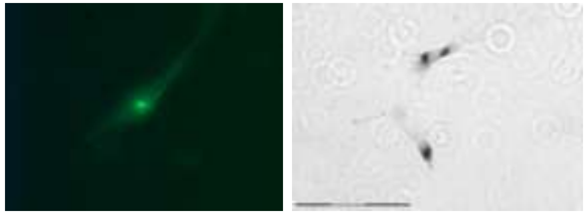

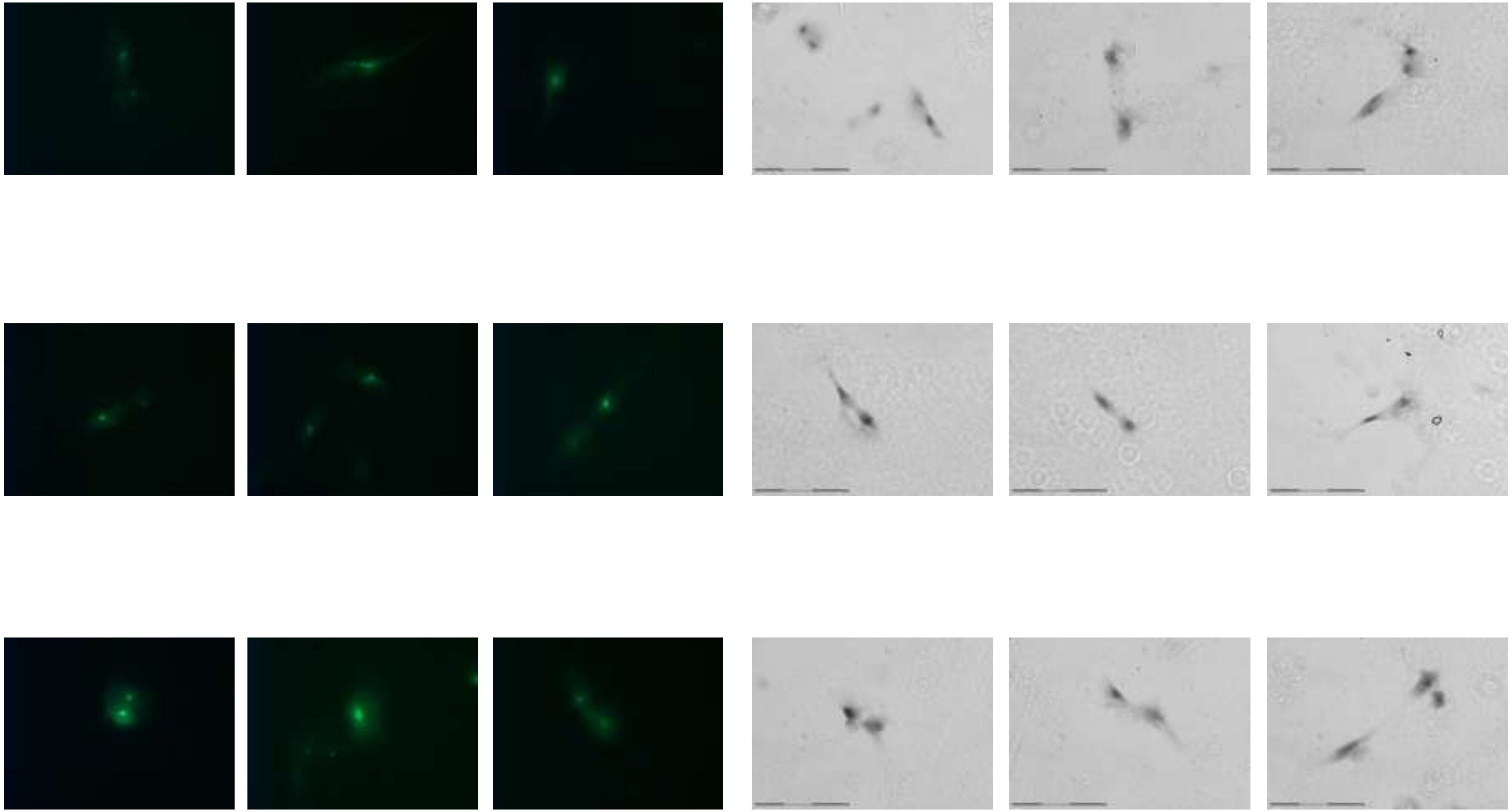

# I1102T (N-domain)

**Reduced expression; reduced activity**

**wt**

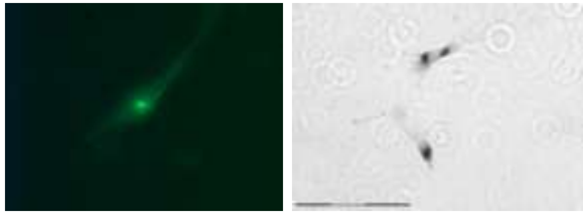

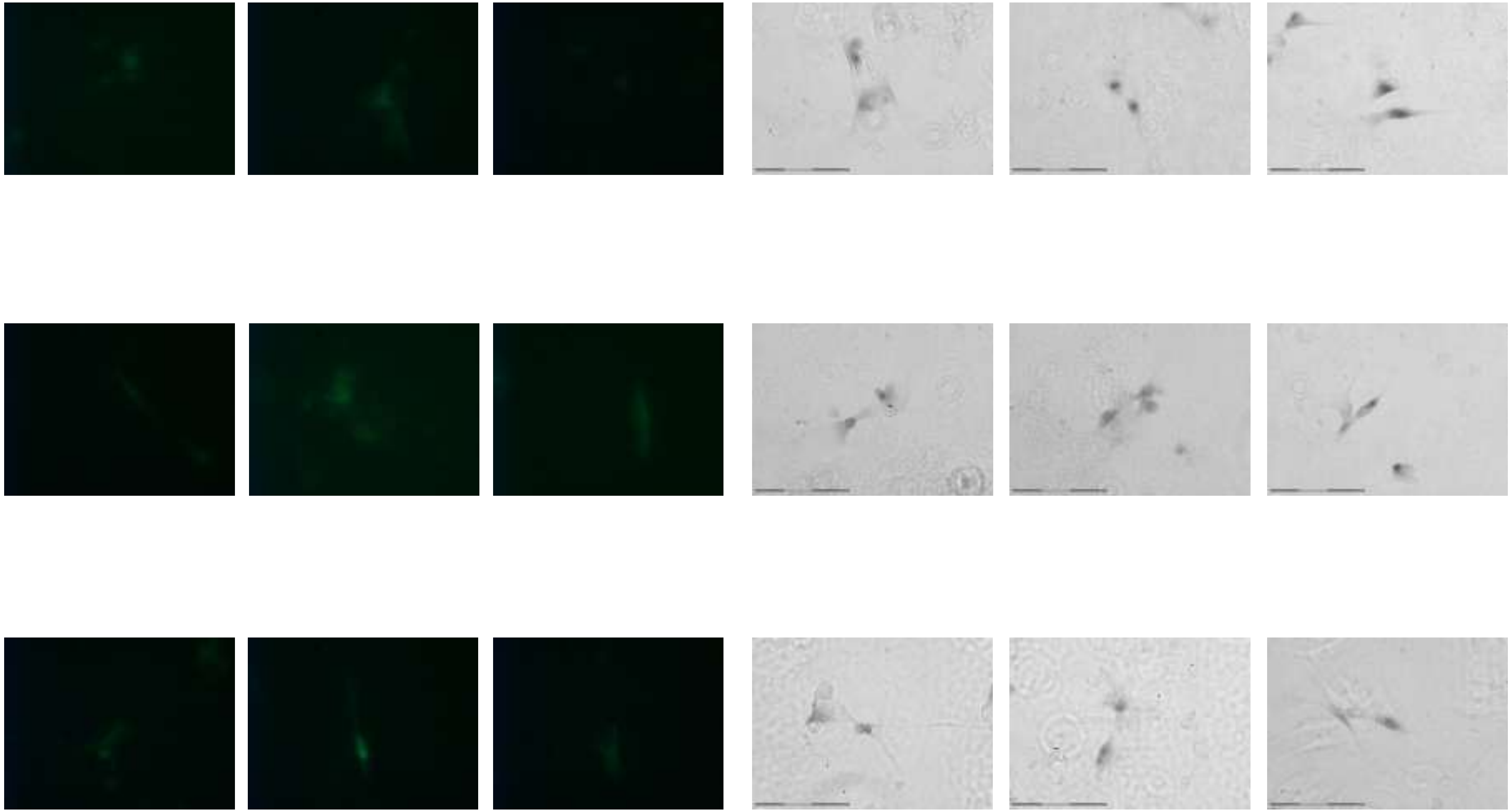

# L1083F (N-domain)

**Reduced expression; reduced activity**

**wt**

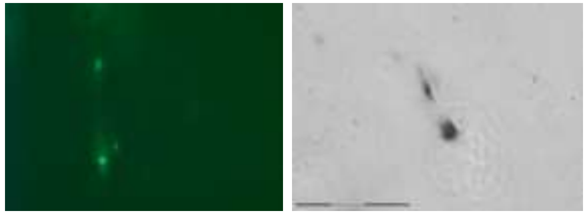

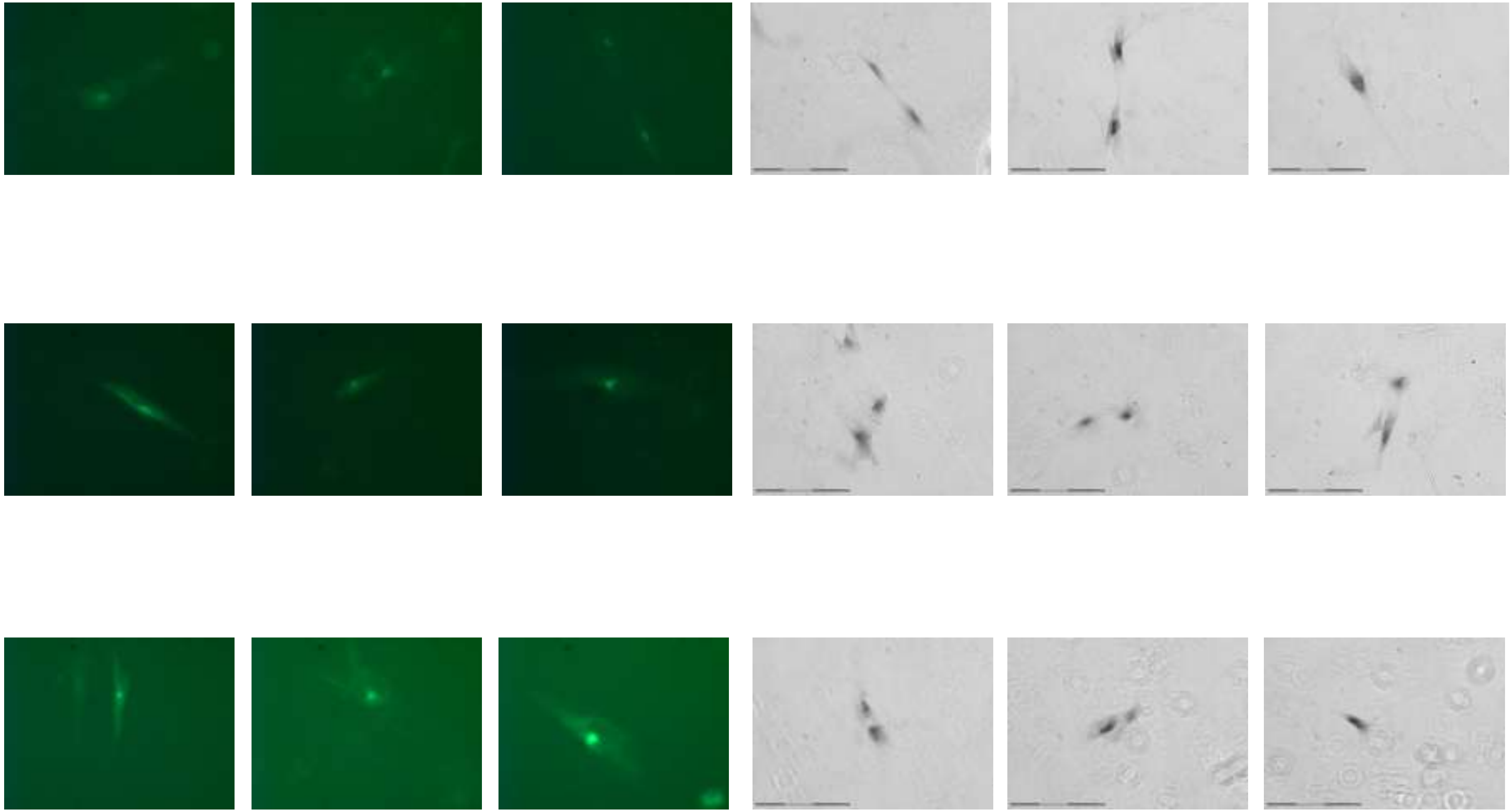

# H1069Q (N-domain)

Reduced expression; significant activity

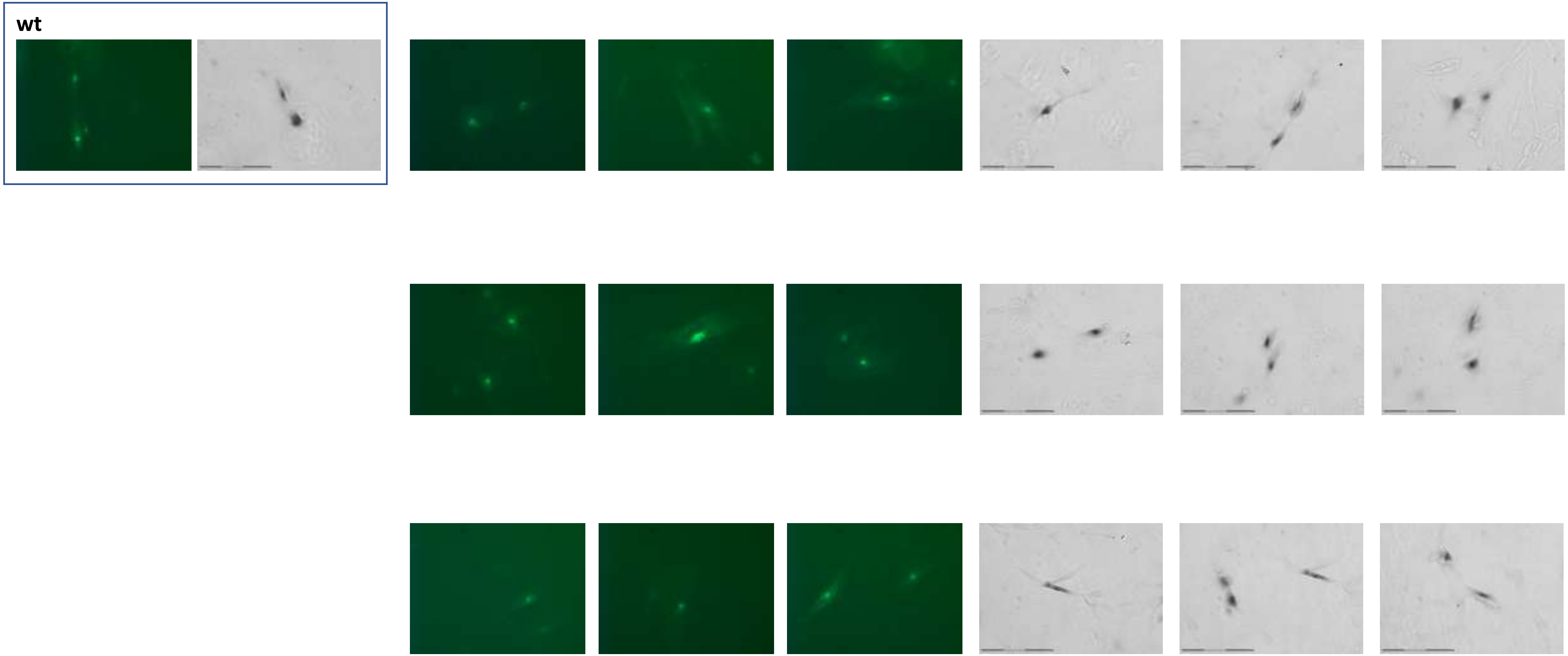

# G1061E (N-domain)

**Reduced expression; no activity**

**wt**

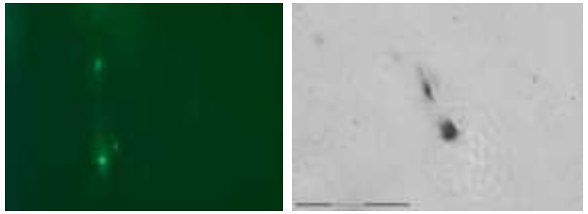

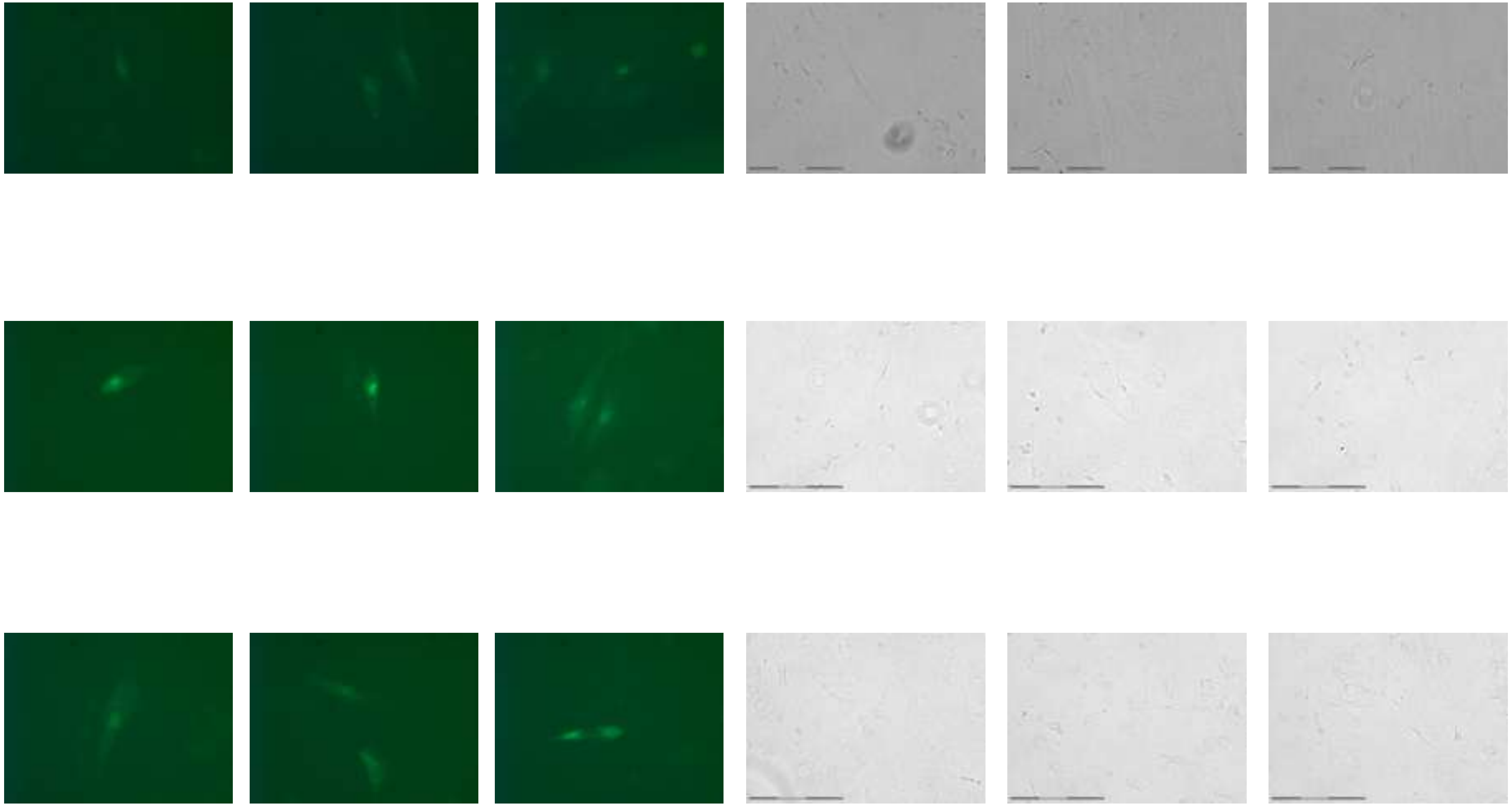

# V1036I (N-domain)

**Normal expression; significant activity**

**wt**

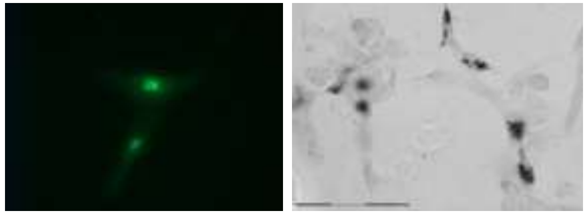

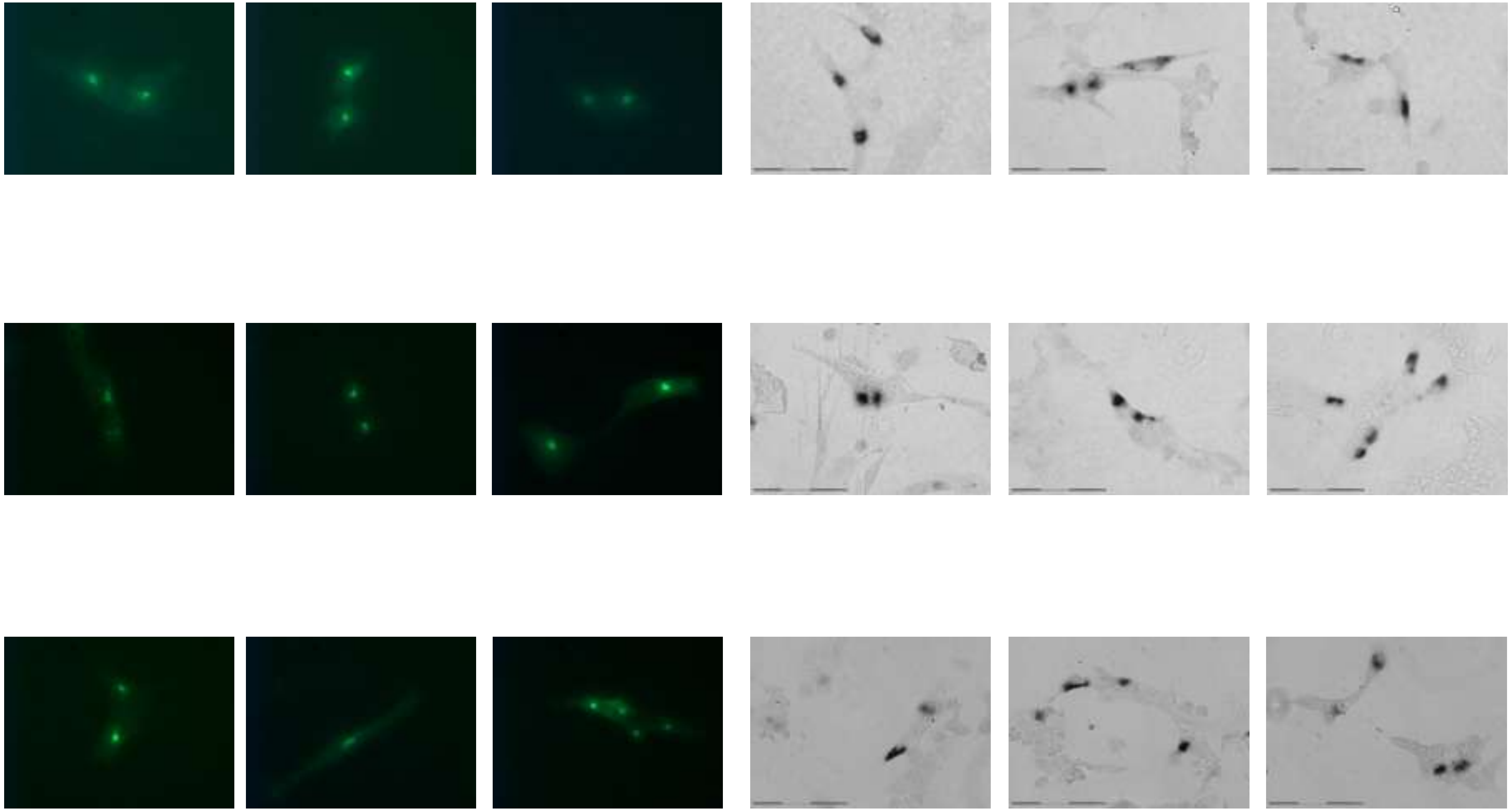

# H1034R (P-domain)

**Normal expression; significant activity**

**wt**

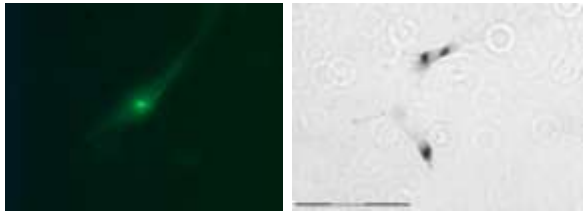

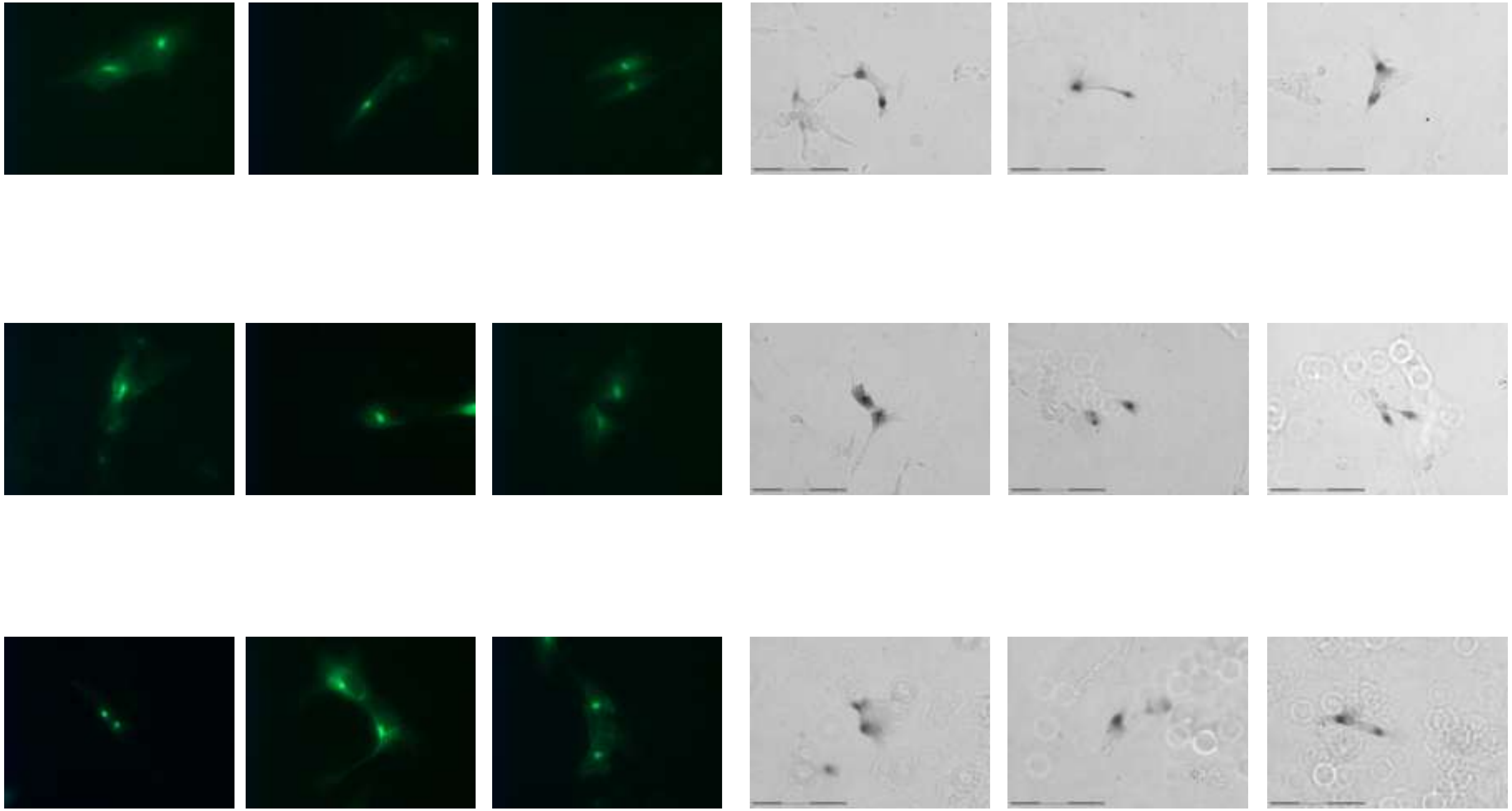

# A1018V (P-domain)

**Reduced expression; significant activity**

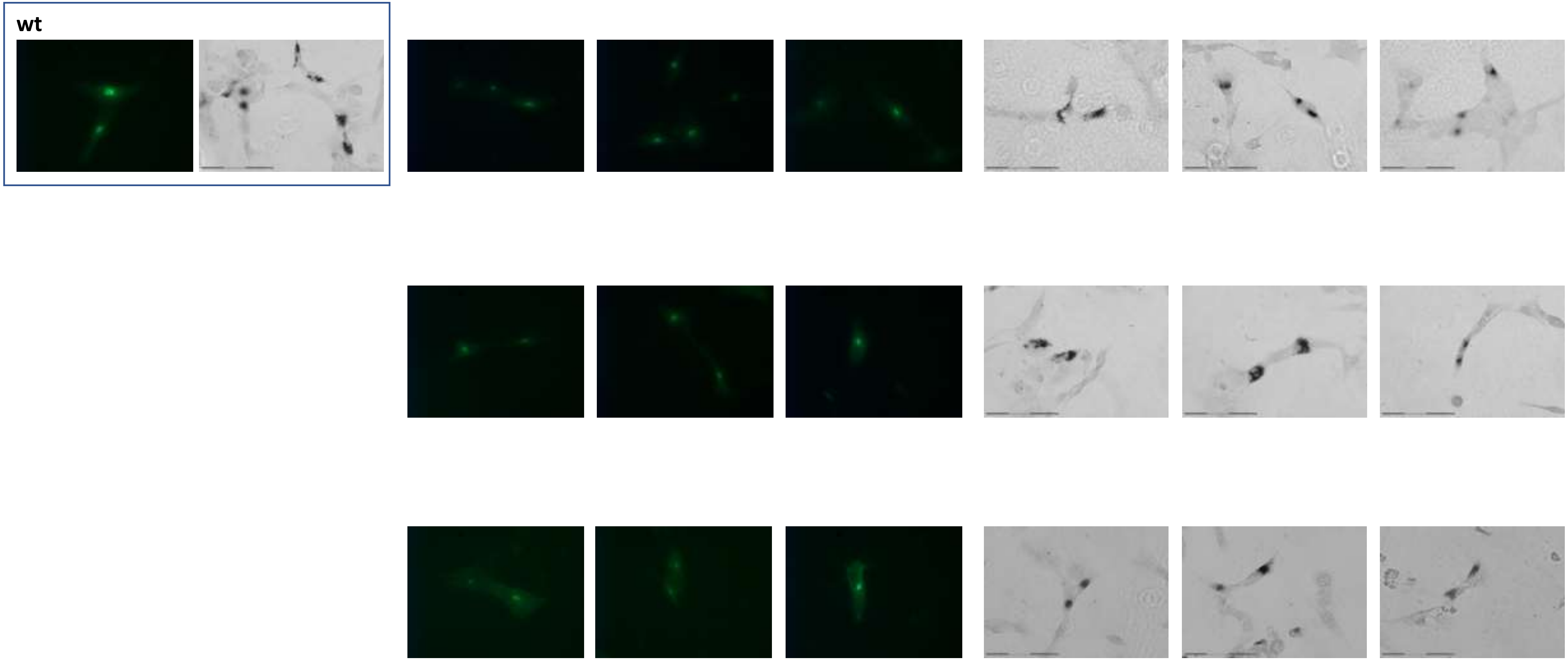

# A1003V (P-domain)

**Reduced expression; reduced activity**

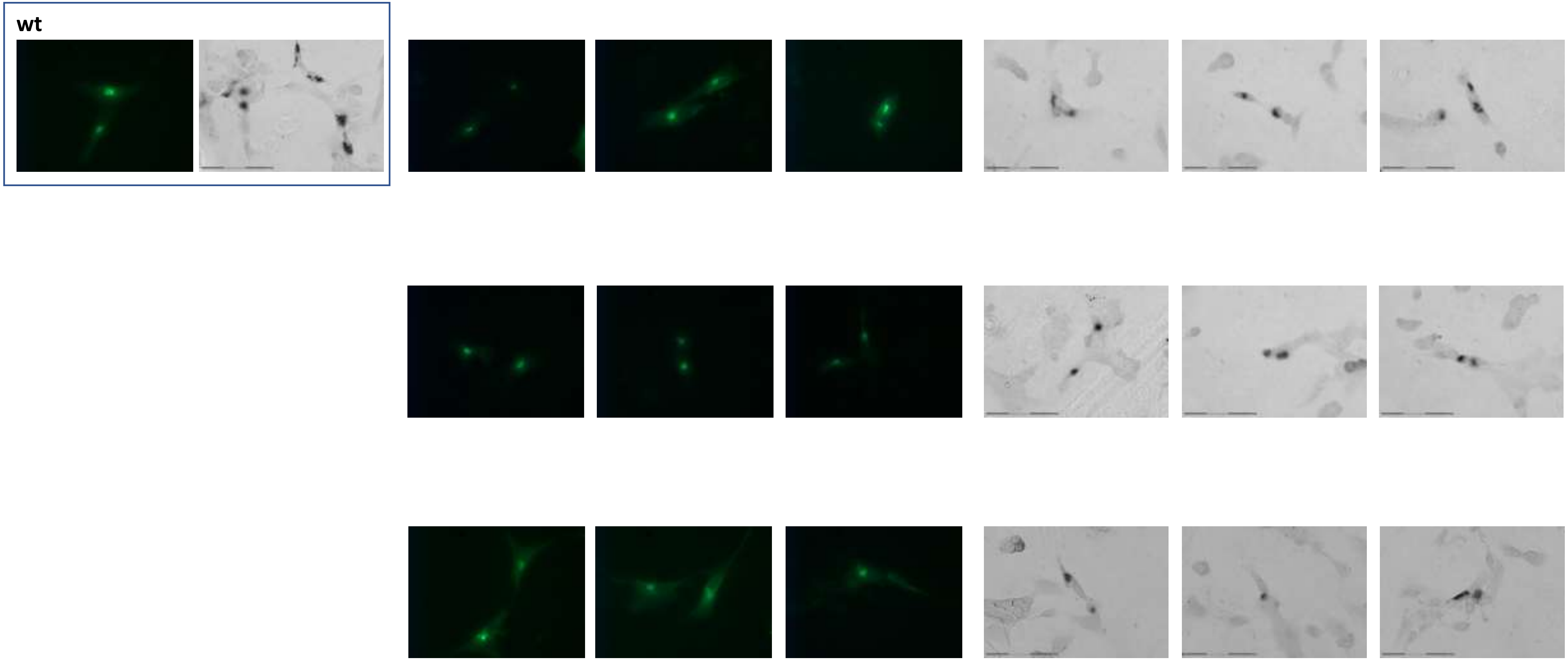

# V995A (TM6)

**Reduced expression; significant activity**

**wt**

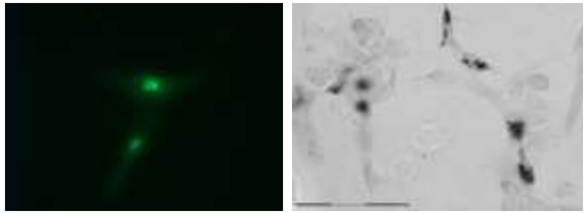

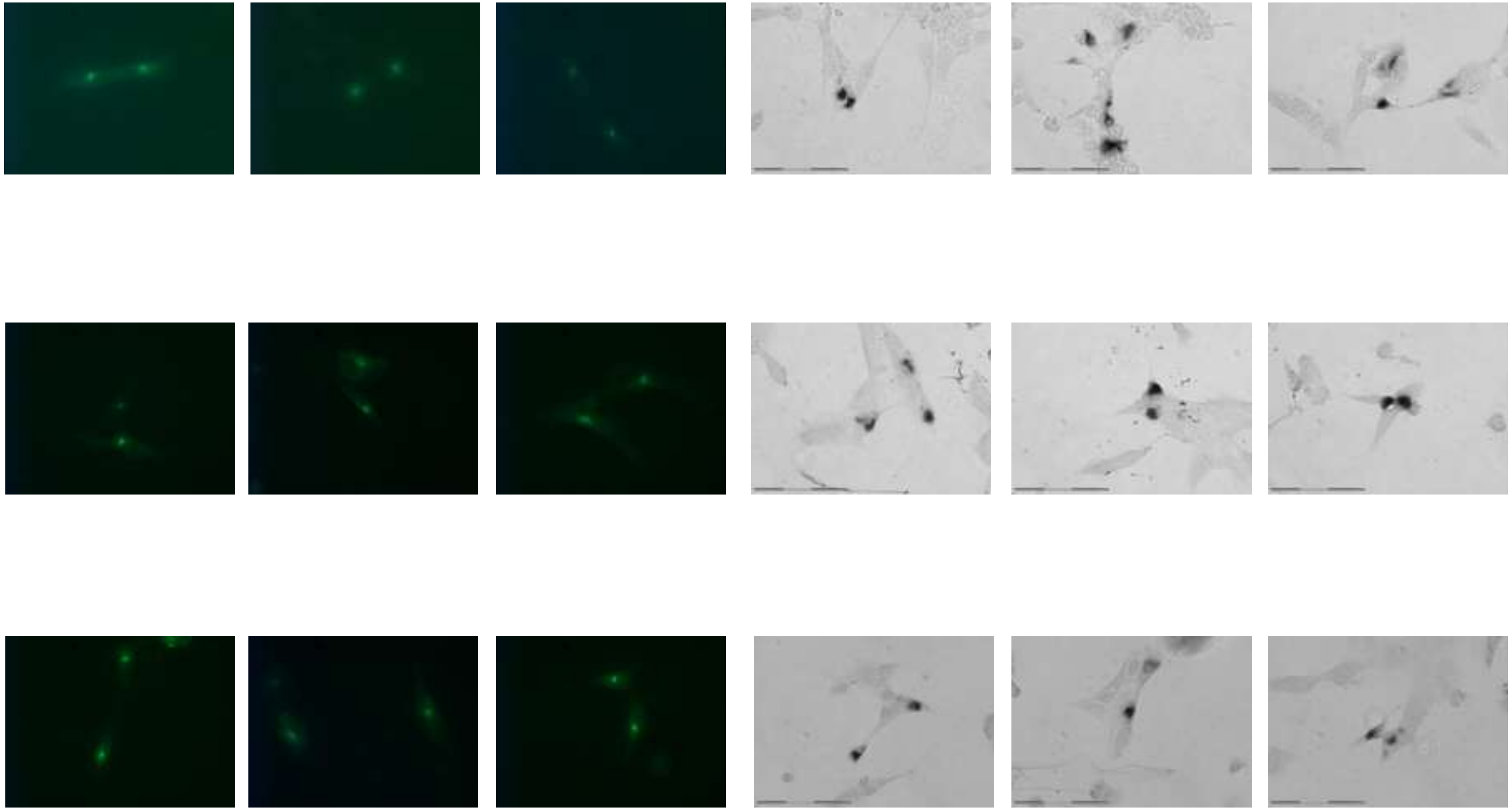

# T993M (TM6)

**Normal expression; reduced activity**

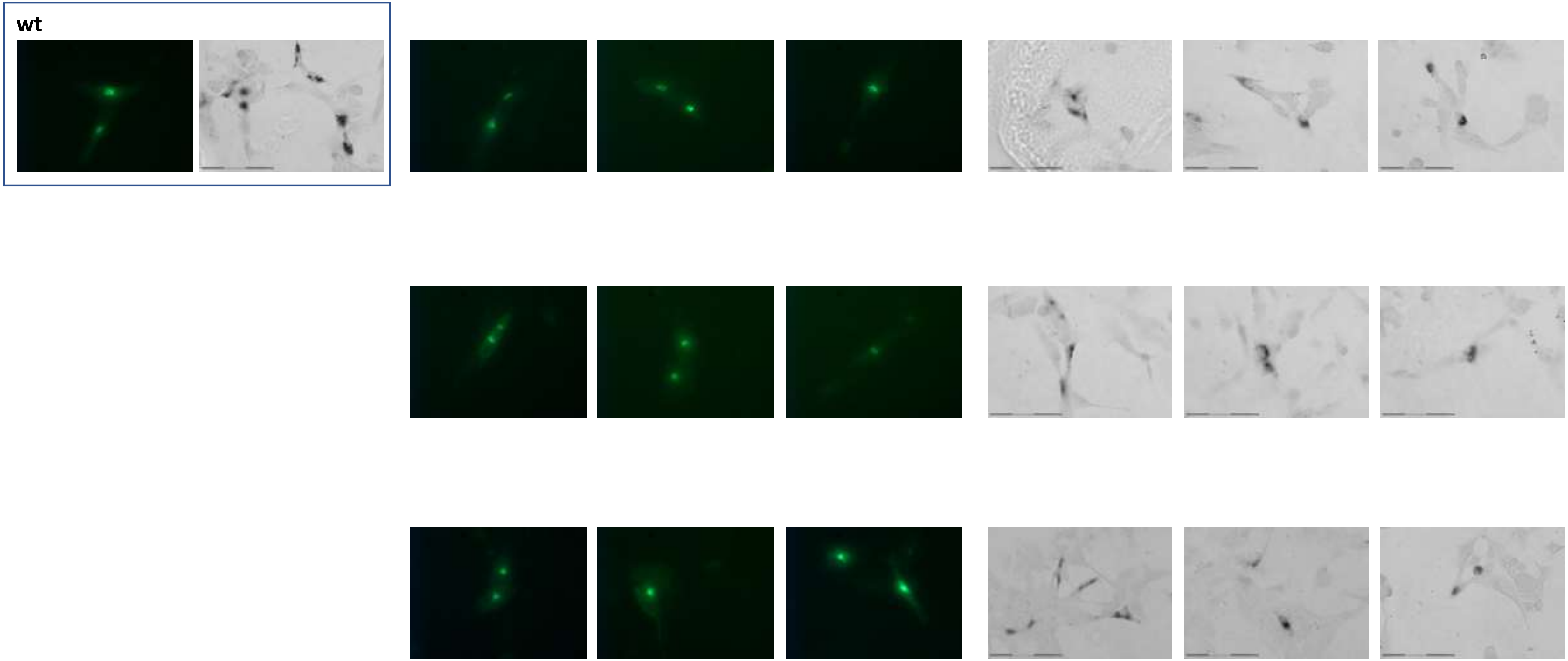

# P992L (TM6)

**Reduced expression; reduced activity**

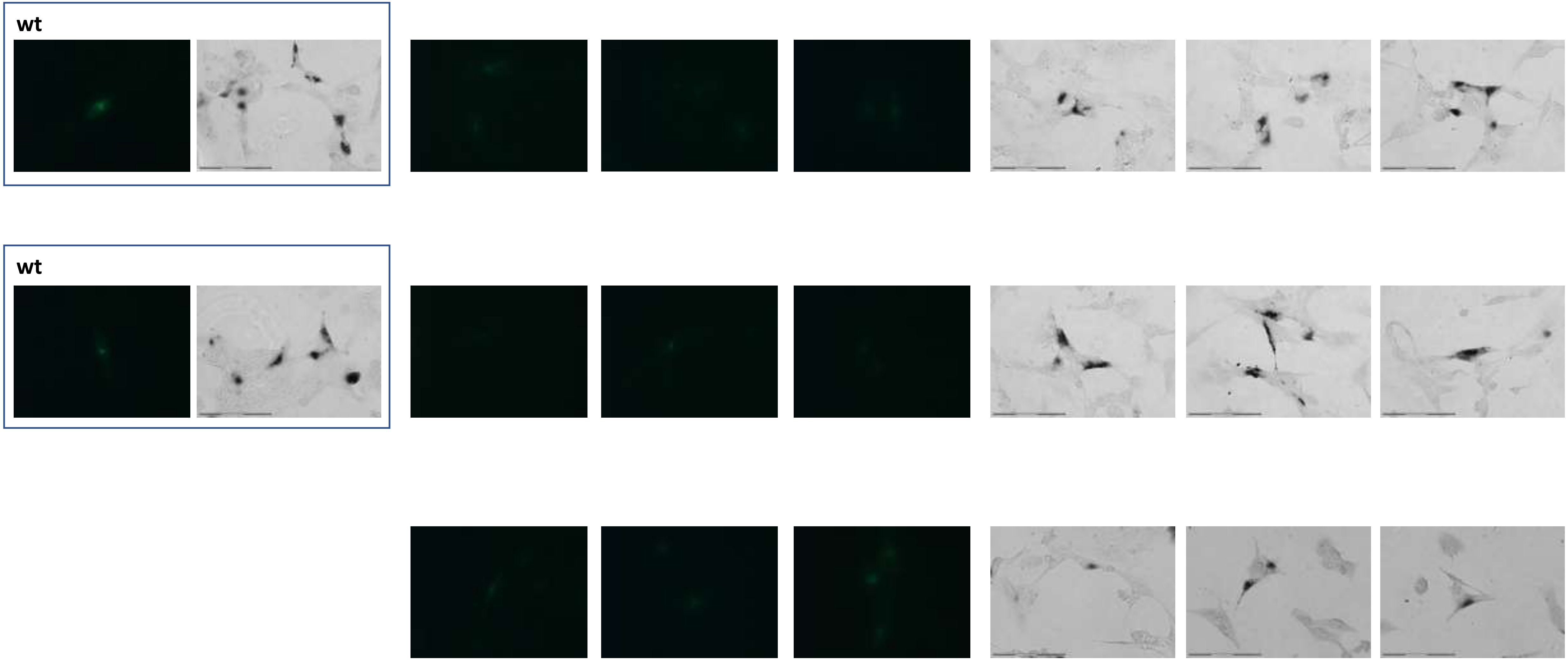

# T991M (TM6)

**Reduced expression; reduced activity**

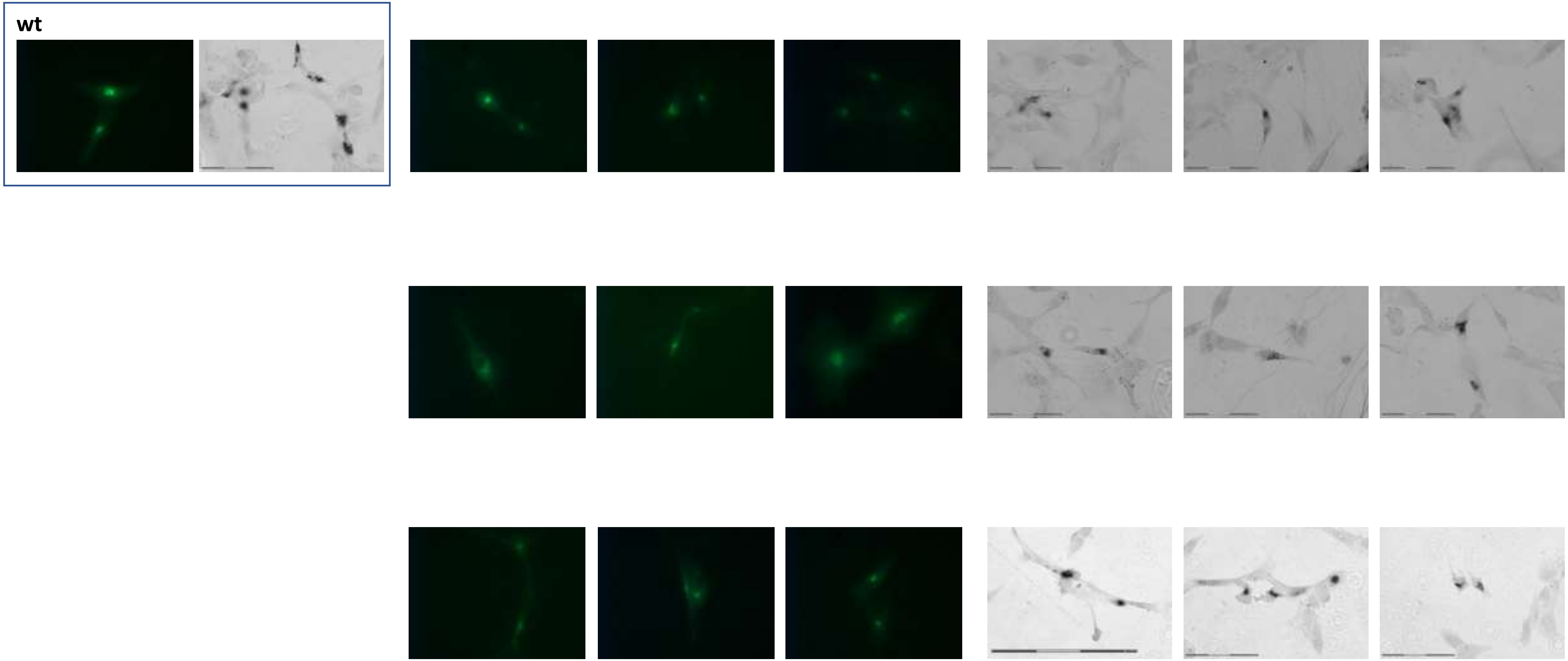

# T977M (TM6)

**Reduced expression; reduced activity**

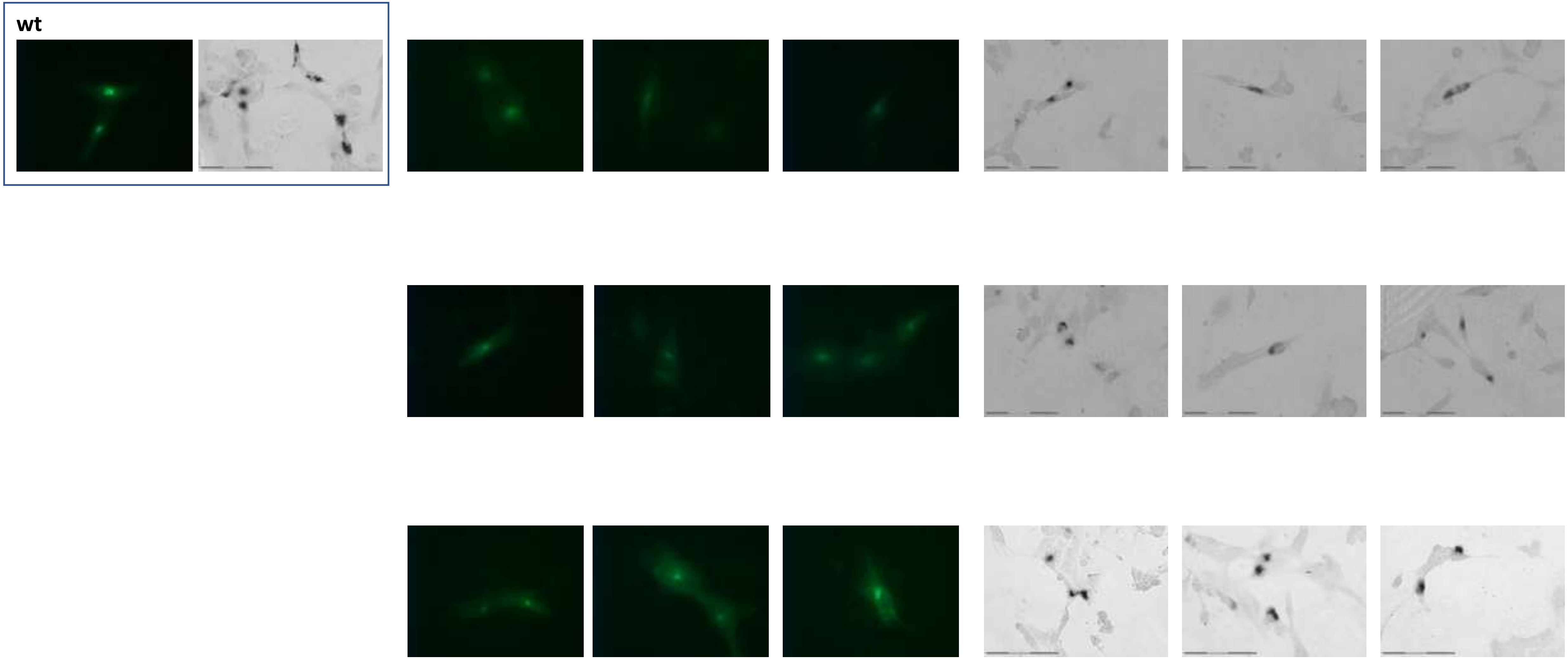

# S975Y (TM6)

Reduced expression; significant activity

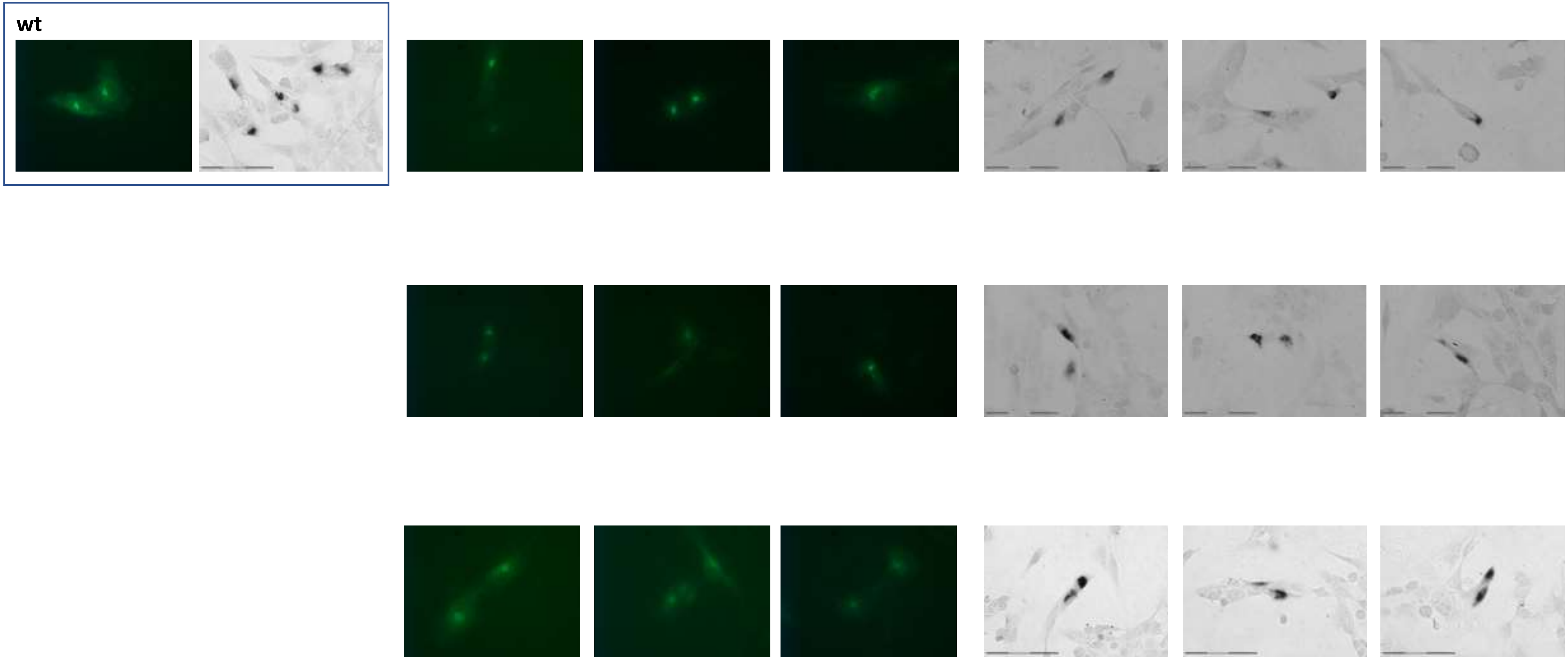

# T974M (TM6)

Reduced expression; significant activity

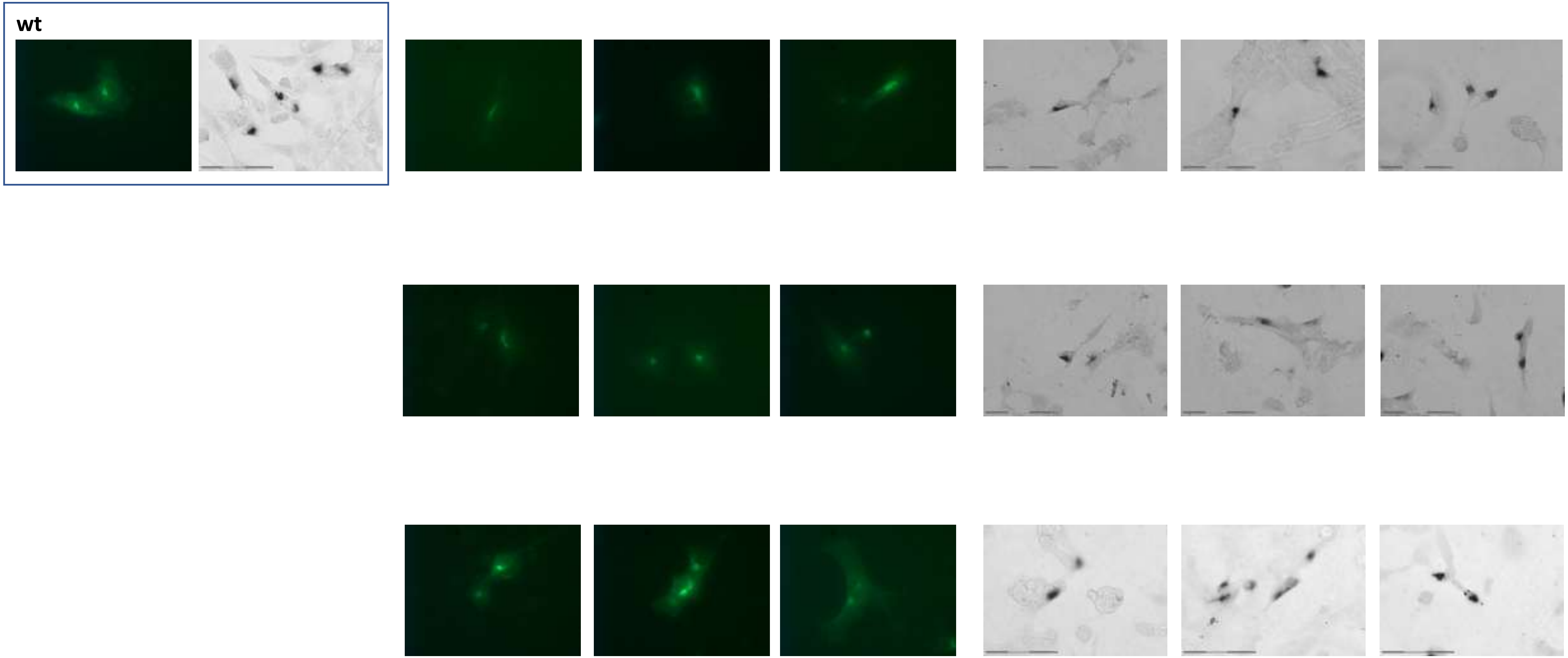

# G943D (TM5,6 loop)

Reduced expression; significant activity

wt

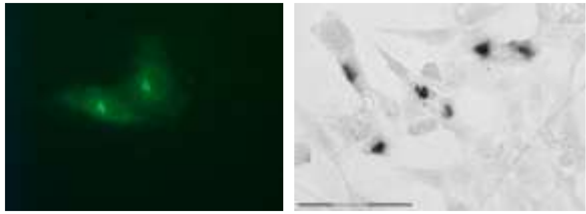

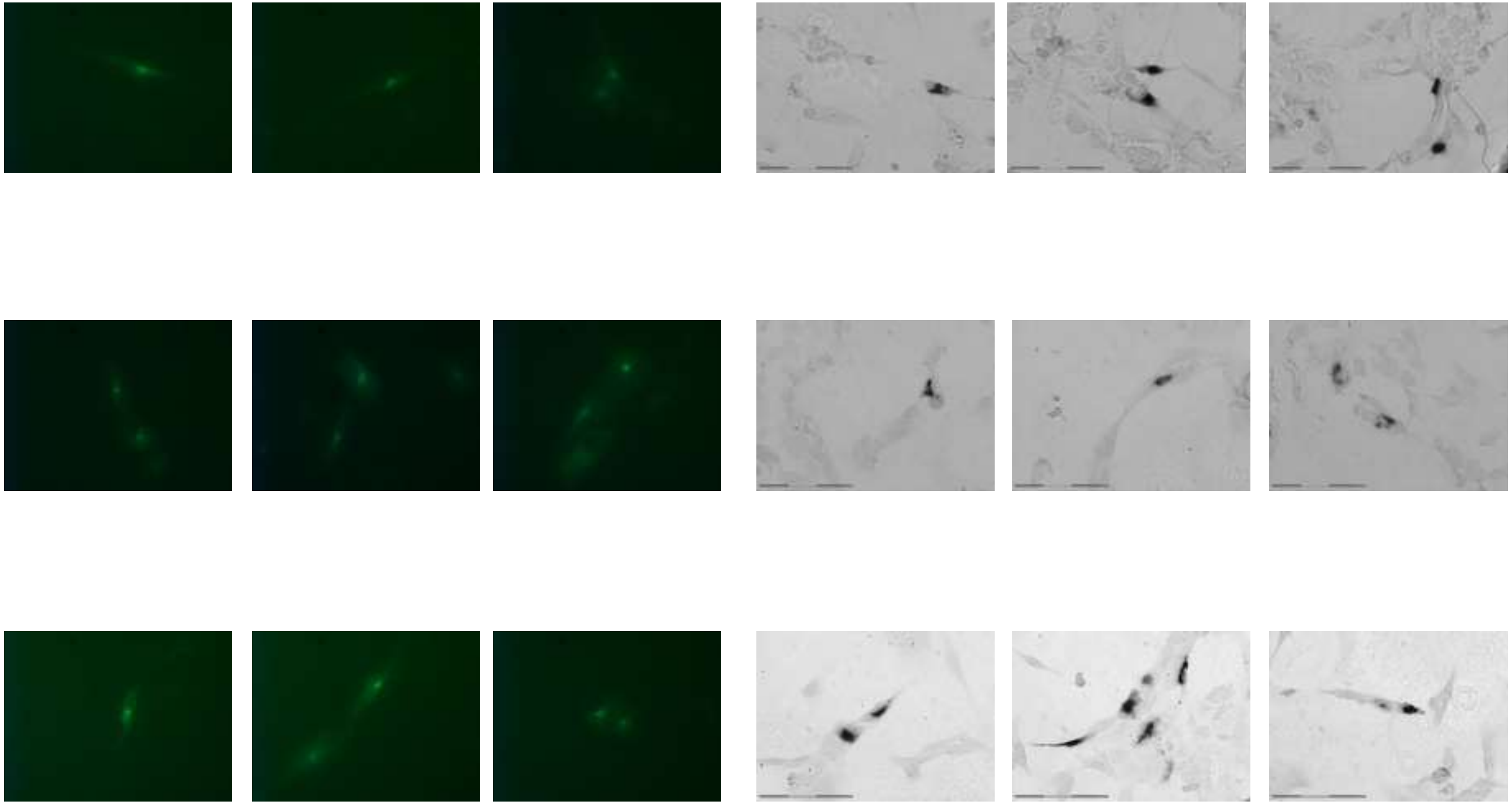

# G943S (TM5,6 loop)

Reduced expression; significant activity

wt

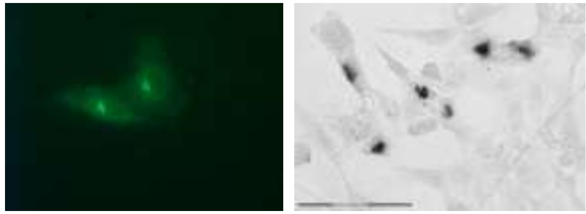

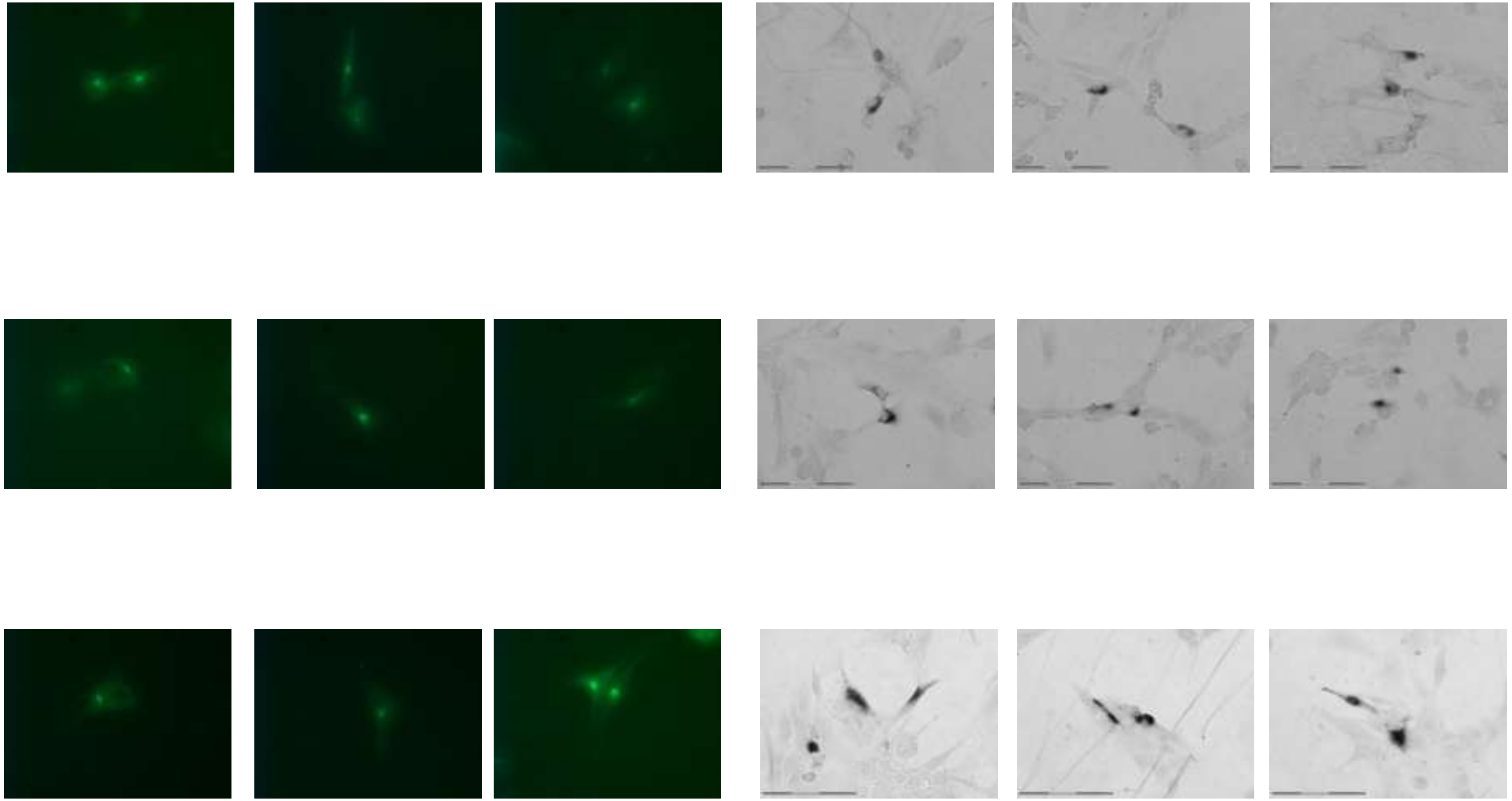

# T935M (TM5,6 loop)

Reduced expression; normal activity

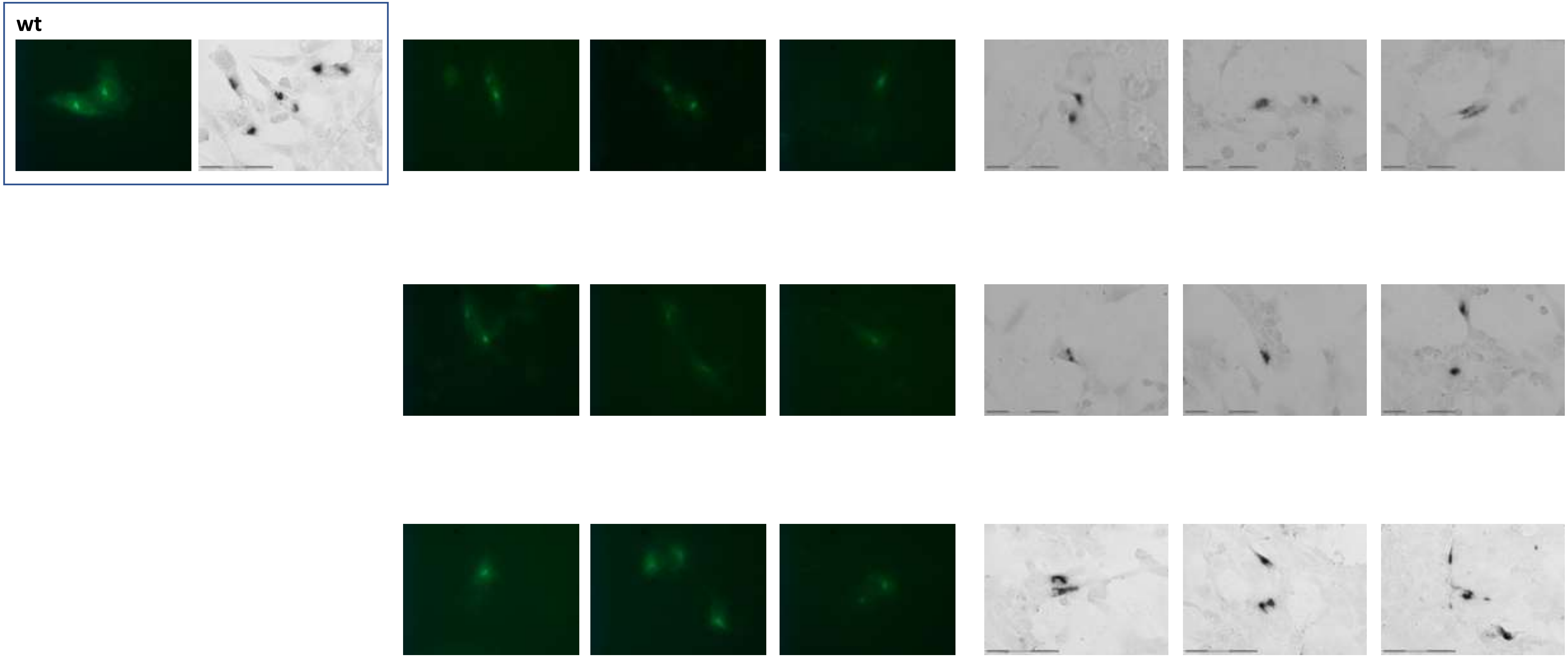

# I929V (TM5)

**Normal expression; reduced activity**

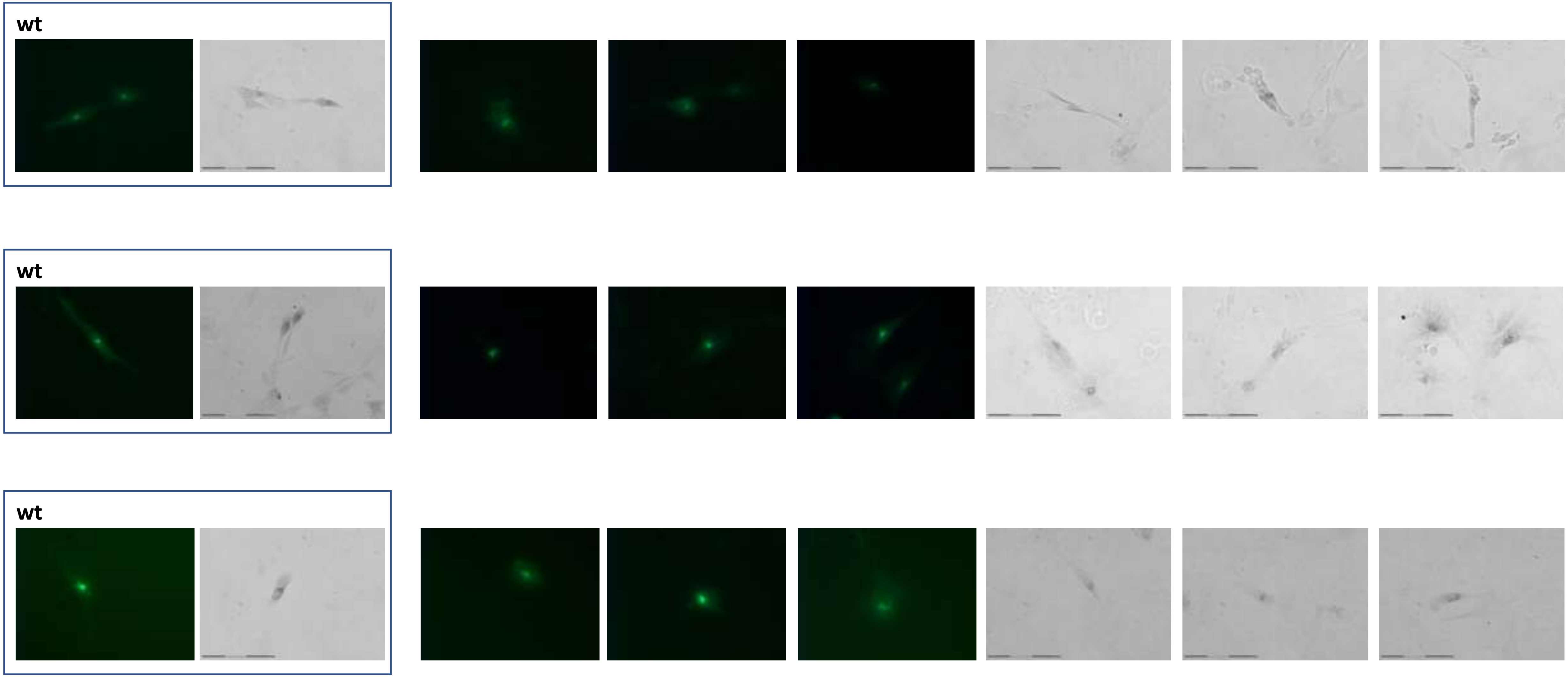

# R919W (A-domain)

Normal expression; significant activity

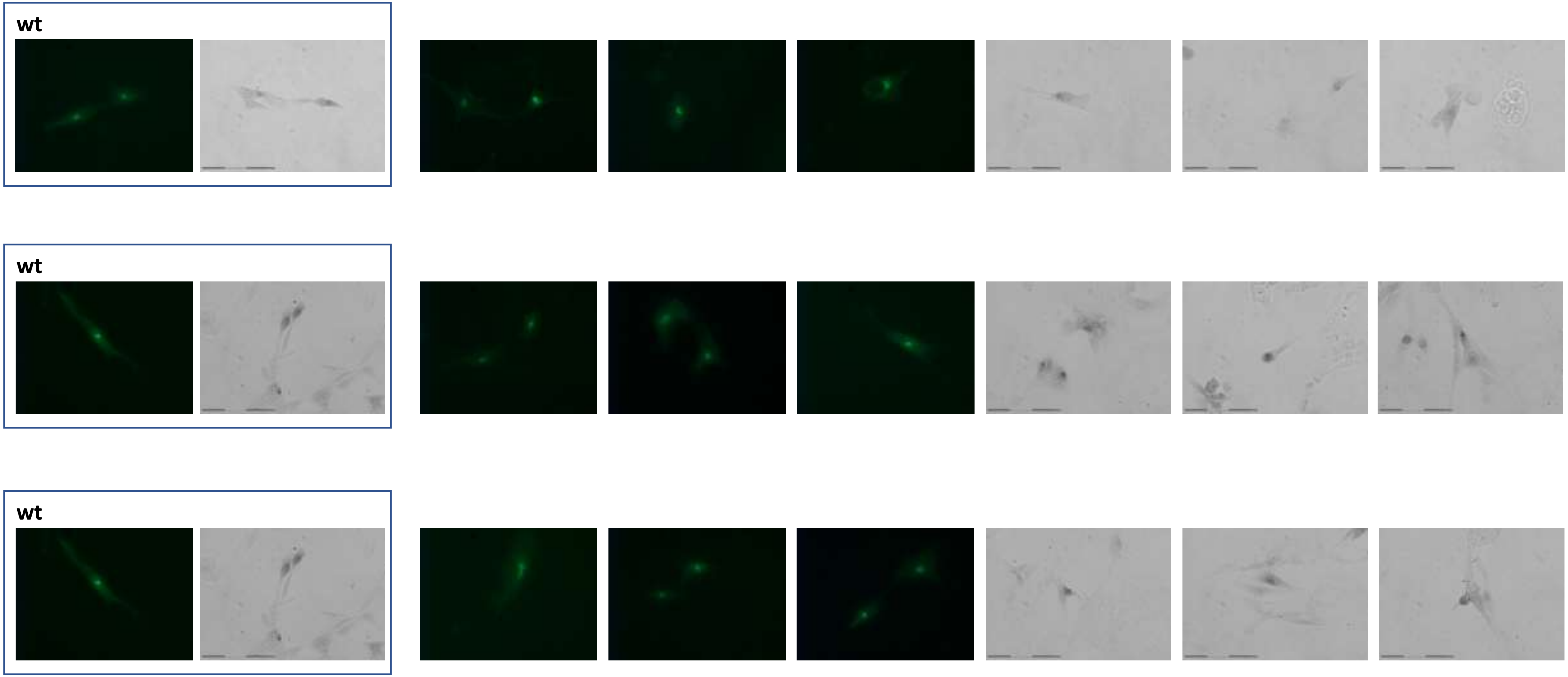

# R919G (A-domain)

Normal expression; significant activity

wt

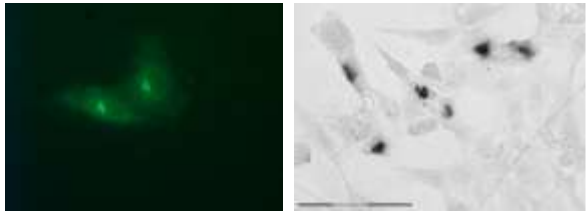

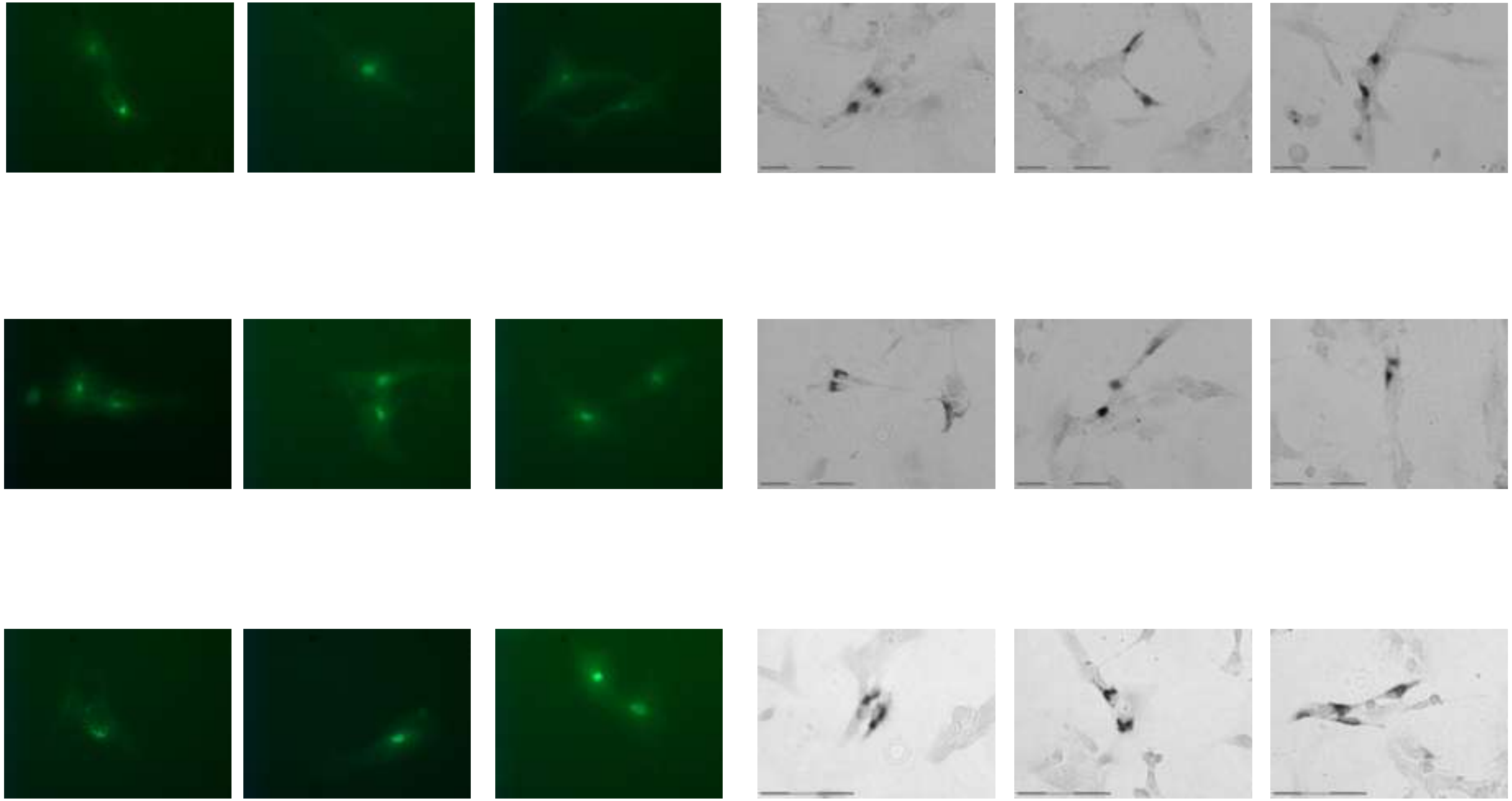

# S876C (A-domain)

**Normal expression; reduced activity**

**wt**

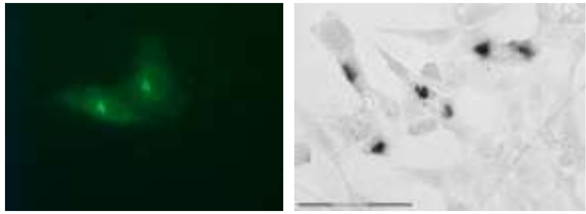

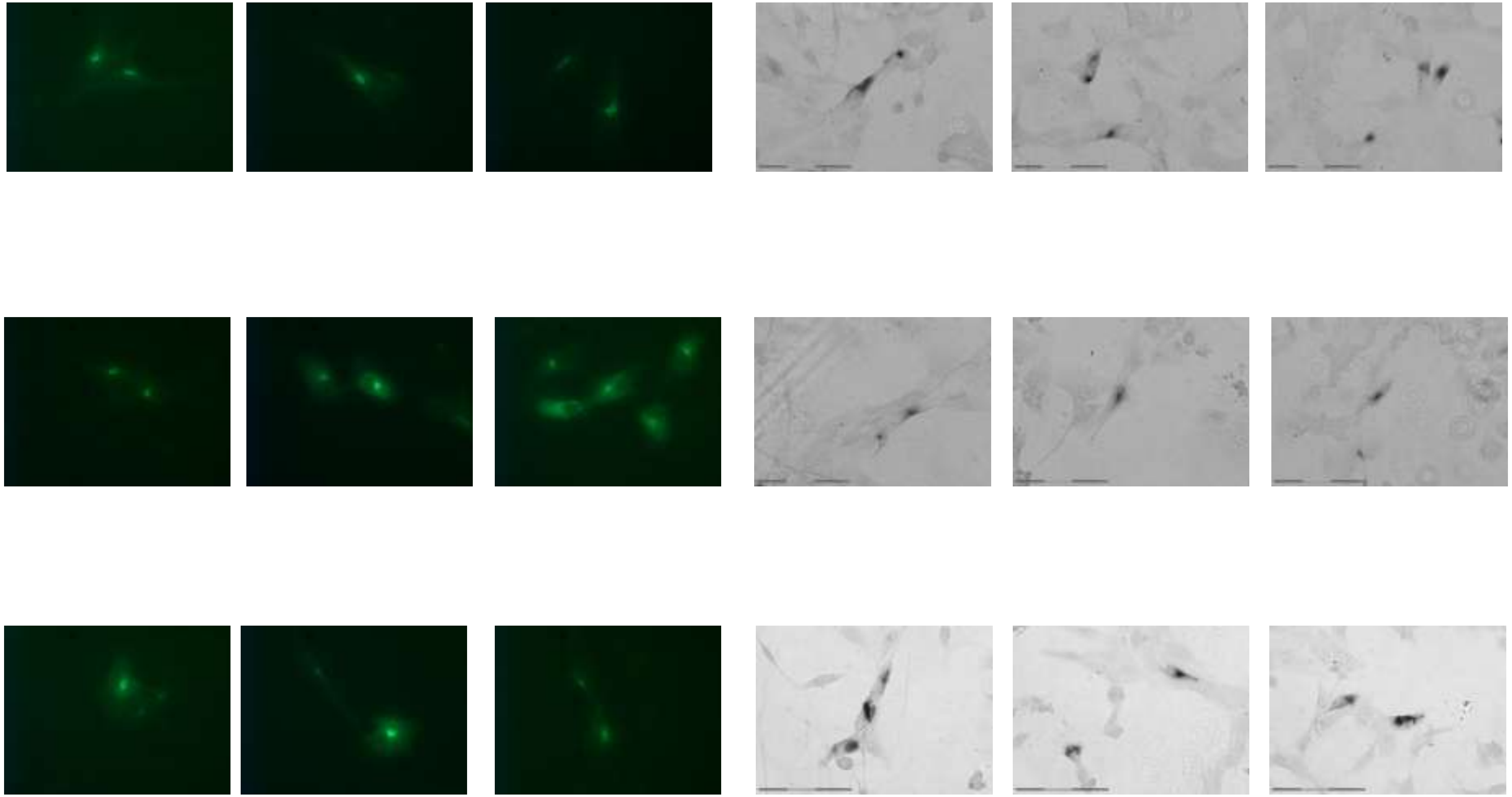

# A874V (A-domain)

**Reduced expression; reduced activity**

**wt**

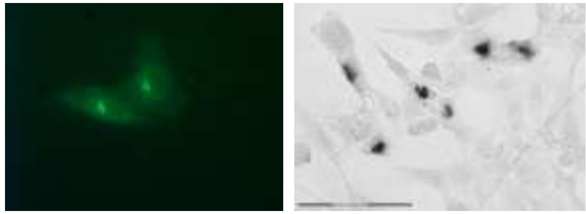

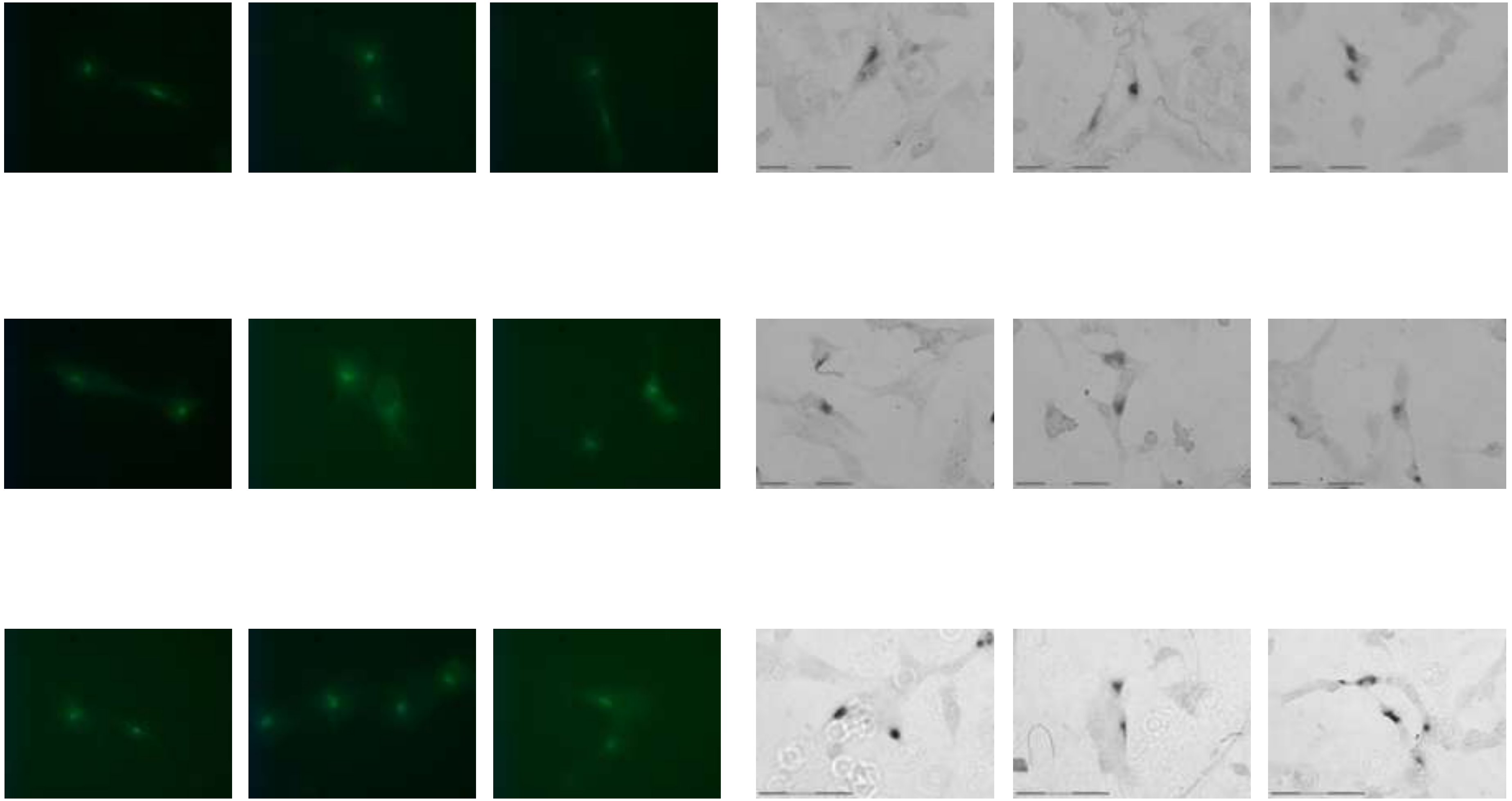

# G869R (A-domain)

**Normal expression; reduced activity**

wt

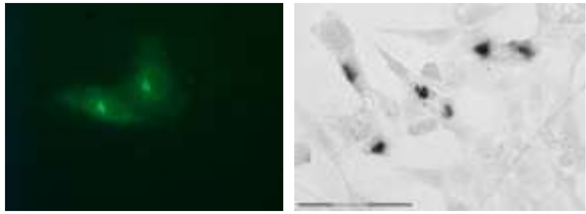

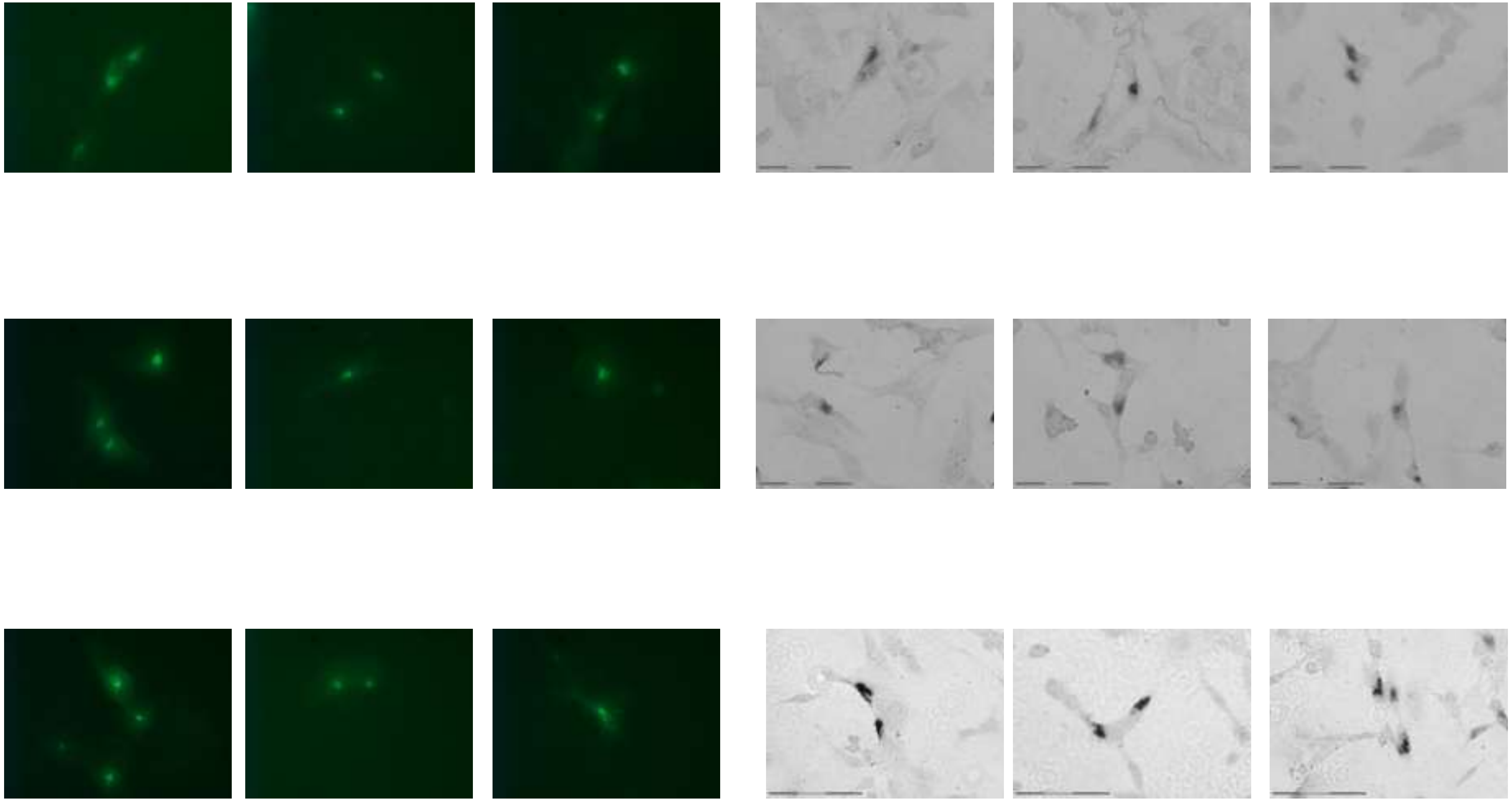

# T850I (A-domain)

Normal expression; significant activity

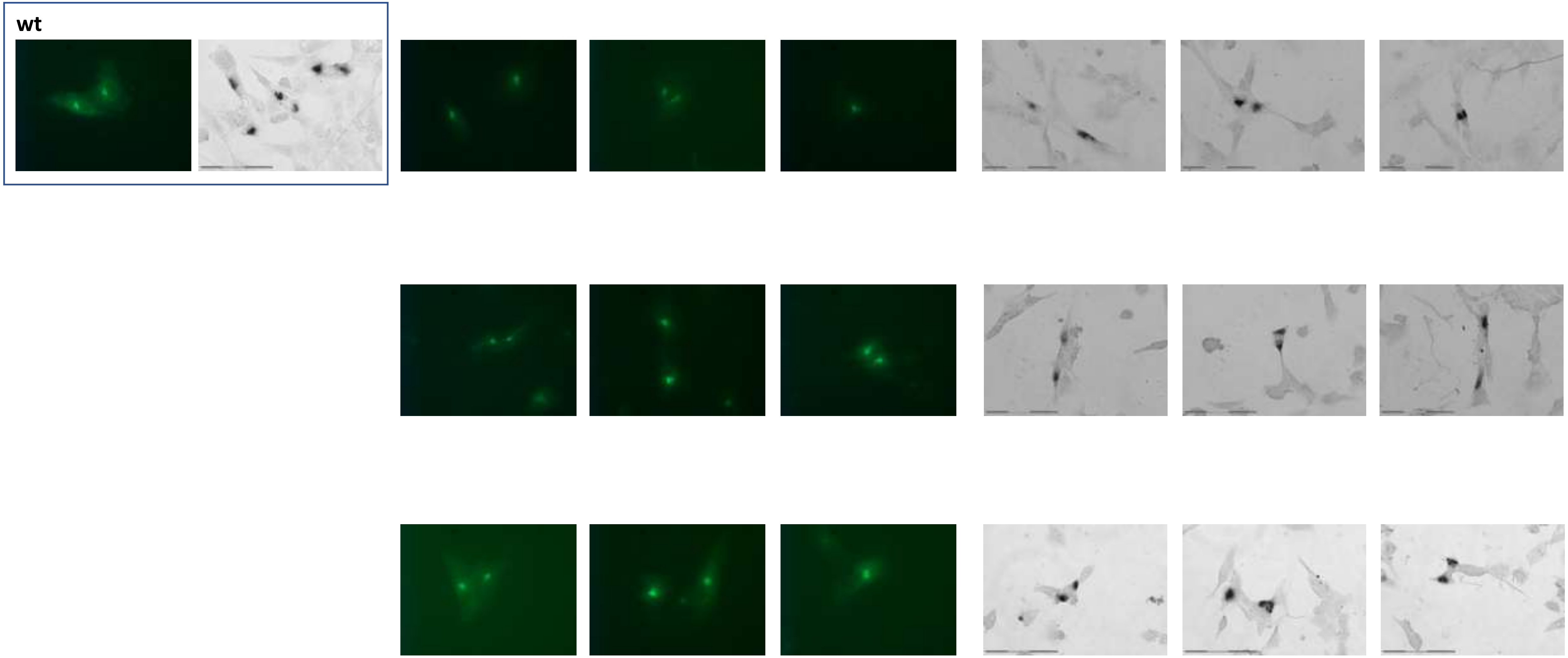

# K832R (A-domain)

Normal expression; significant activity

**wt**

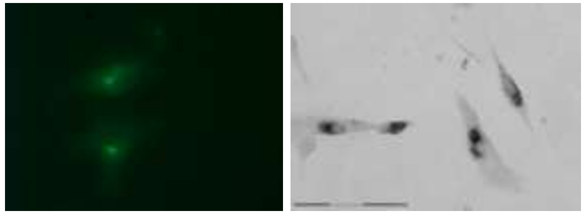

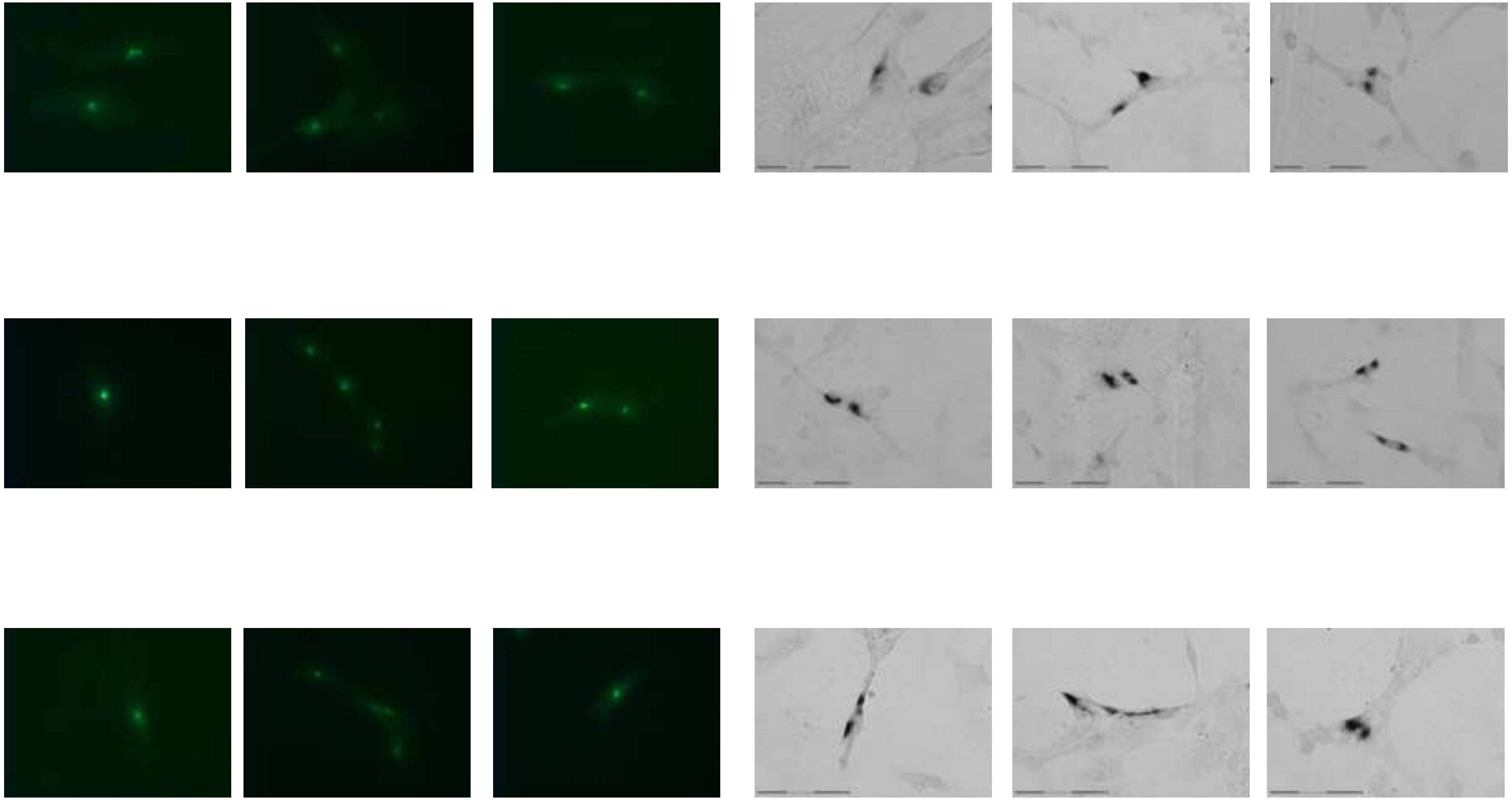

# R827W (A-domain)

**Normal expression; significant activity**

**wt**

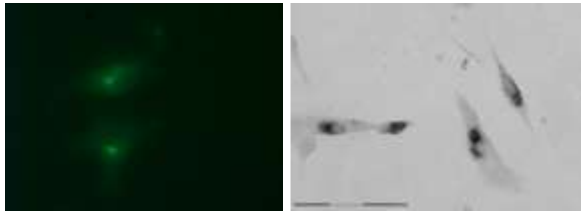

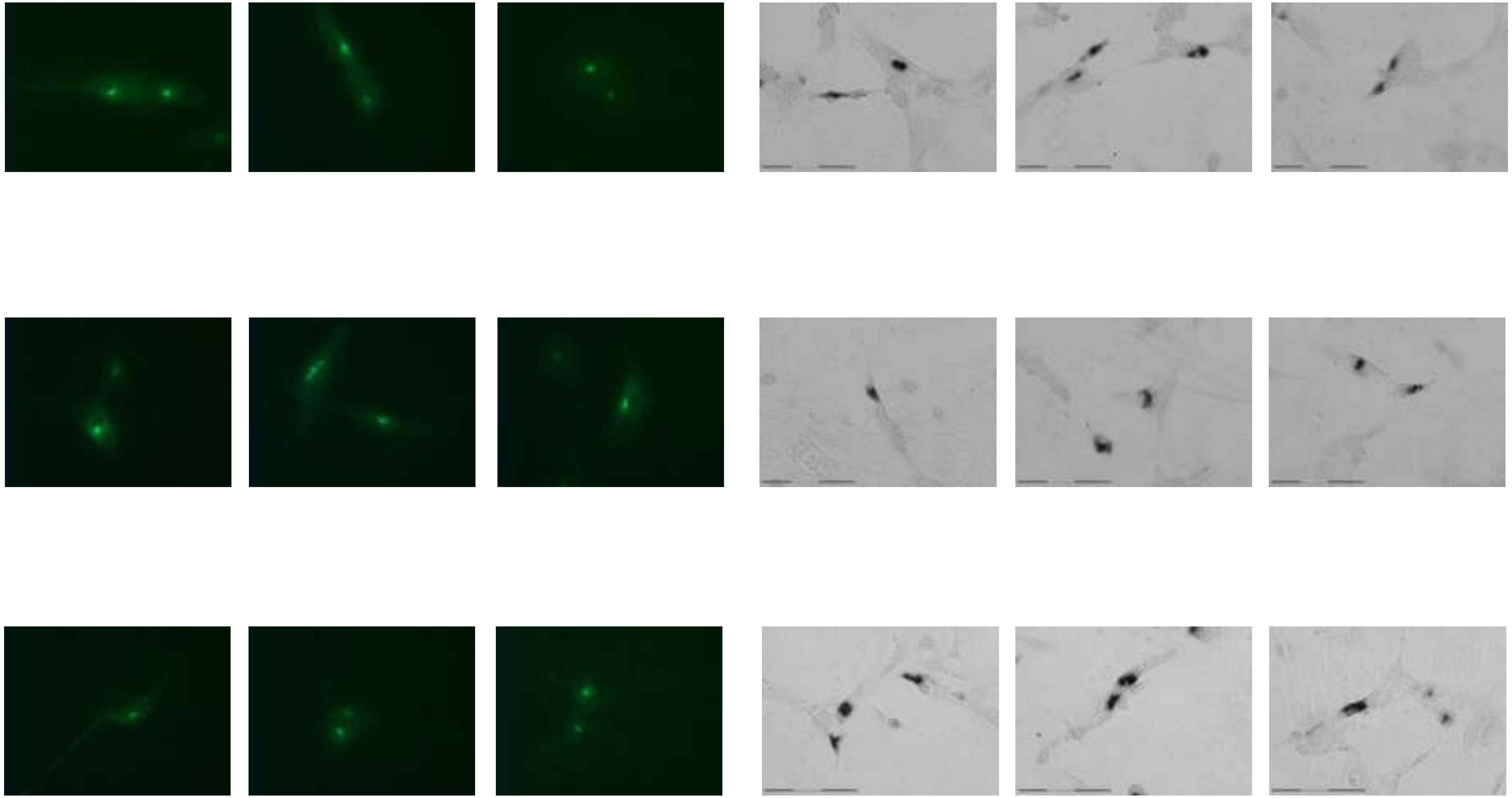

# R778L (TM4)

**Reduced expression; reduced activity**

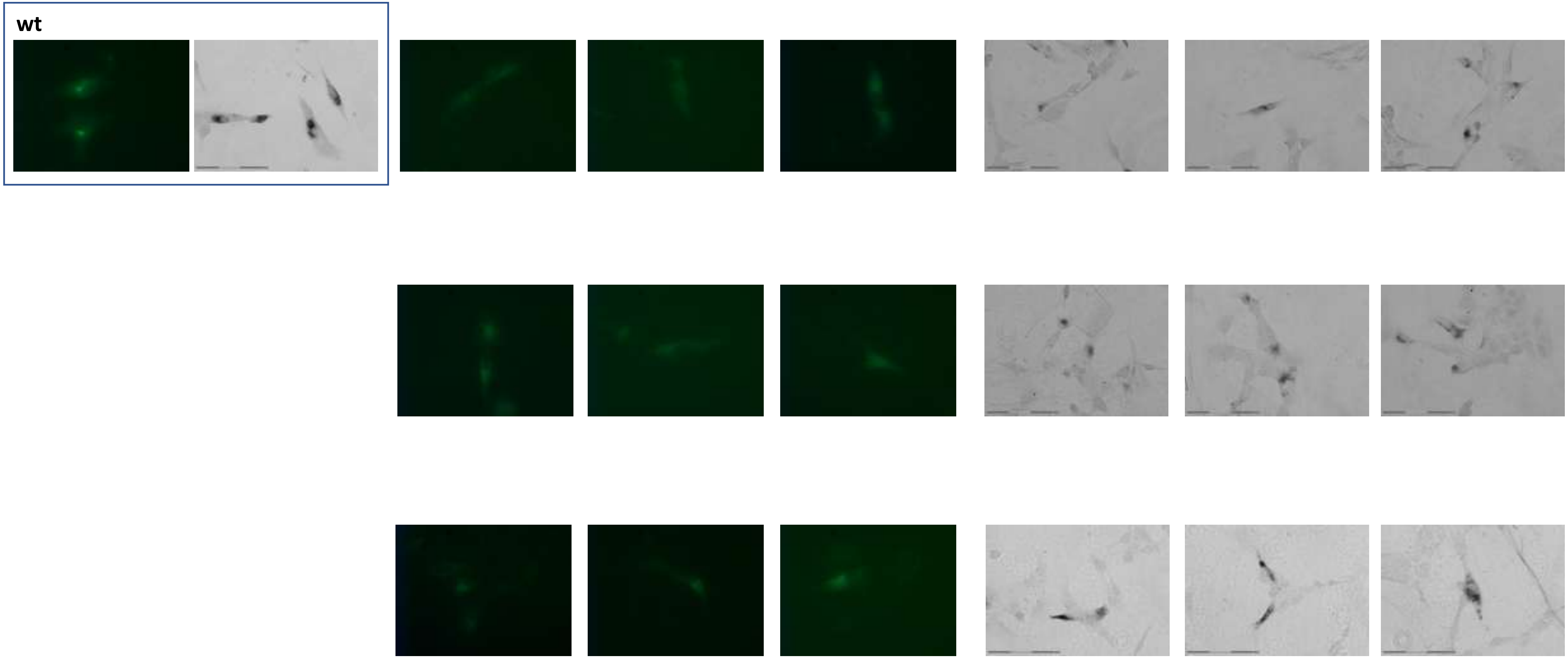

# R778Q (TM4)

**Reduced expression; reduced activity**

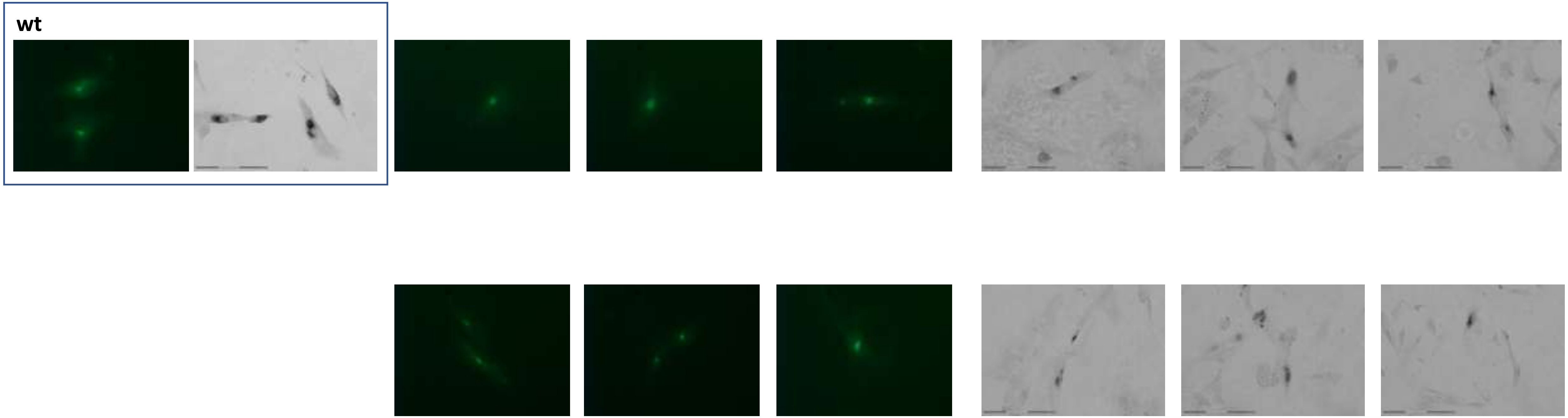

# R778W (TM4)

**Reduced expression; reduced activity**

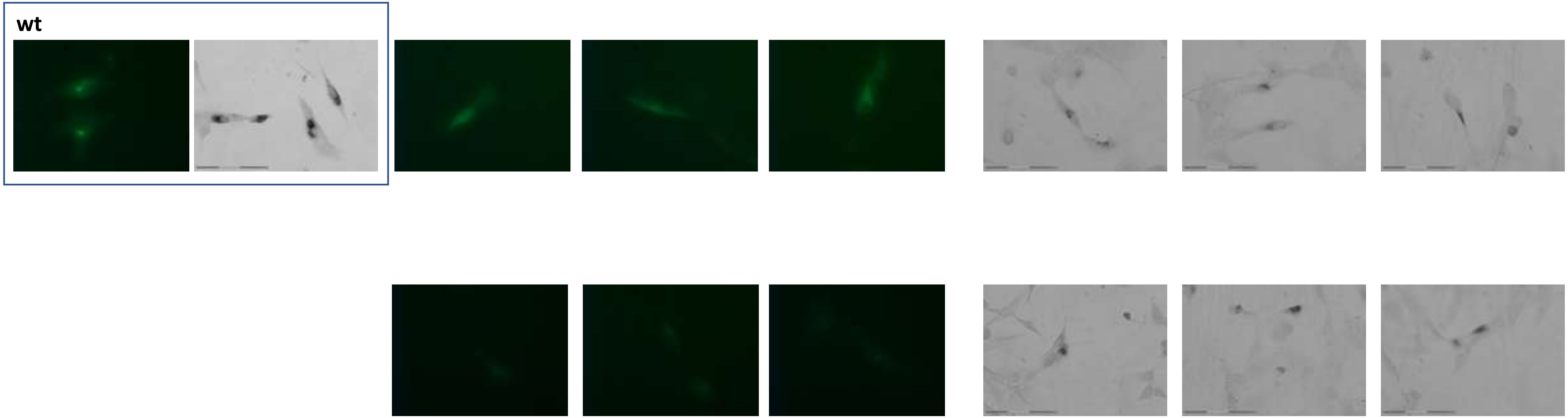

# M769V (TM5)

Normal expression; reduced activity

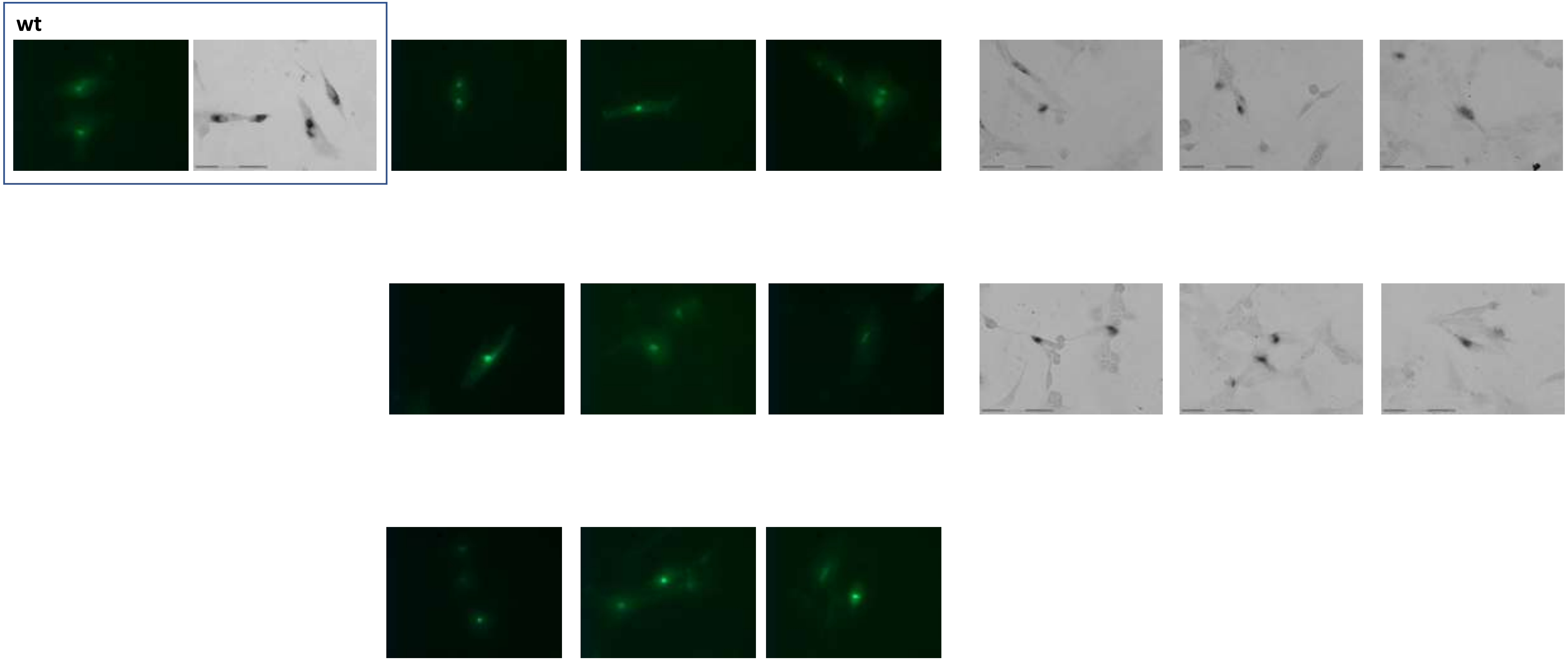

# V731M (TM3)

**Normal expression; reduced activity**

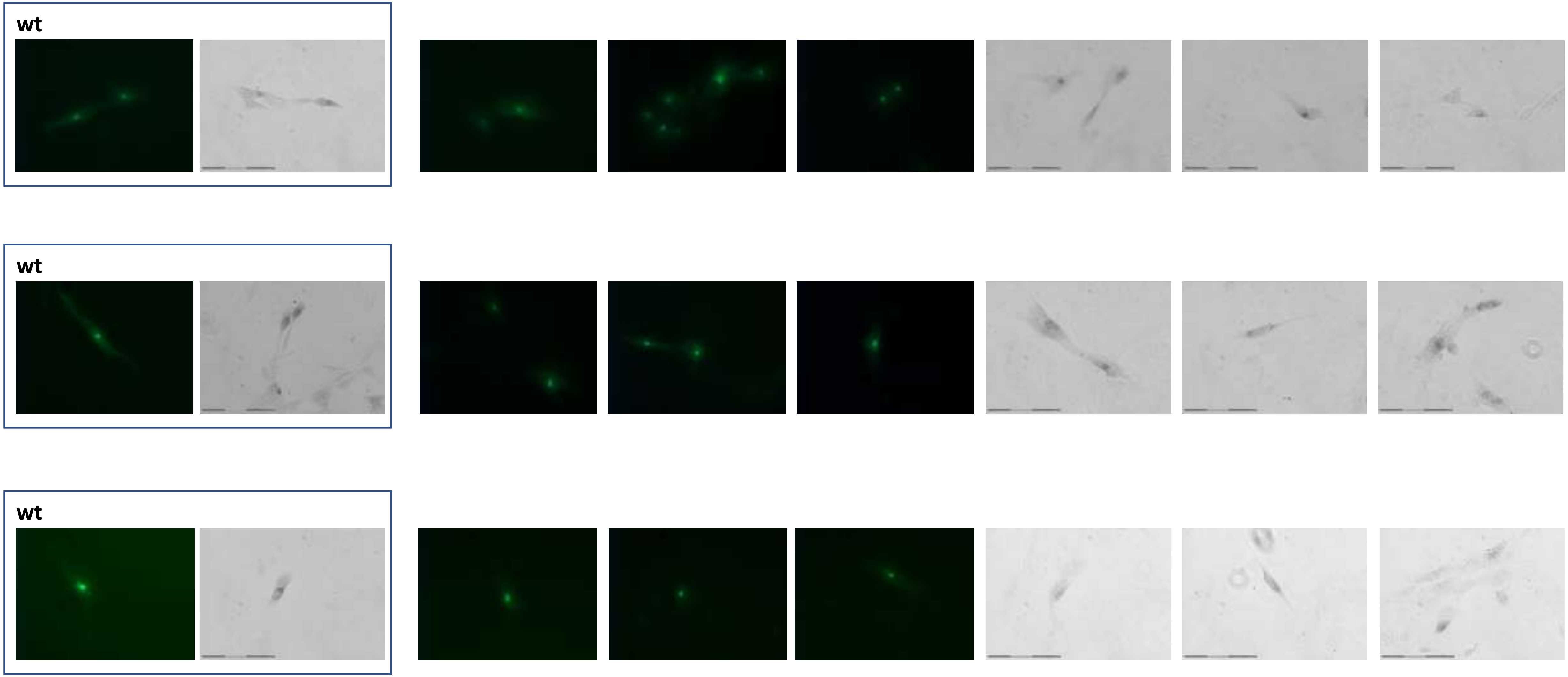

# N728S (TM3)

**Normal expression; reduced activity**

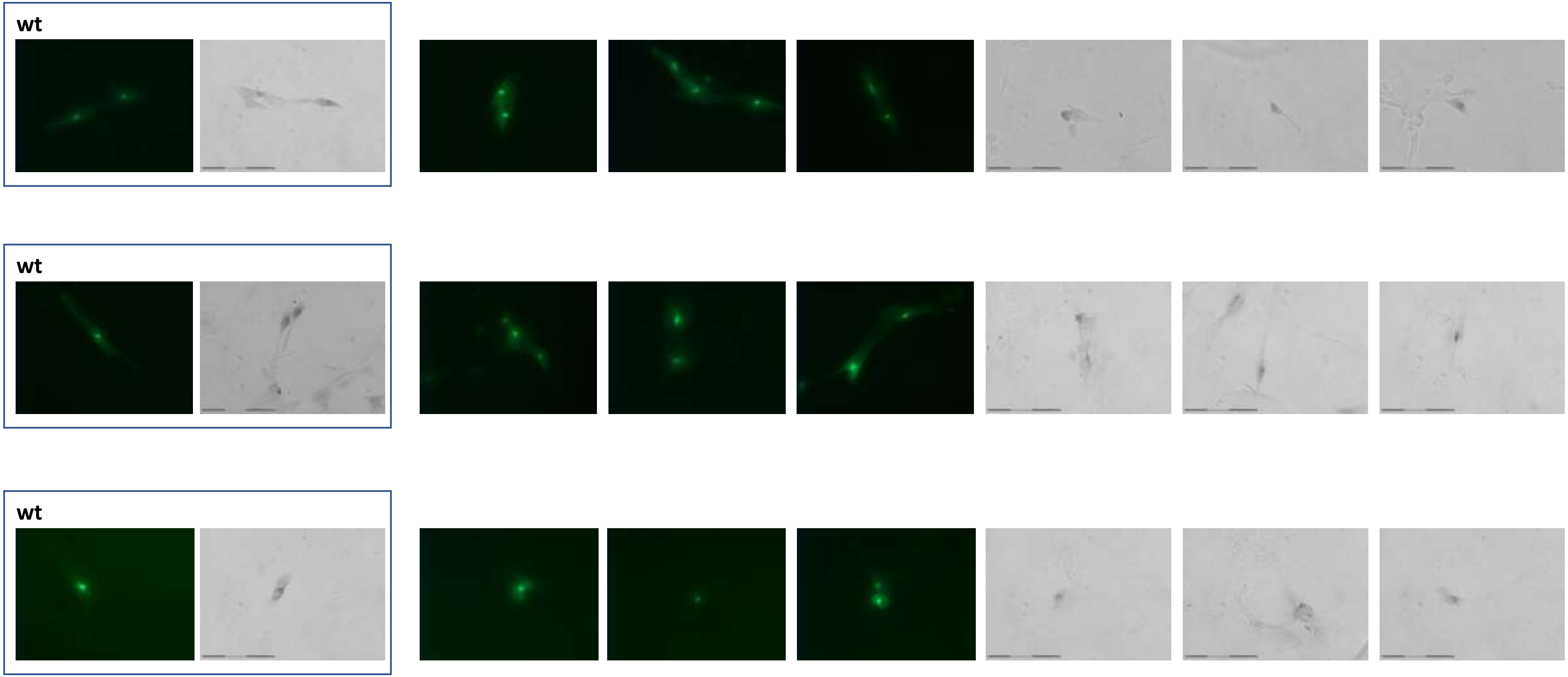

# Y670C (TM1)

**Normal expression; reduced activity**

**wt**

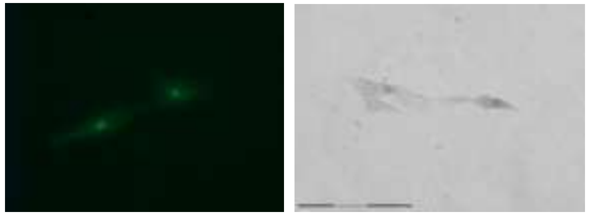

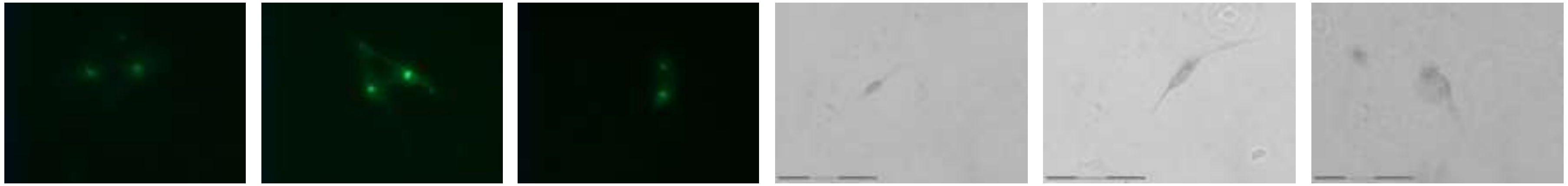

**wt**

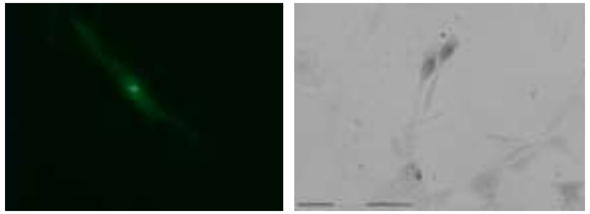

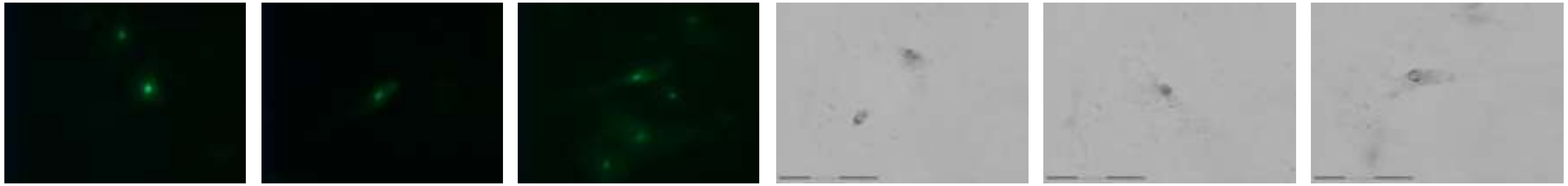

**wt**

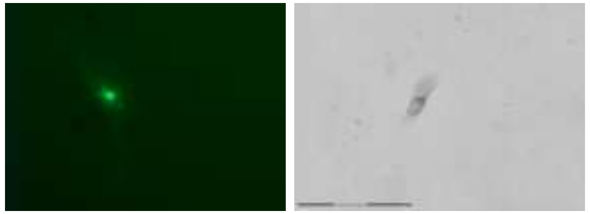

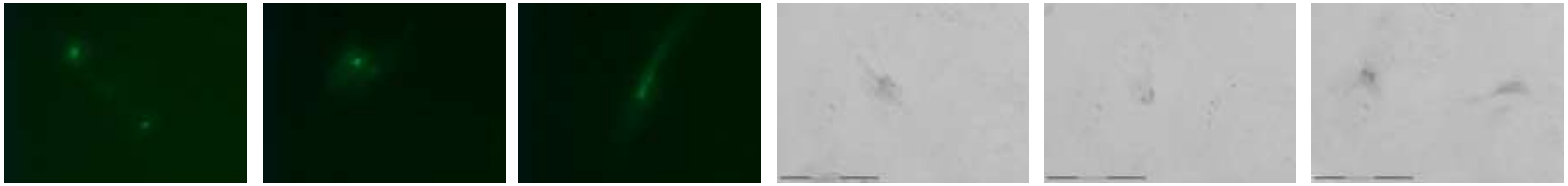

# M668V (TM1)

Normal expression; reduced activity

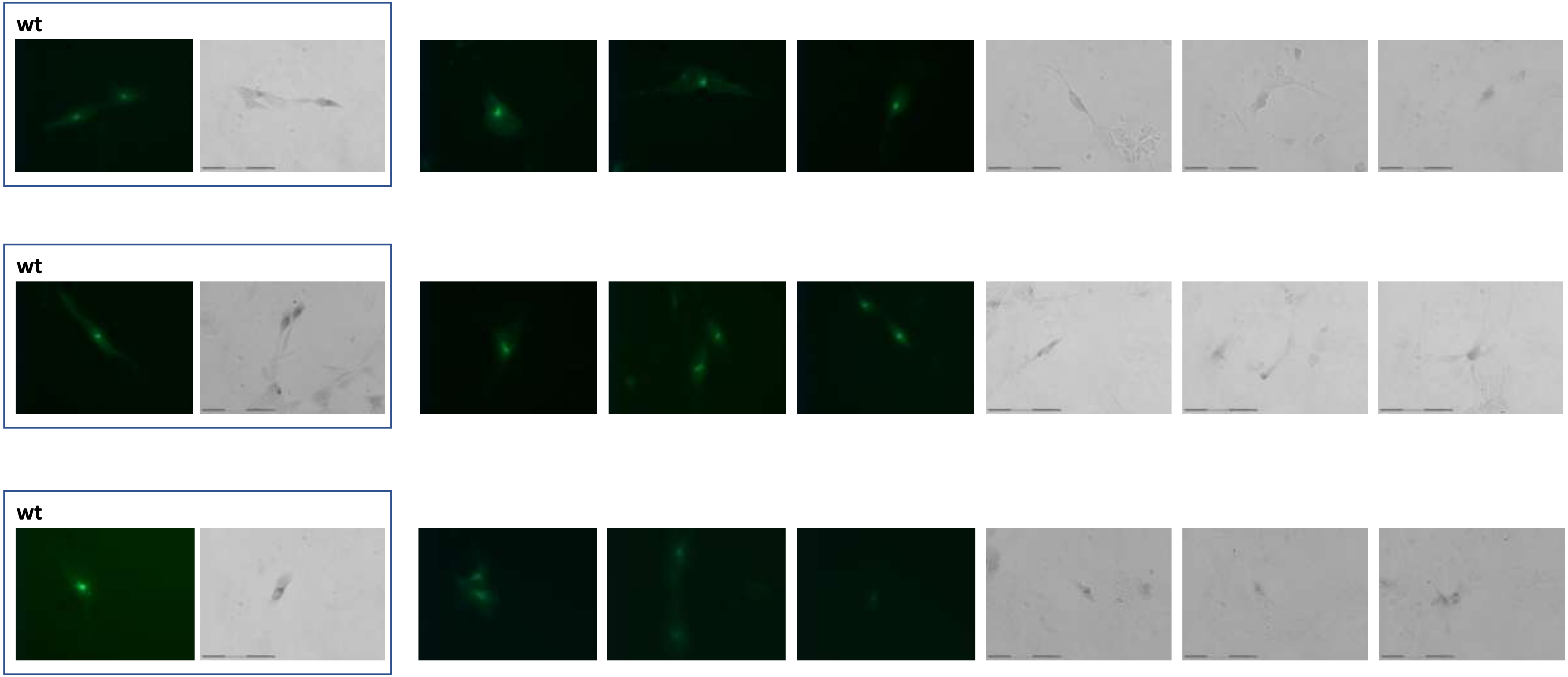

# M665I (TM1)

Normal expression; reduced activity

**wt**

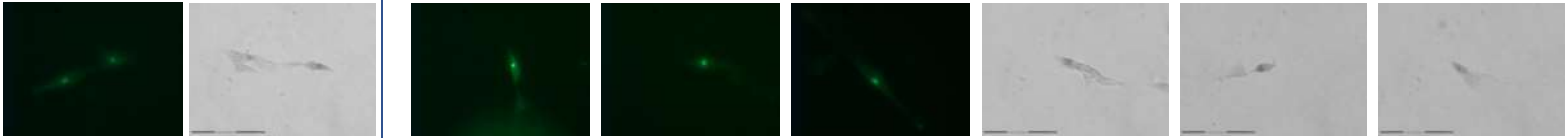

**wt**

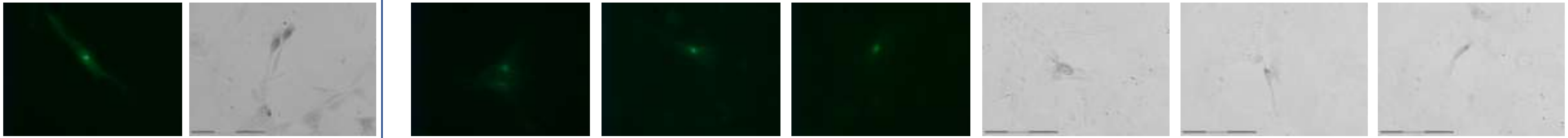

**wt**

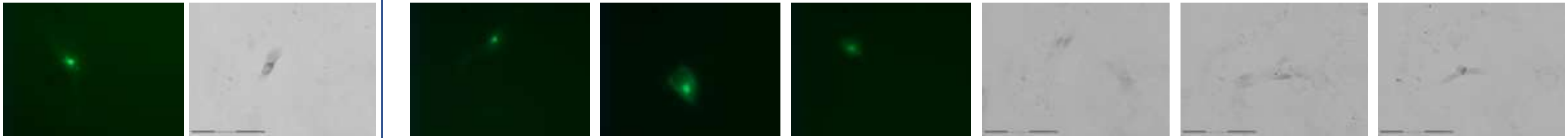

# M665I (TM1)

**Normal expression; no activity**

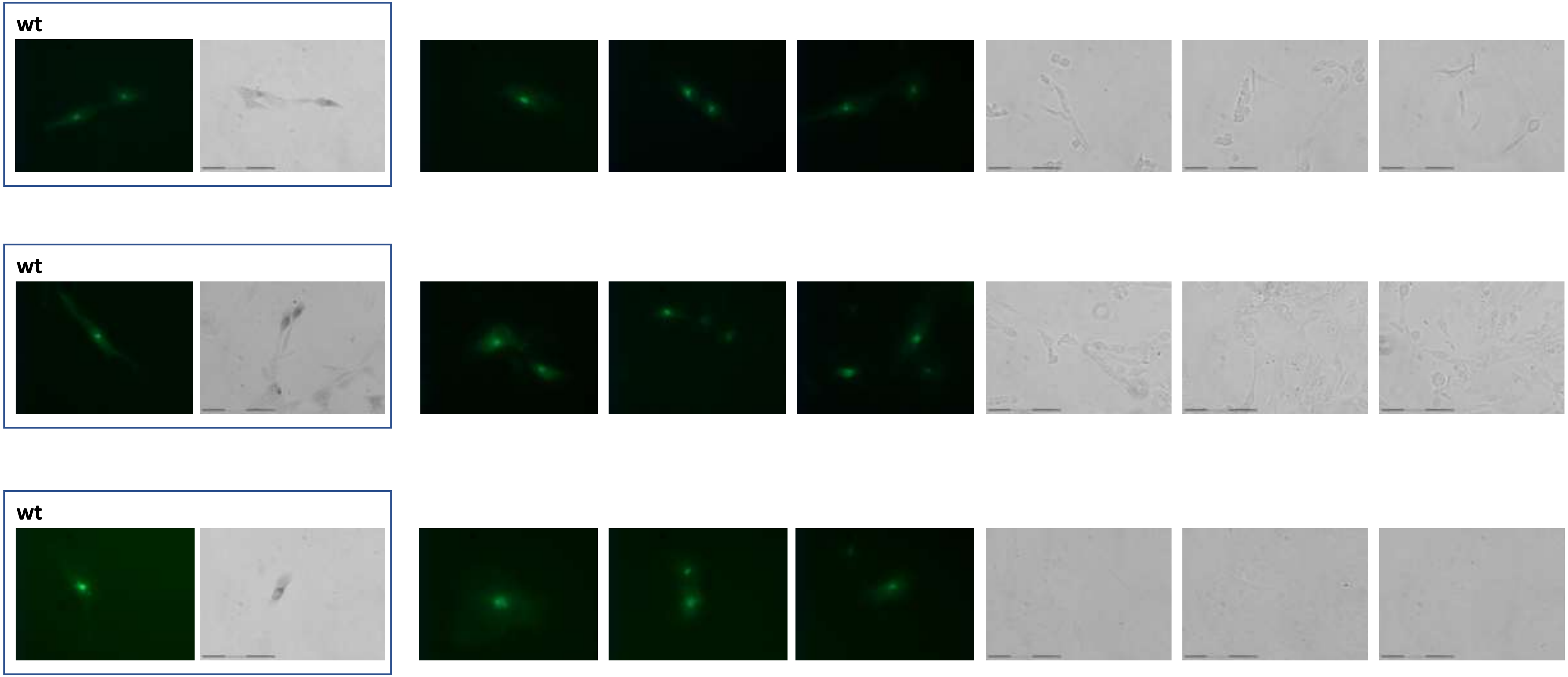

# S657R (TM1)

Reduced expression; no activity

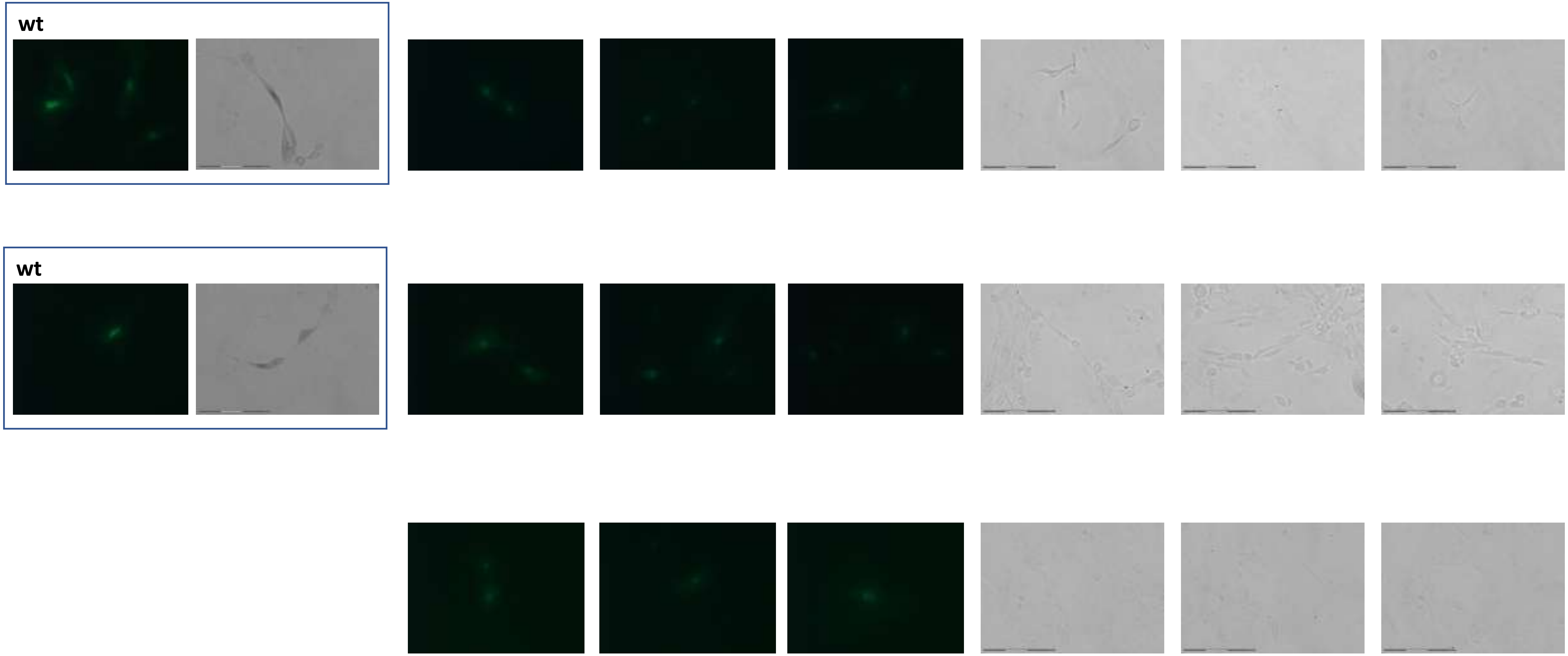

# M645R (MBD6,TM1 linker)

**Normal expression; significant activity**

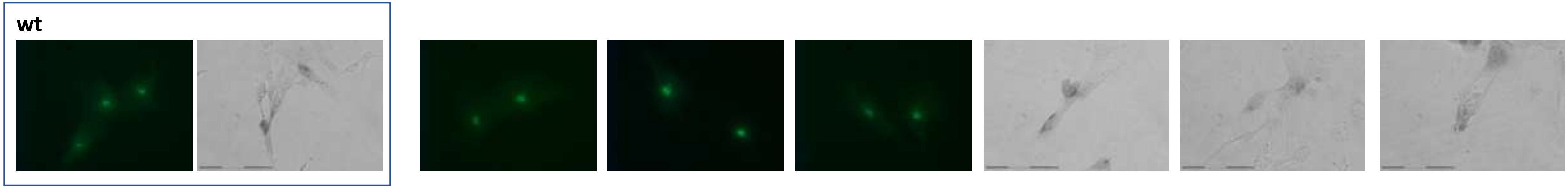


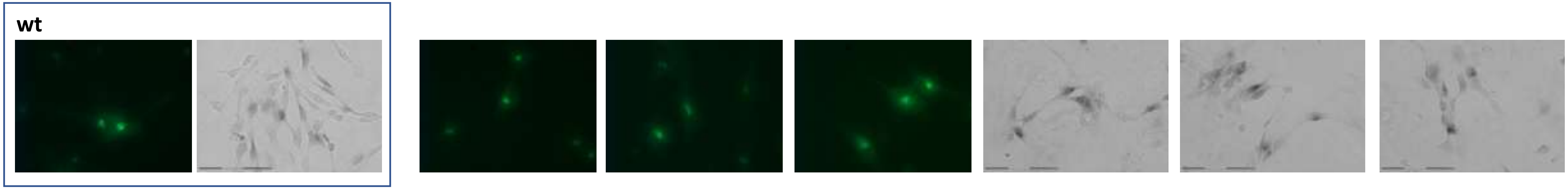

# L641S (MBD6,TM1 linker)

Normal expression; significant activity

wt

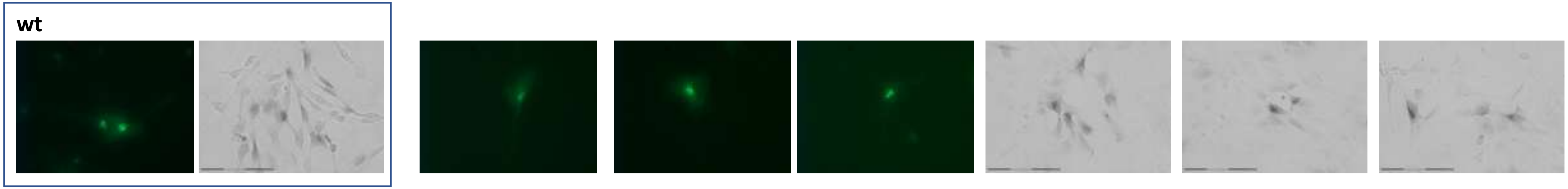

wt

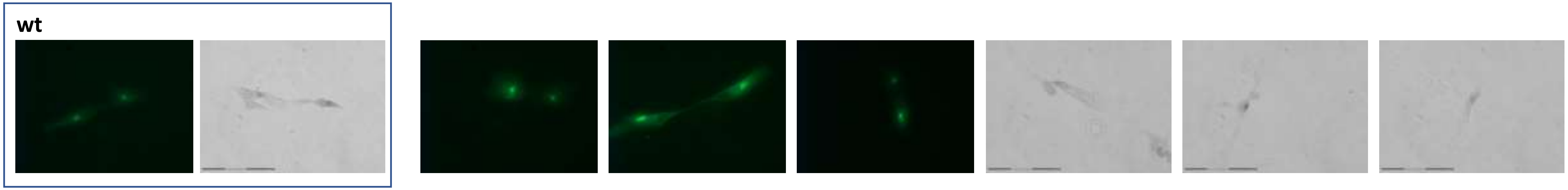

wt

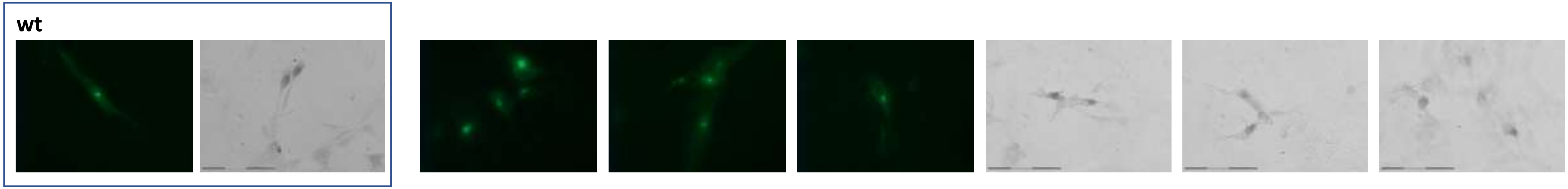

# H639Y (MBD6,TM1 linker)

**Normal expression; significant activity**

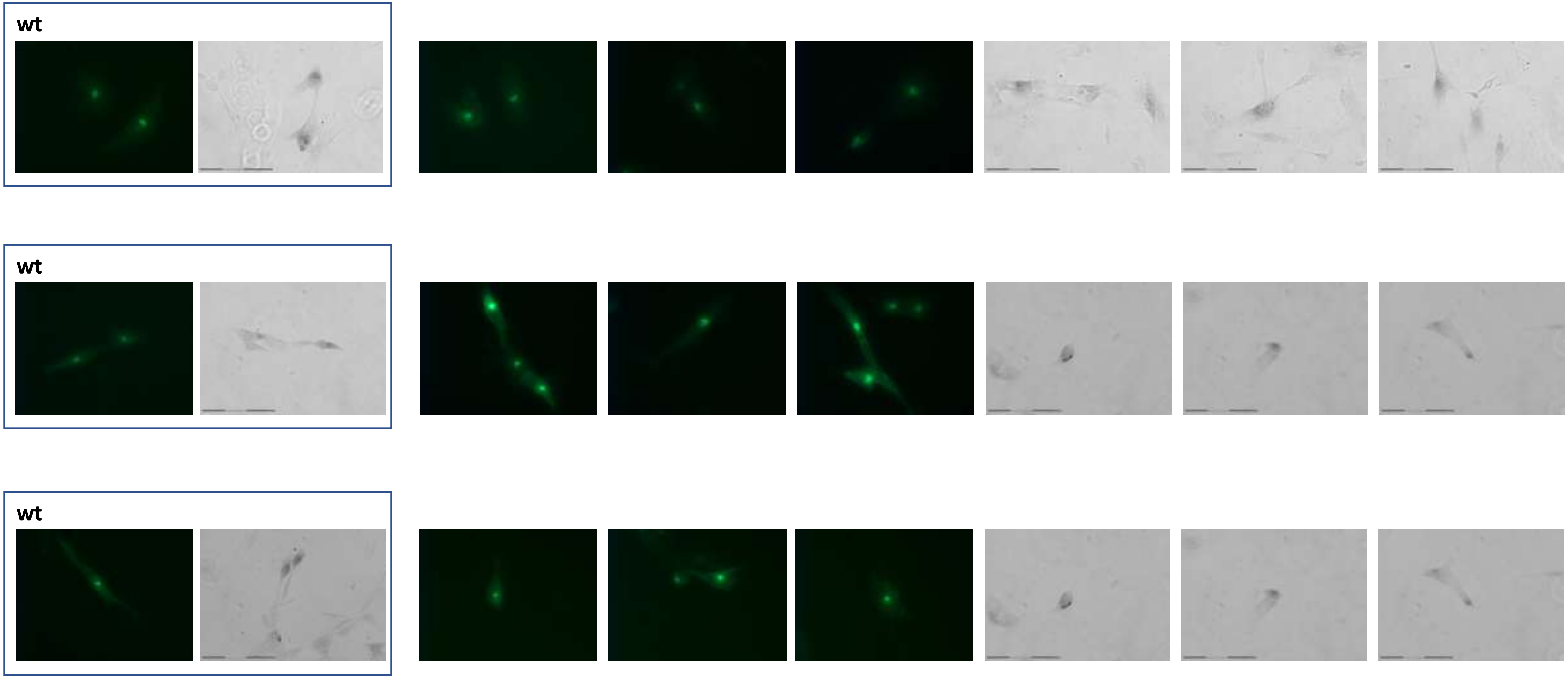

# G626A (MBD6)

**Reduced expression; reduced activity**

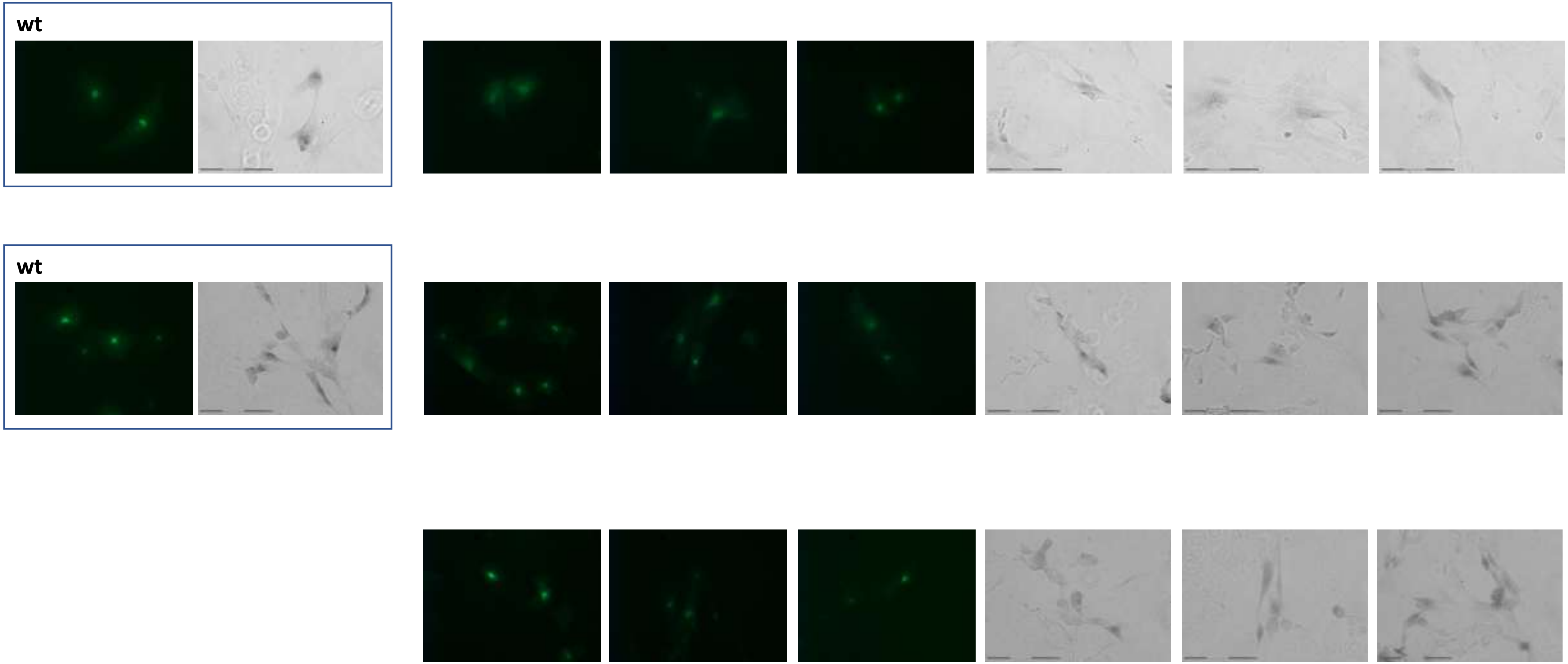

# G641S (MBD6)

**Normal expression; reduced activity**

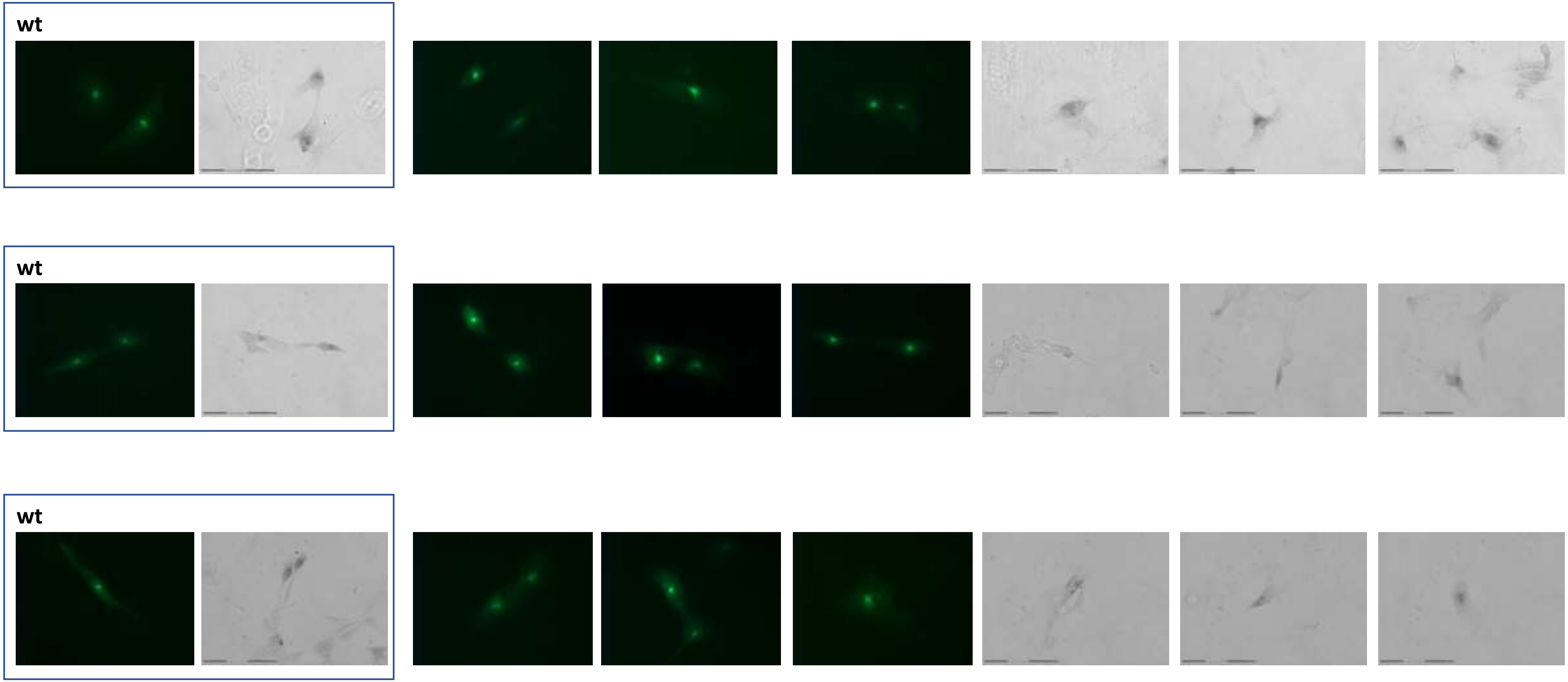

# 159: P610L (MBD6)

**Reduced expression; reduced activity**

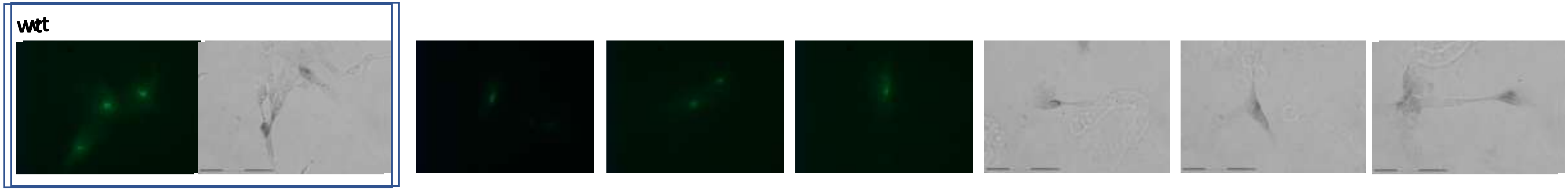


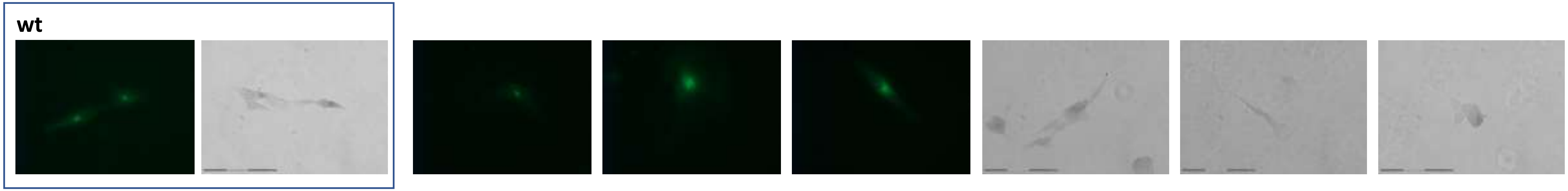

# V536A (MBD5)

Normal expression; reduced activity

wt

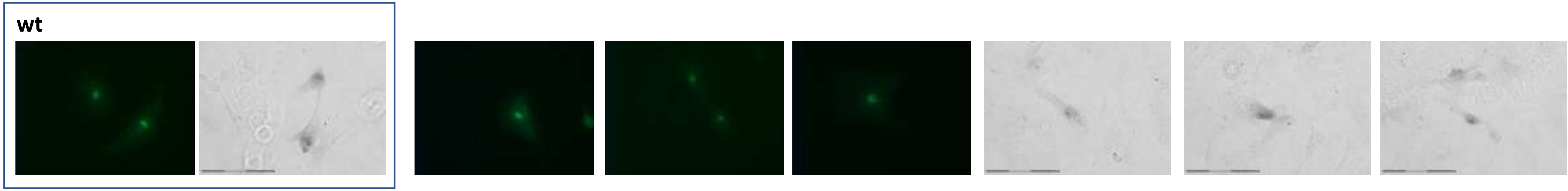

T2 (02/17/22)

wt

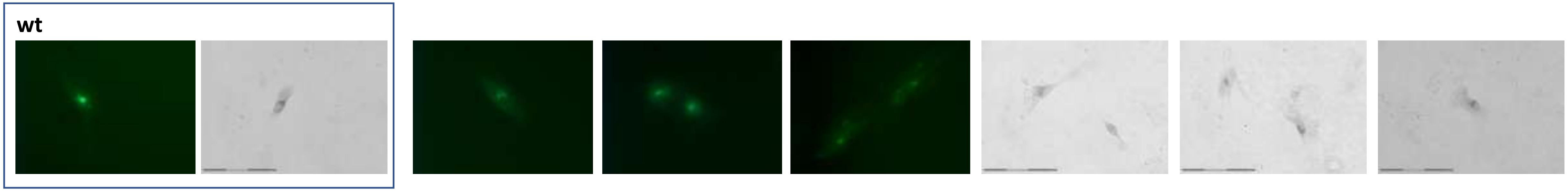

# V519M (MBD5)

Normal expression; reduced activity

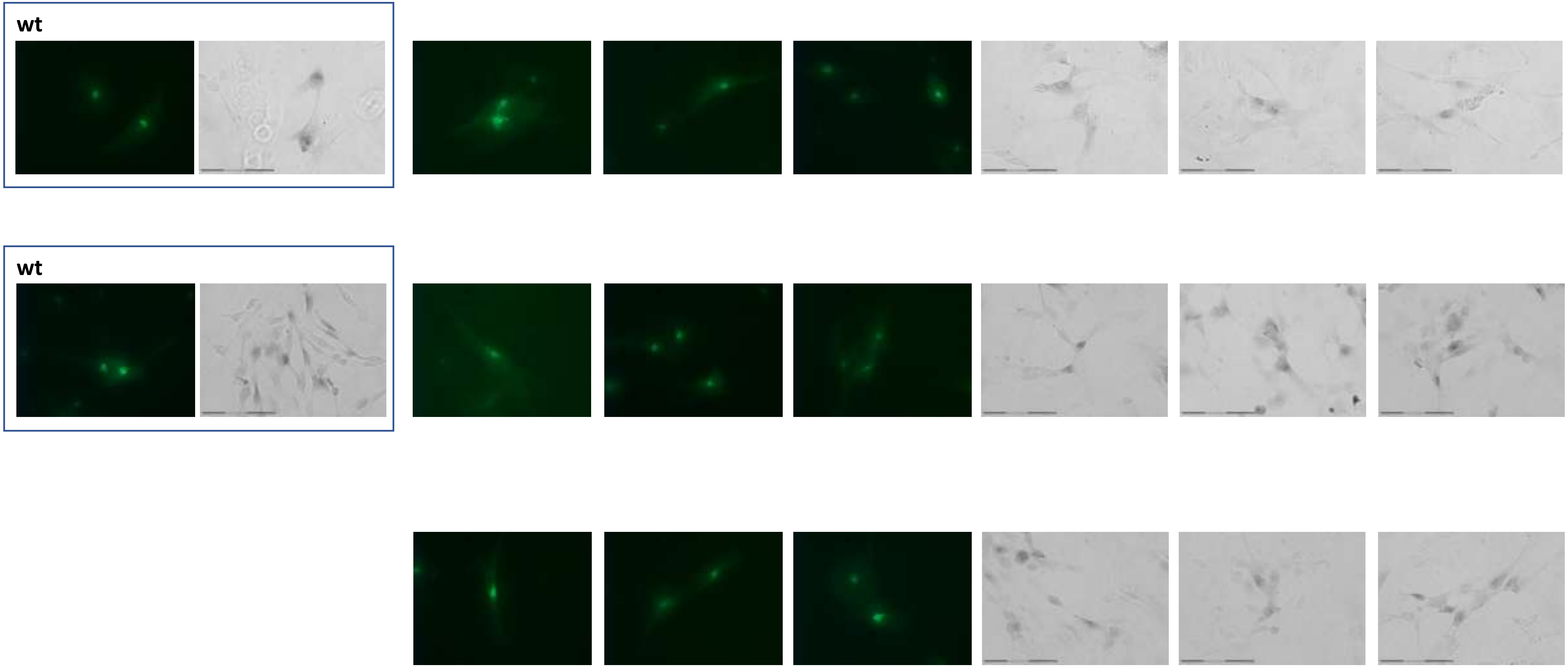

# R508T (MBD5)

**Reduced expression; reduced activity**

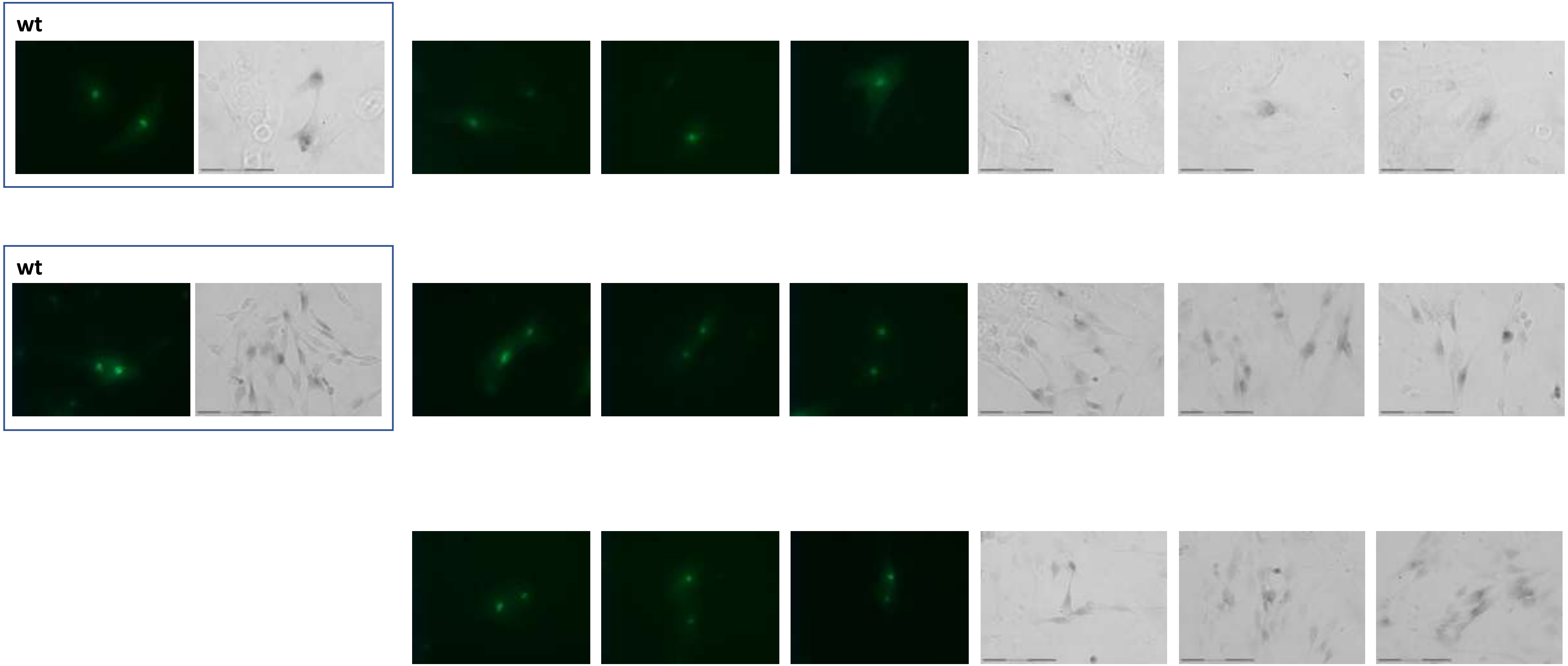

# S501F (MBD5)

Normal expression; reduced activity

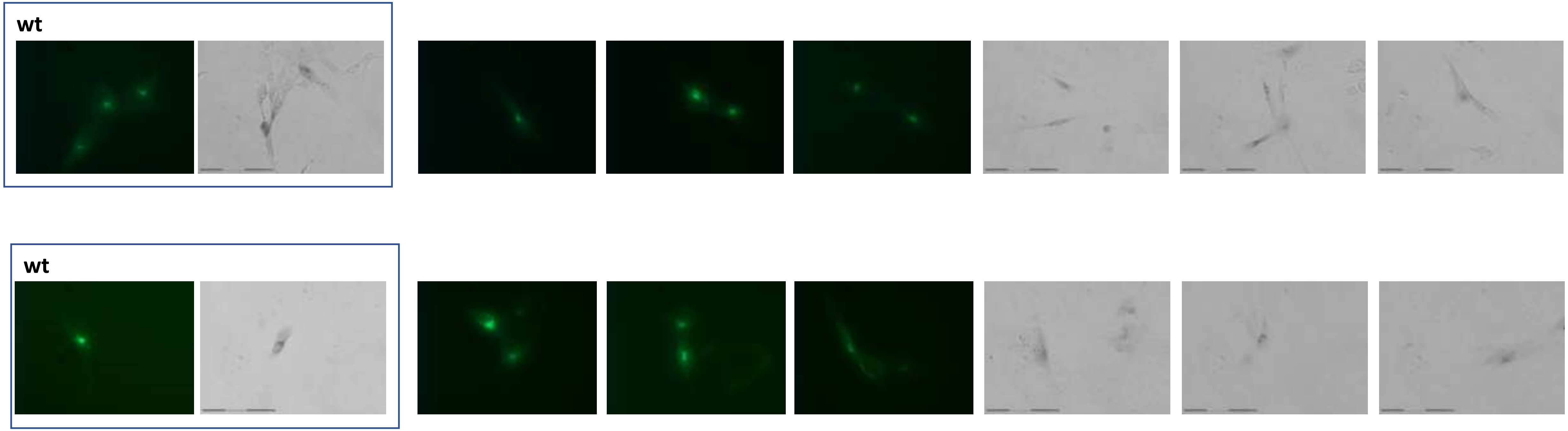

# A476T (MBD4,5 linker)

Normal expression; significant activity

**wt**

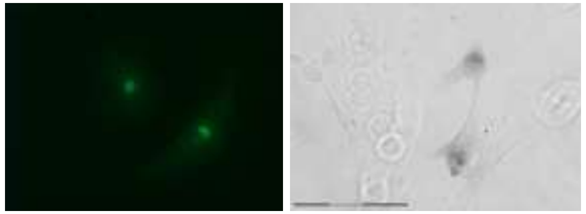

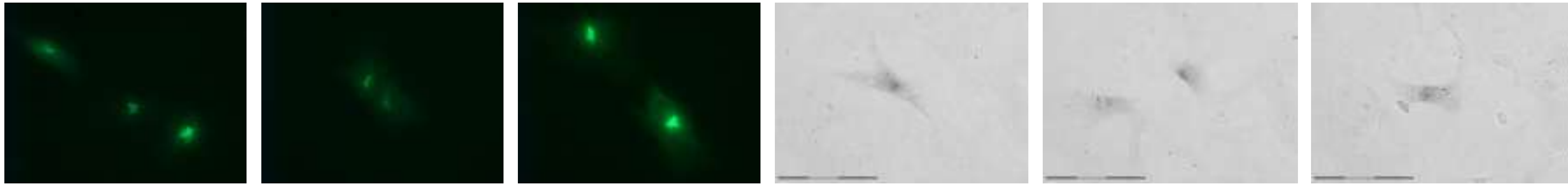

**wt**

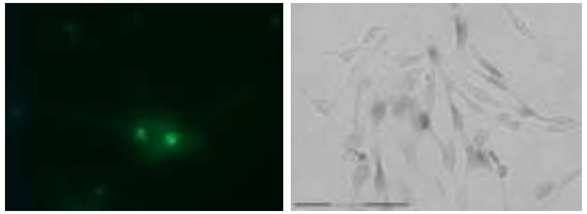

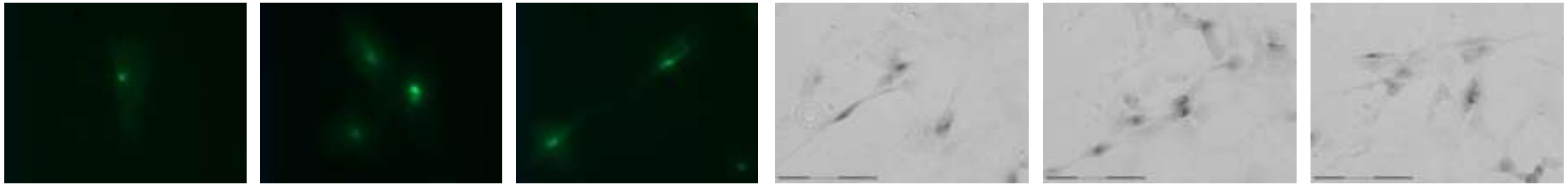

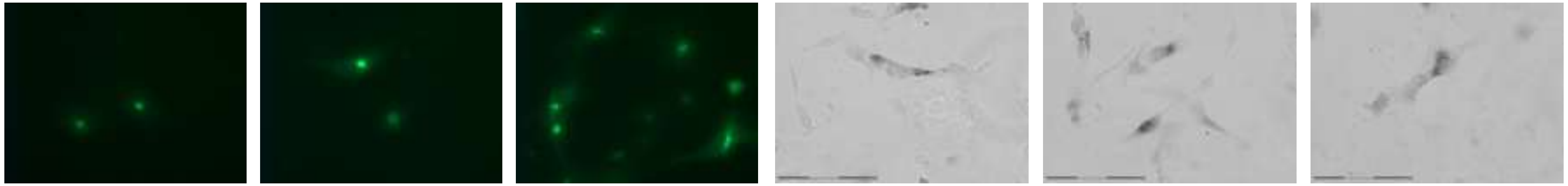

# 390V (MBD4)

Normal expression; reduced activity

wt

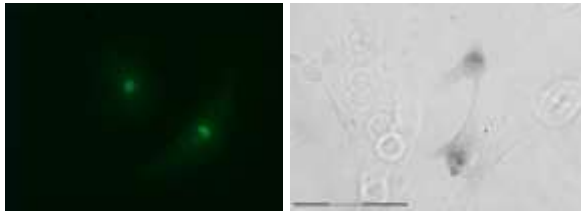

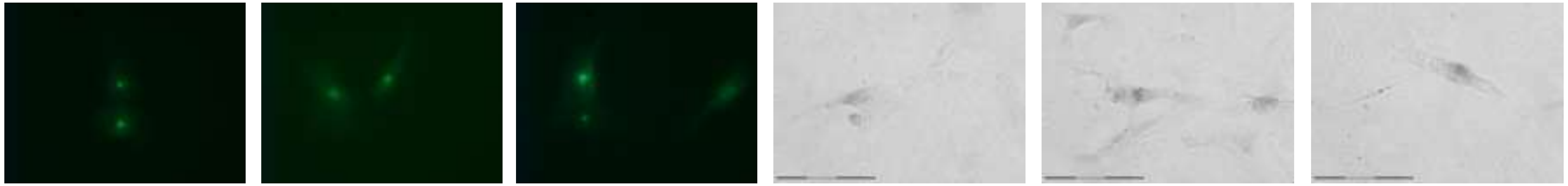

wt

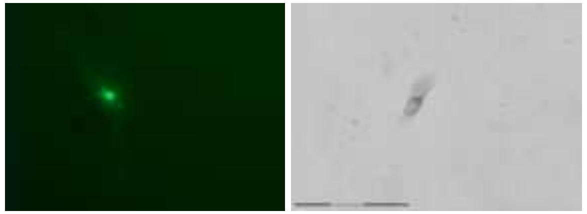

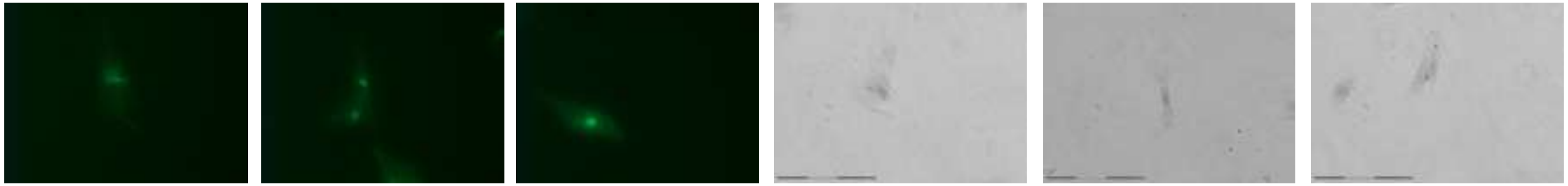

# E316K (MBD3)

Normal expression; significant activity

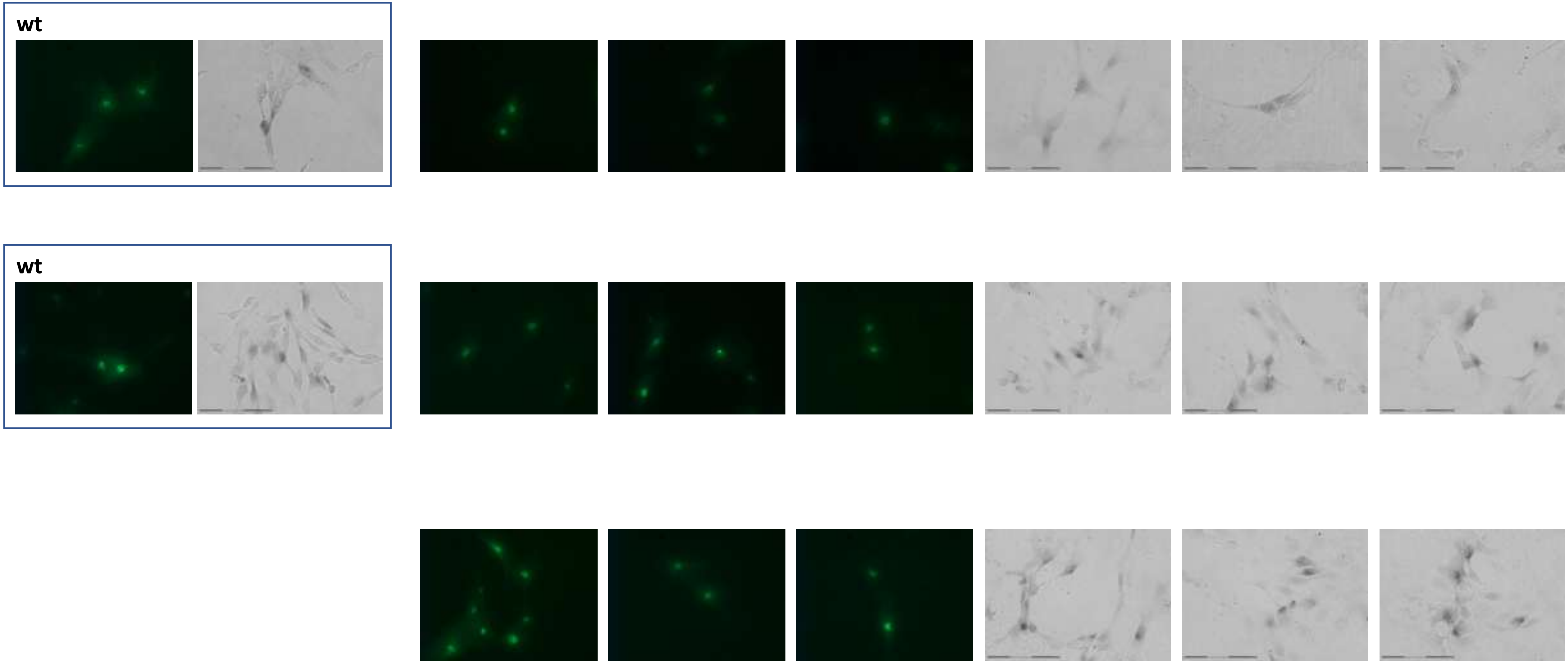

# A297S (MBD3)

Normal expression; significant activity

wt

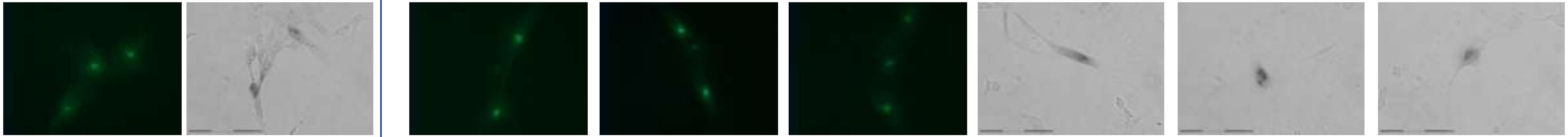

wt

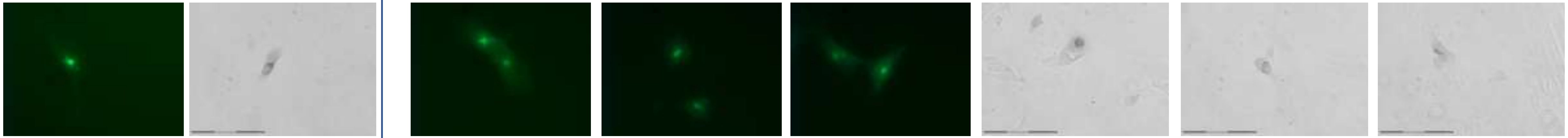

# L292S (MBD3)

**Normal expression; reduced activity**

**wt**

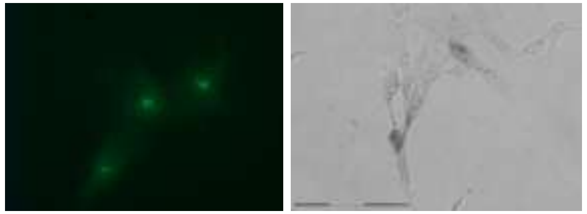

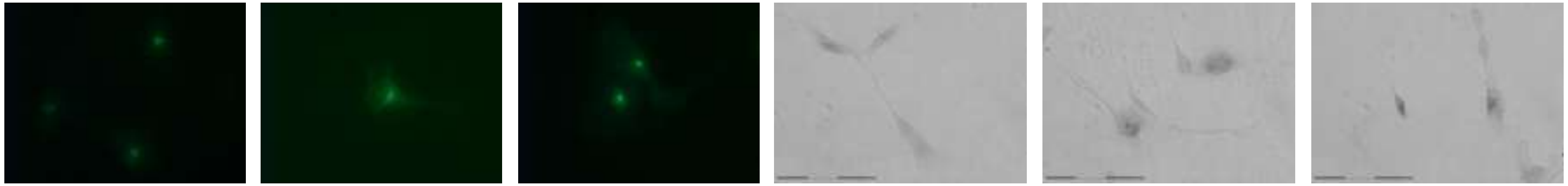

**wt**

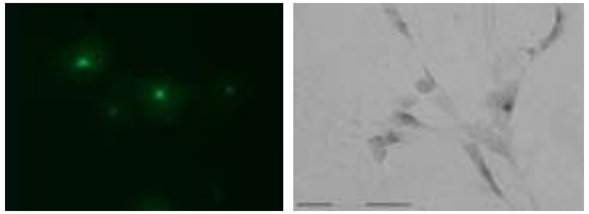

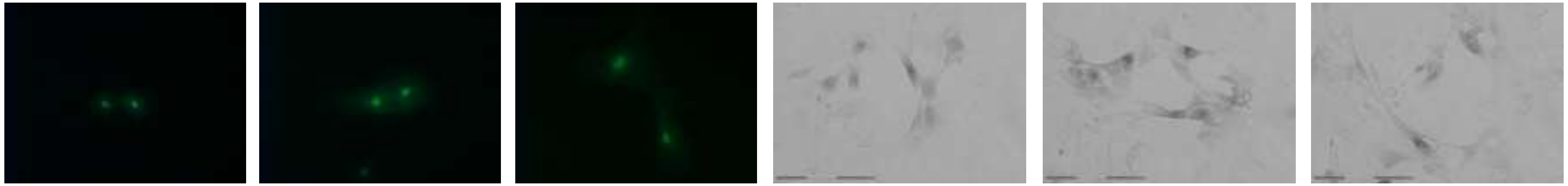

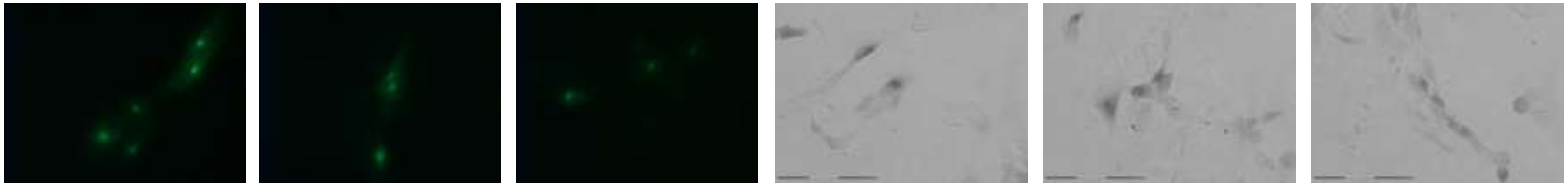

# G250R (MBD2,3 linker)

Normal expression; significant activity

wt

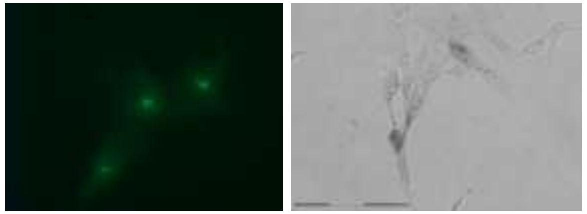

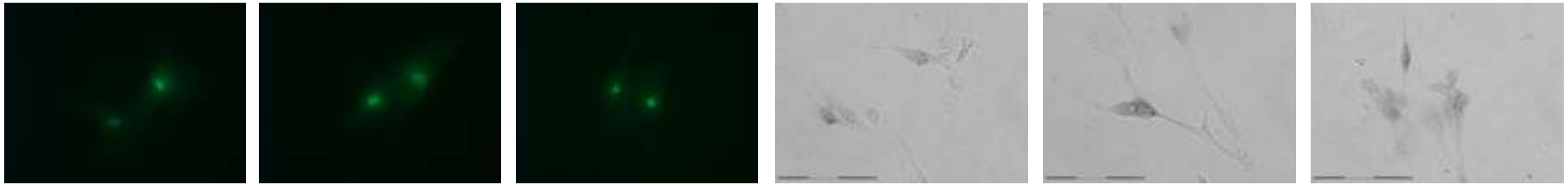

wt

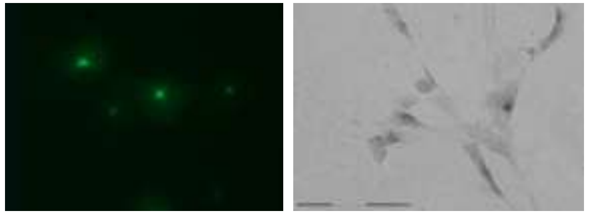

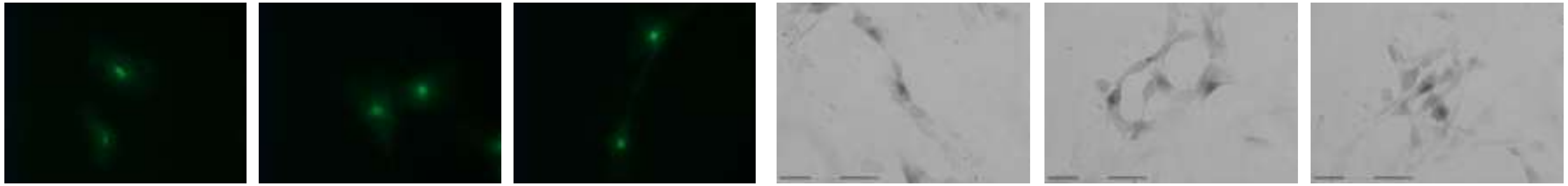

# R226W (MBD2,3 linker)

**Reduced expression; reduced activity**

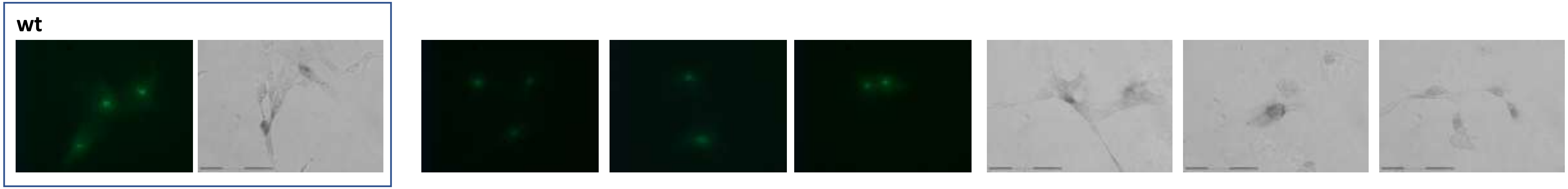


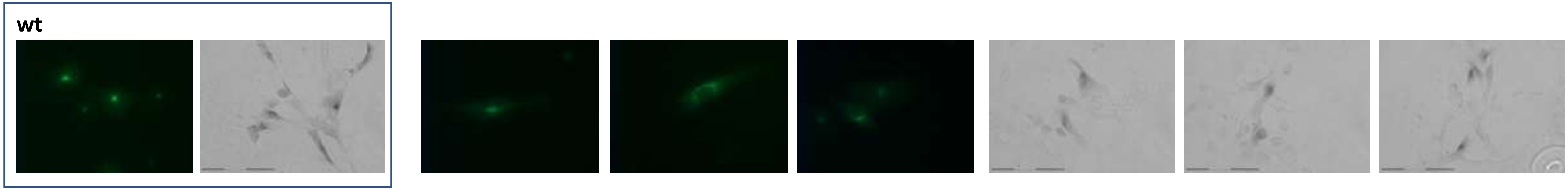

# I210V (MBD2)

**Reduced expression; reduced activity**

**wt**

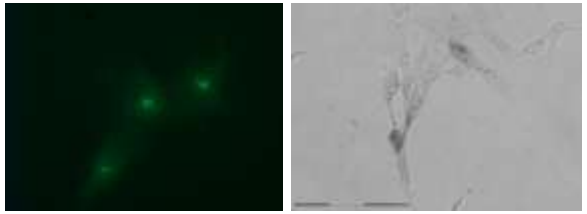

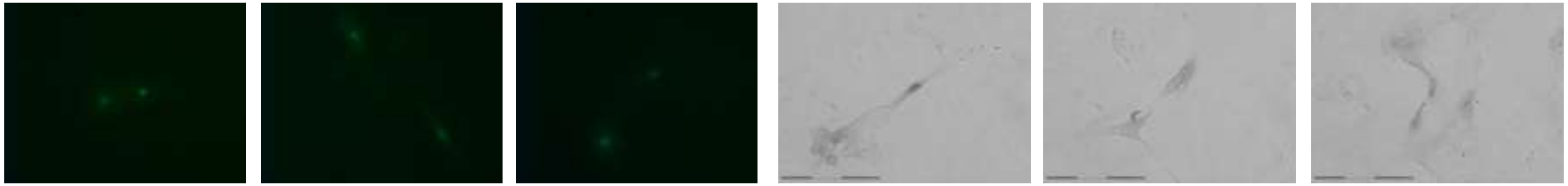

**wt**

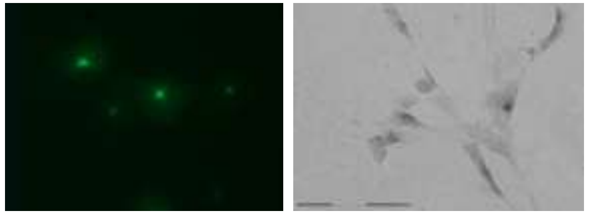

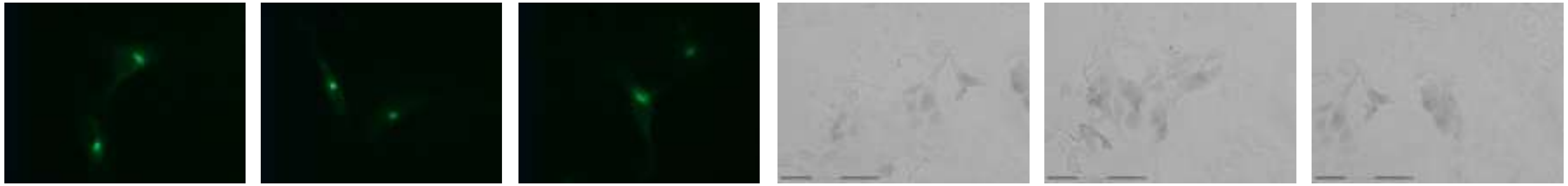

# D196E (MBD2)

**Normal expression; significant activity**

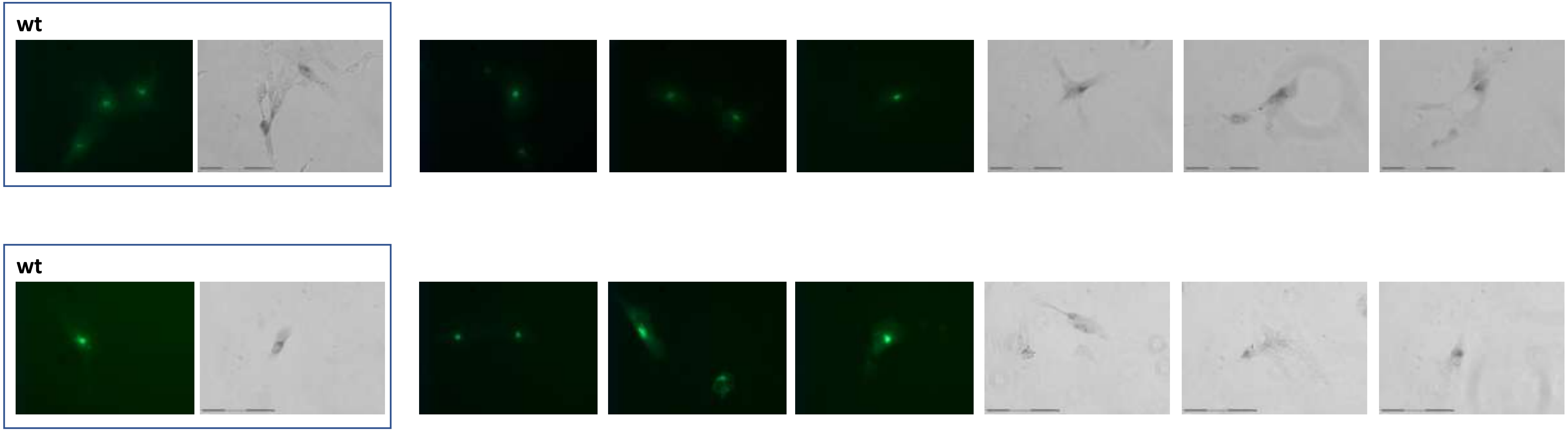

# A183V (MBD2)

**Normal expression; reduced activity**

**wt**

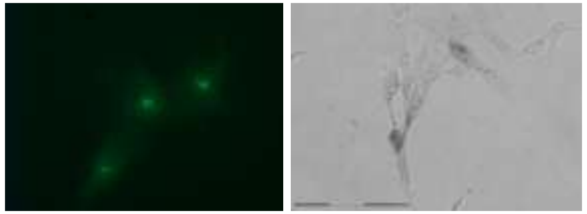

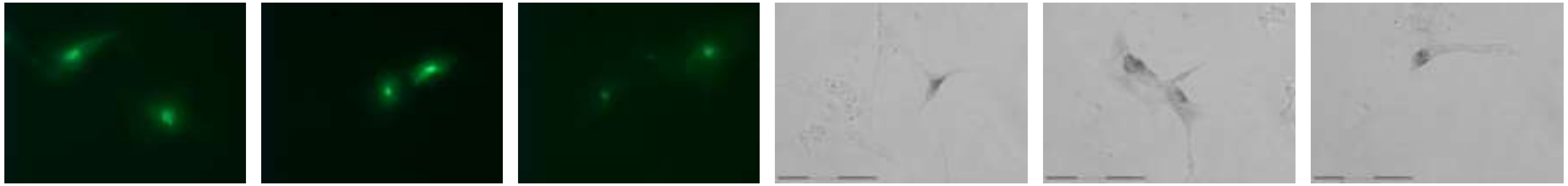

**wt**

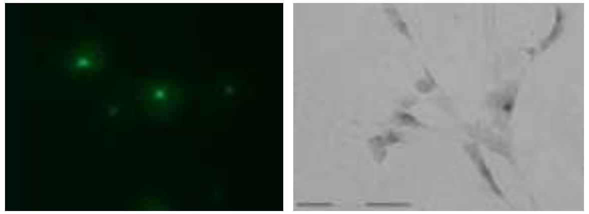

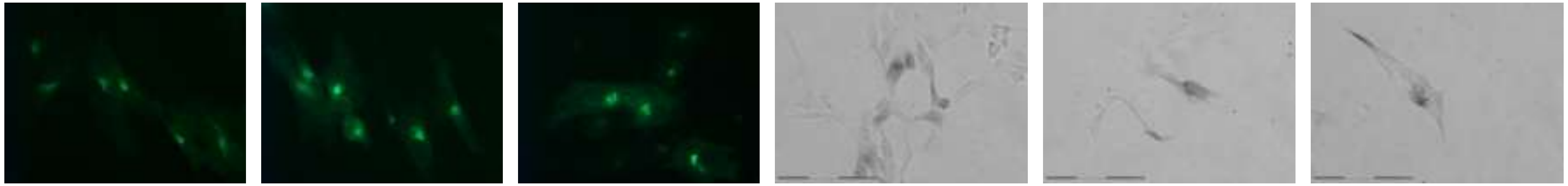

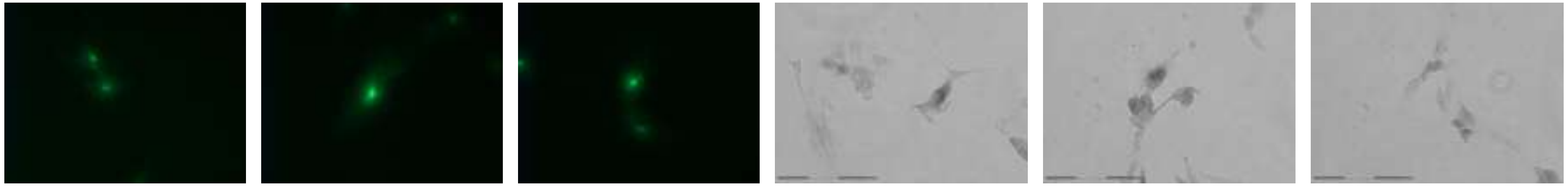

# L168P (MBD2)

**Reduced expression; reduced activity**

**wt**

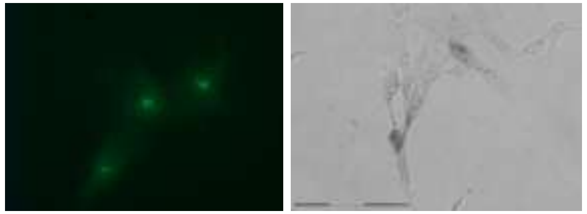

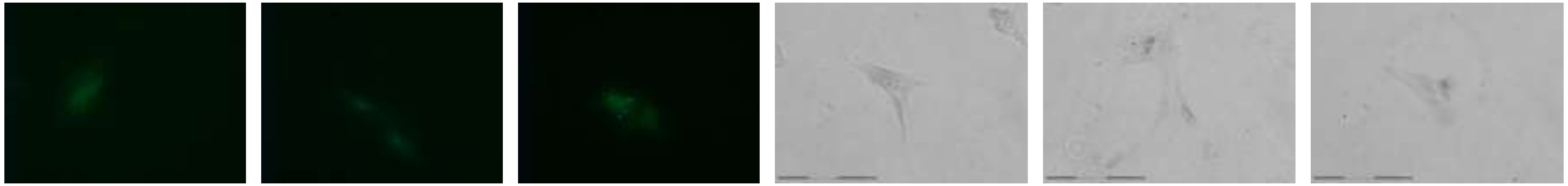

**wt**

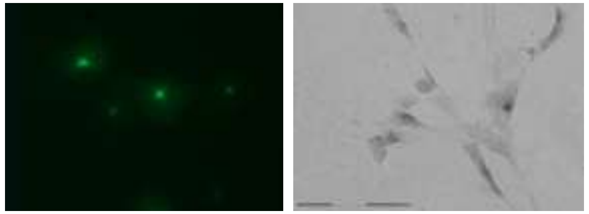

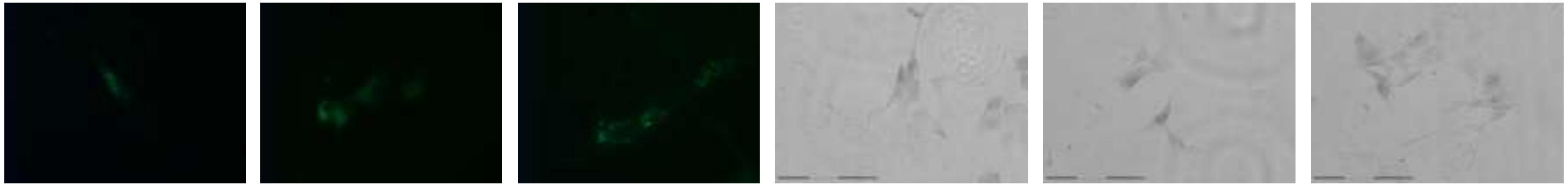

# R166W (MBD2)

**Normal expression; significant activity**

**wt**

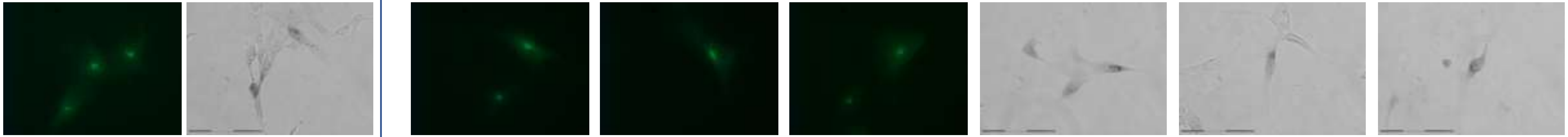

**wt**

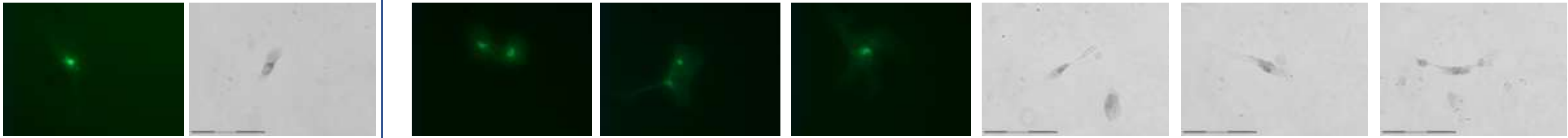

# I161T (MBD2)

**Reduced expression; reduced activity**

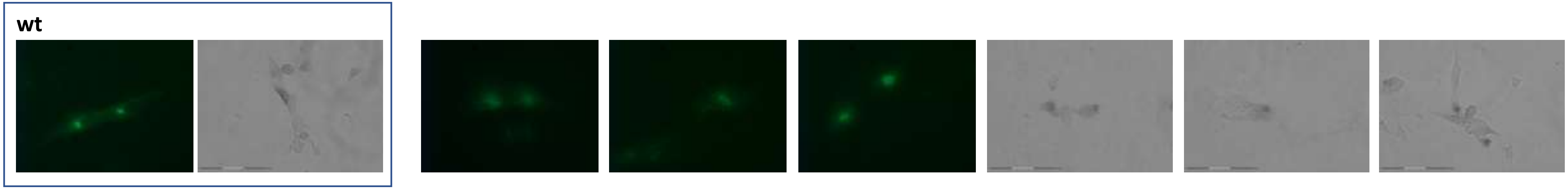


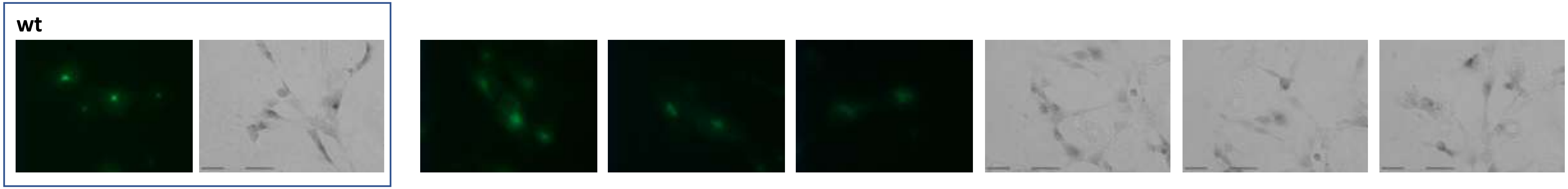

# V149M (MBD2)

Normal expression; reduced activity

wt

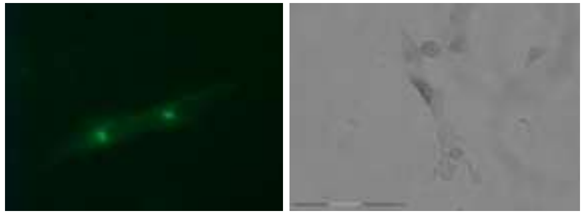

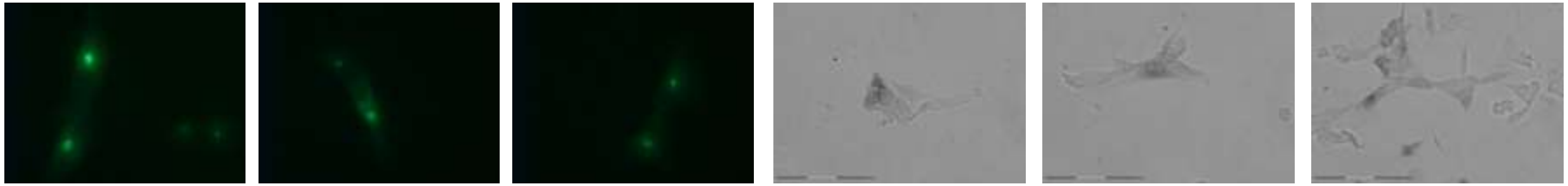

wt

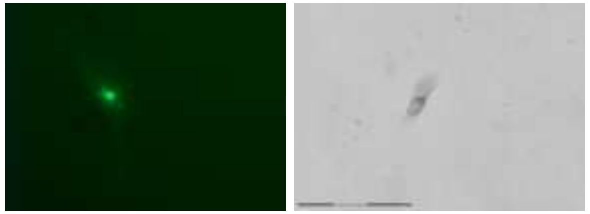

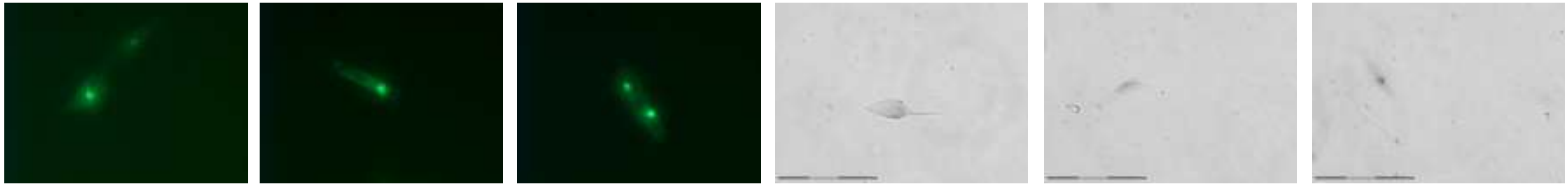

# R148W (MBD2)

**Normal expression; reduced activity**

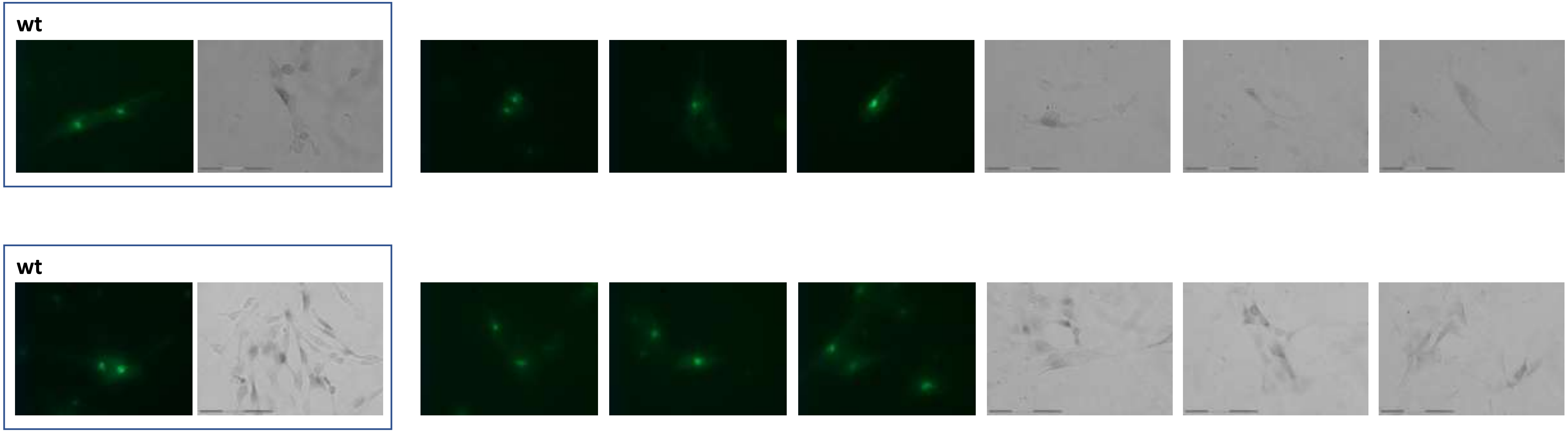

# R136G (MBD1,2 linker)

**Normal expression; significant activity**

**wt**

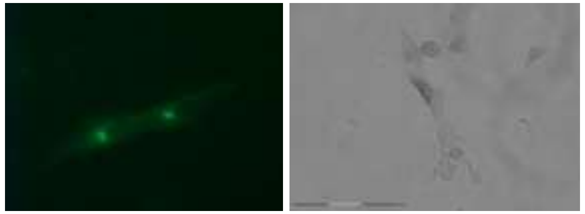

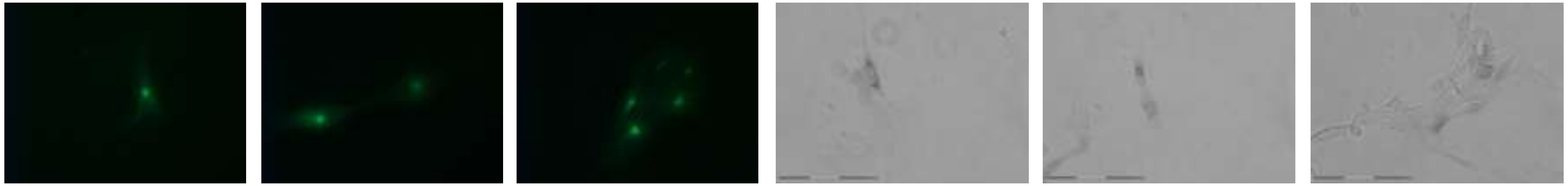

**wt**

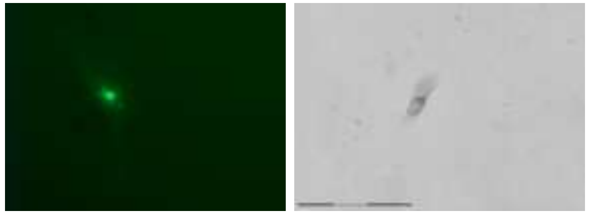

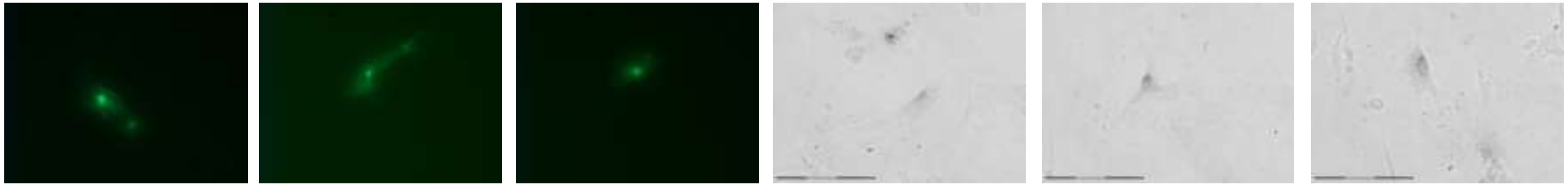

# S132F (MBD1,2 linker)

**Reduced expression; reduced activity**

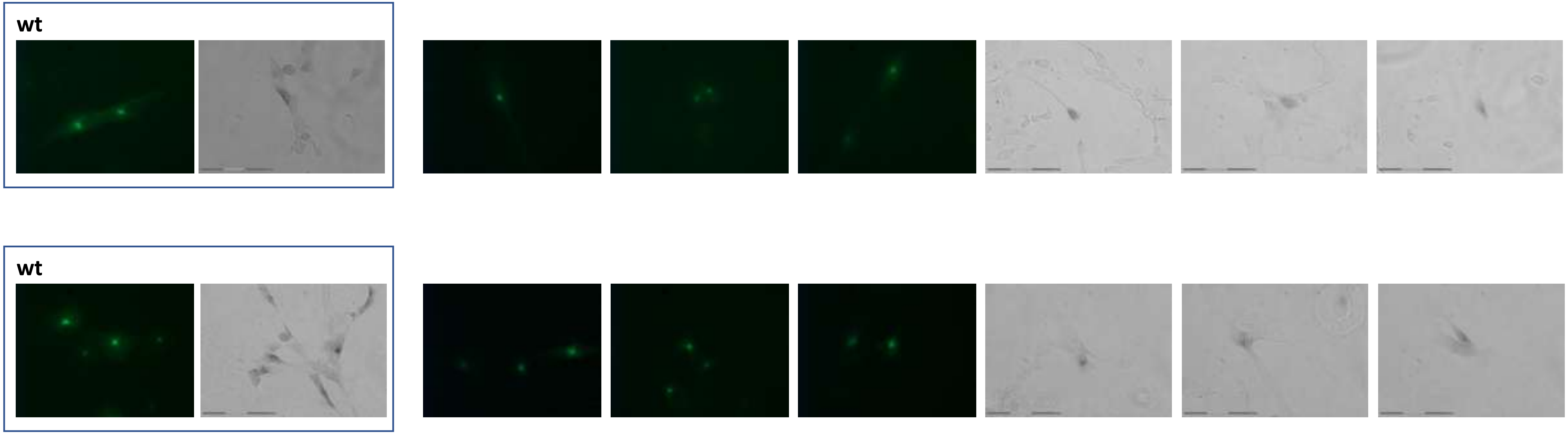

# I116T (MBD1)

Normal expression; significant activity

wt

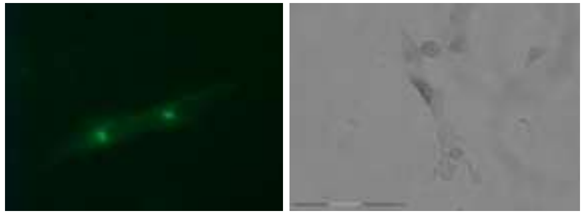

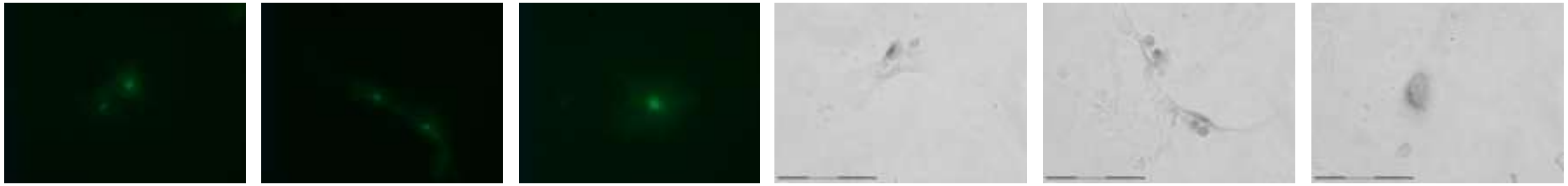

wt

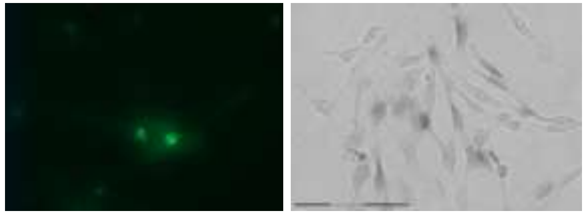

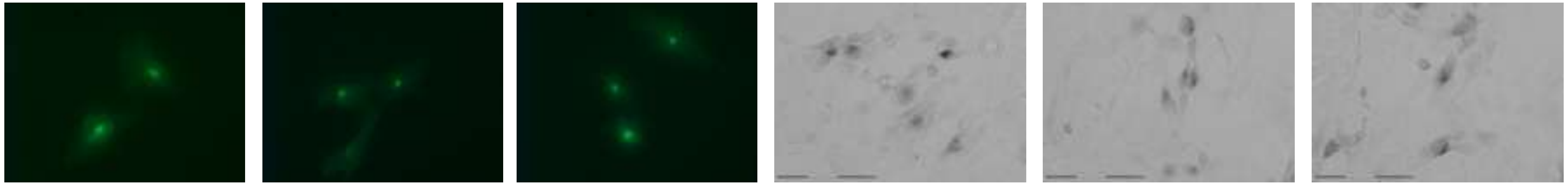

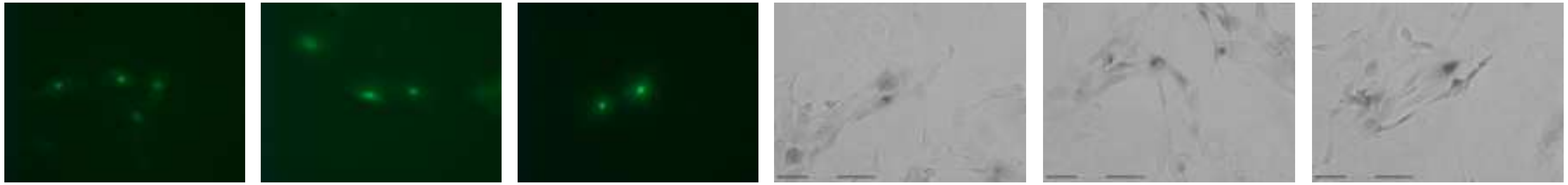

# N41S (N-terminal peptide)

Normal expression; significant activity

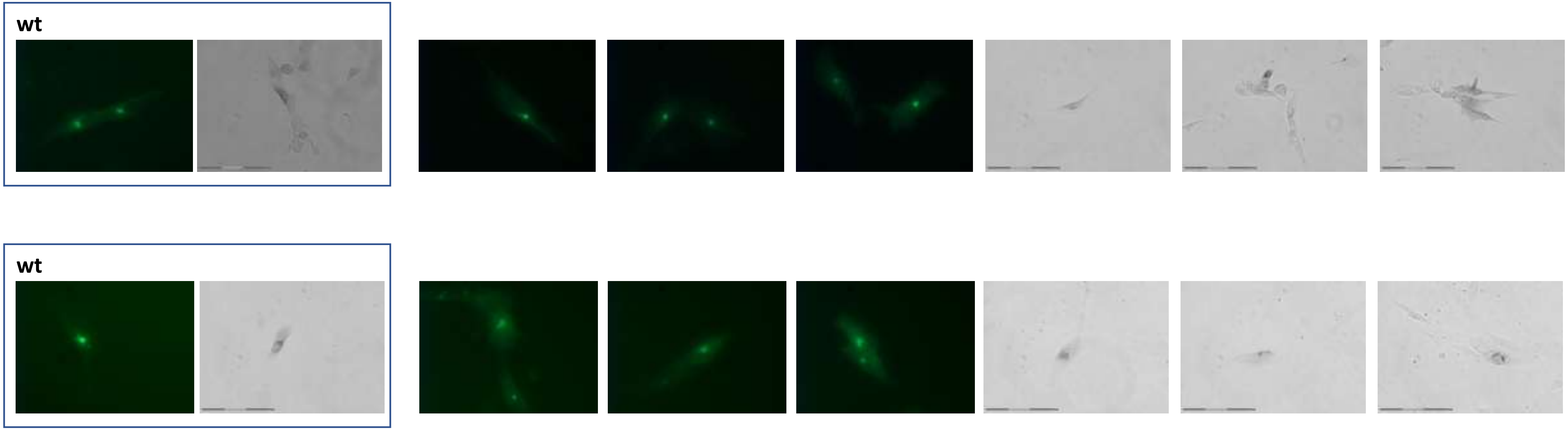

# D1027A

Normal expression; no activity

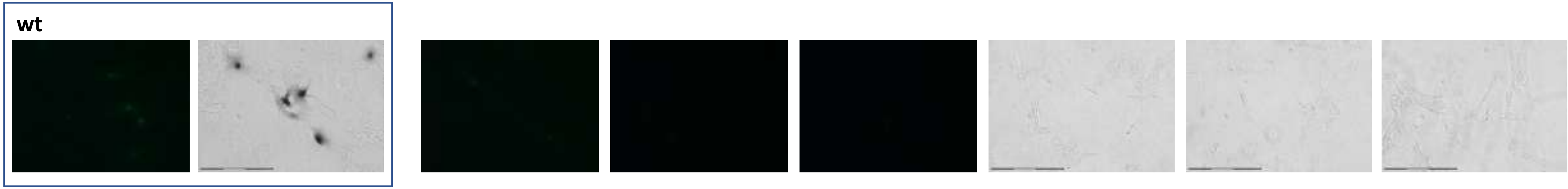

**A**

| ATP7B / ortholog | PDB ID | Resolution (Å) | Description | Reference |
|---|---|---|---|---|
| | 7SI3 | 3.2 | E2·$P_i$ state, consensus structure | |
| *Xt*ATP7B | 7SI6 | 3.3 | E2·$P_i$ state, MBD5 and MBD6 visible | Bitter *et al.*, 2022 |
| | 7SI7 | 3.5 | E2·$P_i$ state, MBD5 and MBD6 absent | |
| | 7XUM | 3.8 | C983S/C985S/D1027A mutant with $Cu^+$ in presence of ATOX1 (no ATOX1 density) | |
| | 7XUK | 3.3 | C983S/C985S/D1027A mutant in presence of ATOX1 (no ATOX1 density, apo structure) | |
| *Hs*ATP7B | 7XUN | 3.4 | Apo C983S/C985S/D1027A mutant | Yang *et al.*, 2023 |
| | 7XUO | 3.6 | C983S/C985S/D1027A mutant with cisplatin in presence of ATOX1 (no ATOX1 density) | |
| | 8IOY | 4.0 | C983S/C985S/D1027A mutant with AMP-PNP | |
| | 8Q73 | 3.6 | Apo E1 state | |
| *Os*HMA4 | 8Q74 | 3.7 | E1 state with $Cu^+$ | Guo *et al.*, 2024 |
| | 8Q75 | 3.2 | E2P state with $AlF_x$ | |
| | 8Q76 | 3.3 | E2P state with $BeF_x$ | |

**B**

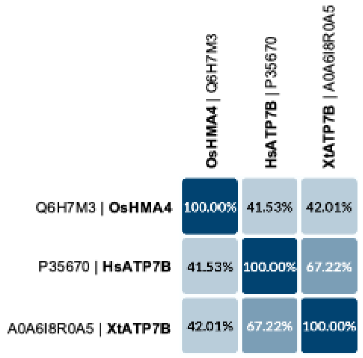


**Supplemental Figure 3. Available structural information for ATP7B**
**(A)** Available experimental structures of ATP7B and orthologs. Source organisms: *Xt*ATP7B - *Xenopus tropicalis* (frog), *Hs*ATP7B - *Homo sapiens* (human), *Os*HMA4 - *Oryza sativa* subsp. *japonica* (rice) **(B)** Sequence identity matrix of available experimental structures of ATP7B and its orthologs, created in UniProt using Clustal2.1 algorithm (UniProt ID | **ATP7B (ortholog)** label format).

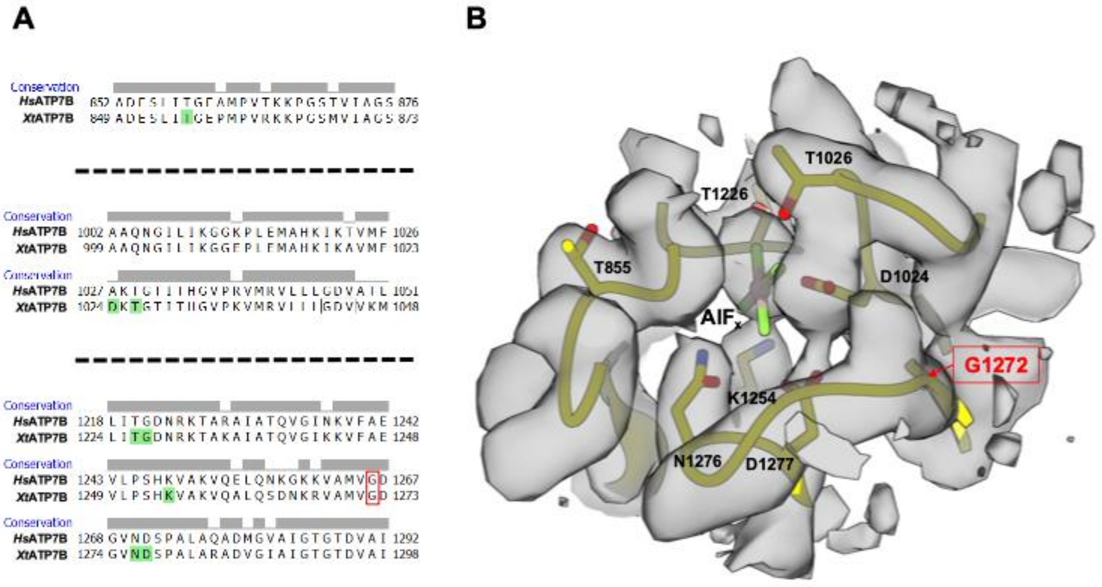


**Supplemental Figure 4 (A)** Amino acid sequences of human (*Hs*ATP7B, PDB ID 7XUN) and frog (*Xt*ATP7B, PDB ID 7SI3) ATP7B aligned in ChimeraX v1.8 using Clustal Omega v1.1.0 algorithm. Residues at 4Å-distance from $AlF_x$ (inorganic phosphate mimic) in 7SI3 model of *Xt*ATP7B, as visualized in **B**, are highlighted in green. 7SI3 model was used for predicting impact of G1266R variant in *Hs*ATP7B (analogous residue in *Xt*ATP7B, G1272, is highlighted in red/red box). All available experimental *Hs*ATP7B represent C983S/C985S/D1027A mutant. **B:** Cryo-EM density of *Xt*ATP7B (PDB ID 7SI3, resolution 3.19 Å) active site to demonstrate overall model quality in the region of interest. Residues at 4Å-distance from $AlF_x$ are shown as sticks. Residue G1272 in *Xt*ATP7B (labelled in red box) corresponds to G1266 in *Hs*ATP7B

```
                10         20         30         40         50         60
MBD1  atstvrilgm tcqscvksie drisnlkgii smkvsleqgs atvkyvpsvv clqqvchqig dmgfeasi
MBD2  avvklrvegm tcqscvssie gkvrklqgvv rvkvslsnqe avityqpyli qpedlrdhvn dmgfeaai
MBD3  vtlqlridgm hckscvlnie enigqllgvq siqvslenkt aqvkydpsct spvalqraie alppgnfk
MBD4  sttliaiagm tcascvhsie gmisqlegvq qisvslaegt atvlynpsvi speelraaie dmgfeasv
MBD5  qkcflqikgm tcascvsnie rnlqkeagvl svlvalmagk aeikydpevi qpleiaqfiq dlgfeaav
MBD6  gnieltitgm tcascvhnie skltrtngit yasvalatsk alvkfdpeii gprdiikiie eigfhasl
              ** ** ***  **        *      * *     *     *            *    ** *
```

**Supplemental Figure 5.** Sequence alignment of MBDs1-6. Residues in white are CXXC motif. Residues in red are mutated in selected WD-causing variants. Asterisks indicate high degree of conservation (7)
